# Assessing Arboreal Adaptations of Bird Antecedents: Testing the Ecological Setting of the Origin of the Avian Flight Stroke

**DOI:** 10.1371/journal.pone.0022292

**Published:** 2011-08-09

**Authors:** T. Alexander Dececchi, Hans C. E. Larsson

**Affiliations:** Redpath Museum, McGill University, Montreal, Quebec, Canada; Raymond M. Alf Museum of Paleontology, United States of America

## Abstract

The origin of avian flight is a classic macroevolutionary transition with research spanning over a century. Two competing models explaining this locomotory transition have been discussed for decades: ground up versus trees down. Although it is impossible to directly test either of these theories, it is possible to test one of the requirements for the trees-down model, that of an arboreal paravian. We test for arboreality in non-avian theropods and early birds with comparisons to extant avian, mammalian, and reptilian scansors and climbers using a comprehensive set of morphological characters. Non-avian theropods, including the small, feathered deinonychosaurs, and *Archaeopteryx*, consistently and significantly cluster with fully terrestrial extant mammals and ground-based birds, such as ratites. Basal birds, more advanced than *Archaeopteryx*, cluster with extant perching ground-foraging birds. Evolutionary trends immediately prior to the origin of birds indicate skeletal adaptations opposite that expected for arboreal climbers. Results reject an arboreal capacity for the avian stem lineage, thus lending no support for the trees-down model. Support for a fully terrestrial ecology and origin of the avian flight stroke has broad implications for the origin of powered flight for this clade. A terrestrial origin for the avian flight stroke challenges the need for an intermediate gliding phase, presents the best resolved series of the evolution of vertebrate powered flight, and may differ fundamentally from the origin of bat and pterosaur flight, whose antecedents have been postulated to have been arboreal and gliding.

## Introduction

The origin of avian flight has long been considered a classic macroevolutionary transition [1]. The ecological setting of this transition is significant because it influences the evolutionary drivers for critical components of this transition, such as the flight stroke or flight feathers. Initially, the argument for the origin of avian flight hinged on two competing scenarios: the ground-up and trees-down hypotheses. This dichotomy was originally based on debates of the phylogenetic relationship of birds to other archosaurs. The trees-down proponents favoured a “thecodont” antecedent and the ground-up supporters championed a theropod dinosaur origin of birds (see [2] for a review). A theropod ancestry of birds was first proposed by Huxley [3], resurrected by Ostrom [4], and is supported by an overwhelming wealth of new fossils and phylogenetic analyses in recent years (see [5], [6] for brief reviews of supporting data). In spite of the dinosaurian ancestry of birds, the debate on the ecological setting of the origin of avian flight is still in flux. The plesiomorphic state for non-avian theropods is undoubtedly terrestrial [7], but the possibility of tree-dwelling, small-bodied theropods closely related to birds has resurrected intense debates that bird antecedents may have been arboreal and gliding [8], [9].

The ground-up scenario implies that the capacity for powered flight evolved from a fully terrestrial theropod precursor. This hypothesis suggests feathered limbs evolved for a non-locomotor function, such as display or insulation, and the flight stroke developed to aid in high speed running or traversing steep inclines [2], [10], [11]. The trees-down scenario implies that powered flight evolved in an arboreal lineage of theropods, where, feathered limbs were selected for increased surface area and functioned primarily for parachuting and gliding [12]–[14].

Compelling cases for an arboreal origin of flight have been made based on body size, feather placement, and pedal claw geometry of new, small, feathered, non-avian theropods [15]–[17]. Equally convincing arguments for a terrestrial origin of flight have been made with corrections to older methods of claw geometrics [18], lack of a reversed perching hallux [19]–[21], and feasible biomechanical models of flight from high-speed running [10]. The apparent dichotomy of the ecological setting for the origin has been blurred, and perhaps made moot [22] with recent discoveries that many extant birds have a peculiar behaviour called wing assisted incline running (WAIR) [11], [23]. WAIR is used by extant birds to ascend steeply inclined, vertical, and even slight overhanging surfaces with the aid of a powerful flight stroke. Although this behaviour allows access into trees, WAIR is essentially a specialized form of terrestrial locomotion that is related to the running-flapping model [10].

Although the arboreal-terrestrial dichotomy may be less clear cut than originally proposed, many recent authors have argued for a trees-down, gliding model based on a arboreal, climbing bird antecedent [8], [9], [13], [14], [24] and that the flight stroke was derived either simultaneously or shortly after from wing-assisted descents from trees [8]. An arboreal context for the origin of the flight stroke requires avian antecedents to have been tree-dwelling. Extant arboreal vertebrates have a suite of adaptations different from their terrestrial counterparts to aid in moving along thin diameter branches, such as large phalangeal indices and extremely mobile shoulder/hip and wrist/ankle joints [25]. An arboreal origin of the flight stroke is expected to be tied with a suite of arboreal adaptations. Although WAIR could have facilitated ascent into trees, it does nothing to aid in movement along branches or descent. Any arboreal setting for the origin of flight, preceded by either a climbing and gliding pathway or a terrestrial-based WAIR origin of the flight stroke and access to the trees, is expected to present some degree of arboreal adaptations in avian antecedents. Conversely, a terrestrial origin of the flight stroke is not expected to have any trace of arboreal adaptations.

To better understand the context of the origin of the flight stroke and powered flight in birds, we need knowledge of the functional, behavioural, and ecological repertoire available to the antecedents of the first fliers. In this paper, we tested for evidence of arboreality in bird ancestors. The trees-down hypothesis is predicated on the existence a lineage of highly arboreal theropods preceding the evolution of the aerofoil and gliding locomotion [8], [12], [14], [24]. We examined anatomical support for arboreality in non-avian theropods using a suite of well-known morphological characters relevant to arboreal locomotion in extant taxa, rather than focusing on any single trait. Given that the plesiomorphic locomotory state for Theropoda is terrestrial cursoriality, we will start with the assumption that all non-avian theropods are terrestrial. If morphological adaptations to facilitate movement into, out of, and within trees are absent, we can argue against an arboreal ancestry of birds and, therefore, the trees down hypothesis.

The sister taxon to Aves is either Deinonychosauria (composed of Dromeaosauridae and Troodontidae) or the enigmatic Scansoriopterigidea [26], [27]. It is within these groups that one would expect the evolution of anatomical traits to facilitate and refine climbing, descending, and branch-walking locomotion. We selected morphological characters that are associated with a clear functional role in arboreal locomotion and/or climbing and are widely distributed across extant and extinct tree dwellers including some pterosaurs, non-mammalian therapsids, mammals, birds and other reptiles [28]–[32].

## Materials and Methods

### Ethics statement

No live animals were used in this study.

### Categorizing arboreal and terrestrial taxa

Occasional climbers, require little behavioural or morphological adaptations. However, similar to the ability to swim, these non-specialized taxa show little propensity in the act and are at greater risk of injury from falling [25], [33]. Truly arboreal tetrapods, or those for whom a scansorial lifestyle occupies a large portion of their foraging regimes, have convergent solutions to the twin problem of securing themselves while maintaining manoeuvrability [25], [32], [34]–[36]. Today's arboreal vertebrates include quadrupedal salamanders, frogs, mammals, and lizards, bipedal birds, and limbless snakes. We compiled data for extant quadrupedal and bipedal taxa to encompass the complete range of potential locomotory modes of non-avian theropods within an arboreal environment.

We focused on claw and grip-based climbing adaptations because non-avian theropods show no evidence for more specialized climbing methods such as suction, capillary action, or dry adhesion that are present in climbing salamanders, frogs, and many lizards. We followed the categorization of arboreal, scansorial, and terrestrial of Van Valkenburgh [37] for non-bird taxa. Arboreal is defined as “[r]arely on the ground, forages and shelters in the trees”; scansorial is “[c]apable of climbing, usually climbs for escape”; and terrestrial is “[r]arely or never [c]limbs…” Although these categories have indiscrete boundaries, they provide straightforward definitions that are in general use. These categories allow us to clearly define endpoints while attempting to account for the continuum of the transition from primarily terrestrial (e.g. the horse), occasional climbers (e.g. the housecat) to those that primarily live in the trees, but are capable of foraging on the ground (e.g. the grey squirrel). We did not differentiate between subsets of locomotion within each category (i.e. cursorial and fossorial taxa were included in terrestrial).

Five foraging categories were used for avian taxa based on Glen and Bennet [18] with some modifications. These consist of ground based, ground foragers (Gg and Ga of [18]), aerial foragers (A) (Ag and Aa of Glen and Bennet [18]), climbers, and birds of prey. With the exception of ground based birds, which rarely or never roust or perch in trees, the majority of living birds spend at least part of their lives in trees. Even taxa that forage primarily on the ground require some arboreal adaptations.

Due to the uncertainty involved in the possible stance adopted by theropods within an arboreal setting, either retaining the bipedal one common to all terrestrial forms [7], or using a more quadrupedal stance, we ran multiple analyses to test all possible permutation. These included a “naive” analysis using the entire dataset (including both bipedal birds and quadrupedal taxa, regardless of stance) and separate bipedal and quadrupedal permutations. Due to bipedalism being plesiomorphic for theropods [7], this stance allowed the forelimbs of theropods to evolve without the constraints involved in locomotion. Because the forelimbs of many theropods were likely used for prey capture [38]–[41], and characters associated with predation and climbing often overlap [42]–[44], the predatory nature of theropods could influence our analysis and give a false positive for climbing. To attempt to minimize this possibility we also performed forelimb and hindlimb only analyses. This division of fore- and hindlimbs is also warranted as it has been suggested that climbing adaptations often manifest themselves primarily in the hindlimb [25], [45]. This is due, in grip based climbers, to the necessity to secure a safe purchase while reaching for new supports [25], [46], [47] and in claw based climbing specialists in the evolution of reversible hindfeet and head first descent [25]. Thus, we suspect that the hindlimb should be more informative in assessing arboreality than the forelimb in theropods, necessitating separate analyses.

### Taxa

Seventy-four extant mammals, nine lizards (including two chameleons and the extinct glider *Xuanlong*), and three extinct arboreal taxa (the synapsid *Suminia* and the enigmatic drepanosaur diapsids *Megalancosaurus* and *Vallesaurus*) were used to represent quadrupeds and scored for all characters. Although lizard locomotion differs fundamentally from that of theropods, the inclusion of a sample of sprawling lizards (including two “flying” lizards, the extant genus *Draco* and the extinct *Xianlong*) was done to ensure a diversity of climbing styles was represented in our study. The sprawling gait of lizards (with the exception of the chameleon) is fundamentally different in terms of loading mechanics, limb retraction, and kinematics during locomotion from the parasagittal gait found in mammals, birds and theropods [48], [49]. These factors, combined with the general small body size of lizards (>80% of taxa are less than 50 g [50] compared to paravians (∼700–2000 g [51]) and the evolution in highly arboreal species with toe pads to enhance grip [52]–[54], generally make lizards unsuitable analogs for theropod behaviour or locomotion. Most lizards, including all used in this analysis, are scansorial and often climb rocks, trees, and bushes [55]. We included only the chameleon in the analyses discussed here because it is the most arboreal of all lizards and has a unique parasagittal stride [56] similar to that expected in an arboreal bird antecedents.

Due to the high degree of morphological disparity between birds and other tetrapods and the bipedal terrestrial and arboreal locomotion of birds, a separate analysis was performed using thirty-one avian taxa that sample each of the five foraging categories of extant birds. Although the absolute value of some metrics may not be directly comparable to quadrupeds, similar trends should be repeated in non-avian theropods as a general solution to the problems of ascending, moving within, and descending from trees.

Twenty-one well preserved theropod specimens, representing fifteen different genera, were included to examine how they clustered with the extant groups. Quantitative and qualitative data of non-avian and avian theropods were taken directly from museum specimens and the literature. A dataset of the most complete non-avian and early avian theropod taxa was used in the cluster analysis. Additional, less complete, non-avian theropods were used for individual quantitative indices to gather the widest phylogenetic and body size ranges of these taxa. Measurements of *Epidendrosaurus* are suspect given this taxon's early ontogenetic stage [17] (pers. obs.) but included for completeness.

### Qualifying and quantifying arboreal adaptations

Seventeen discrete characters diagnostic for habitat preference were used to compare both non-avian theropods and basal birds to non-avian tetrapods. These characters have been demonstrated to be indicators of increasing arboreality [25], [42], [57]. These included the presence of an opposable hallux and/or pollex, the ability of the tail to act as a prehensile organ or as a support on a vertical surface, the ability to pronate/supinate the forelimb, hindfoot reversal and claw curvature. A set of commonly used quantitative indices were applied including the brachial index (BI, ulnar length/humeral length), crural index (CI, tibial length/femoral length), manual and pedal phalangeal indices (MPI and PPP, non-ungual length of the digital ray length/metapodial length), and overall limb lengths. Overall limb lengths were calculated from stylopodial (humerus/femur) and zeugopodial (ulna/tibia) segment lengths divided by trunk length. Only these two limb segments were used to maintain a common comparator between plantigrade to digitigrade taxa. CI does not have the same functional relationship in birds as it does in non-avian tetrapods because of the horizontal position of the femur and different hindlimb biomechanics, and bone proportions in living birds [58]–[60]. The avian tarsometatarus length was divided by the tibial length to derive a more comparable index of distal segment elongation for use in the combined dataset in all extant avians and advanced fossil birds (i.e. Ornithothoraces). We also performed the analyses using the “traditional” CI index for both the advanced fossil and extant birds, and it does not significantly alter the results (results not shown). Qualitative multistate characters were used to characterize joint mobility variation. We define low mobility as movement restricted to a single plane, or allowing very limited active movement in multiple planes (e.g. the ankle of a horse). Medium mobility is defined as movement in more than one plane, but an inability to fully abduct/adduct or invert/evert that segment without discomfort (e.g. wrist of the house cat). Highly mobile joints are defined as those that can freely and fully abduct/adduct and even circumduct (e.g. the wrist of tree squirrels).

Eight characters associated with climbing and perching abilities in extant birds were used to compare extinct theropods to their living descendents [61]–[63]. In addition to PPI and claw curvature, these characters include relative hindlimb, tibial, and metatarsal length (standardized to body mass), the presence and extent of a reversed hallux, presence of zygodactyly, and any modification of tail feathers to act as a supporting strut. Because of the differences in non-avian theropods and bird hindlimbs, tibial and metatarsal indices were standardized against the mean of each clade to generate comparable values. Mass values for non-avian theropods were calculated based on femoral length or circumference estimates, with the lowest value chosen.

These metrics can be divided into those that reduced the distance between the centre of mass and the substrate (i.e. BI), those that facilitate securing a purchase (i.e. claw curvature, PPI) and those that permit greater mobility (i.e. joint flexibility characters). We substituted the functional homologue Ph.III-I for the central metatarsus when computing PPI, as suggested in Hopson [63] because of the digitigrade stance of the theropod foot.

The musculoskeletal characters used in our analyses were selected, a priori, to be not restricted to any particular taxonomic group and show a broad distribution and association with arboreality in unrelated extant clades. The dissociation between these characters and phylogeny is demonstrated by the lack of phylogenetic signal in the clustering results. Continuous characters were also used to examine general trends from terrestriality to arboreality, without regard to absolute values. The patterns of these trends are also non-phylogenetic because they are repeated across unrelated lineages in response to arboreal demands and only used to derive qualitative trends from assemblages of unrelated clades. Correlation cluster algorithms, principal coordinate analyses (PCO), and linear regressions were performed in PAST v.2.00 (PAlaeontological STastics) [64] and R [65]. Complete data and statistical results are given in Table S1, S2, S3, S4, S5, S6, S7, S8, S9, S10, S11, S12, S13, S14 and Text S1.

## Results and Discussion

### Quantitative analyses

#### PCO and clustering analyses

**Figure 1:**
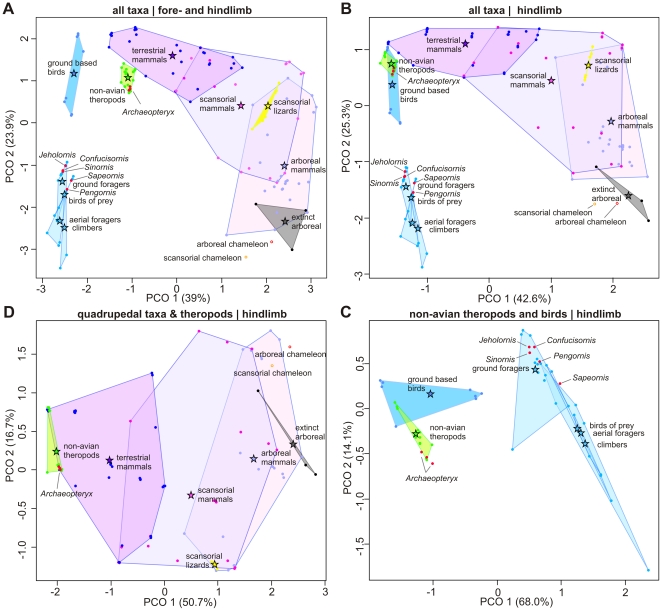
Plot of the first and second principal coordinates (PCO) of discrete locomotory traits. PCO values are calculated in Euclidean and presented for the total dataset of a selection of extant mammals, scansorial lizards, a scansorial and arboreal chameleon, and three extinct arboreal taxa (A), the total taxon set using only hindlimb morphologies (B), a partition of only quadrupedal mammals and reptiles with non-avian theropods and *Archaeopteryx* using only hindlimb morphologies (C), and a partition of only non-avian theropods and birds using a dataset tailored for bird morphologies (D). Each category of taxa are plotted within their respective convex hulls and category labels are given near each category's average, denoted by a star. Non-avian theropods are represented in green hulls, birds in blue hulls, mammals in purple hulls, scansorial lizards in yellow hulls, and fossil arboreal taxa in grey hulls. Basal Mesozoic birds are plotted as red filled circles. The variance explained by each PCO axis is given in parentheses after each axis label. [planned for page width].

**Figure 2:**
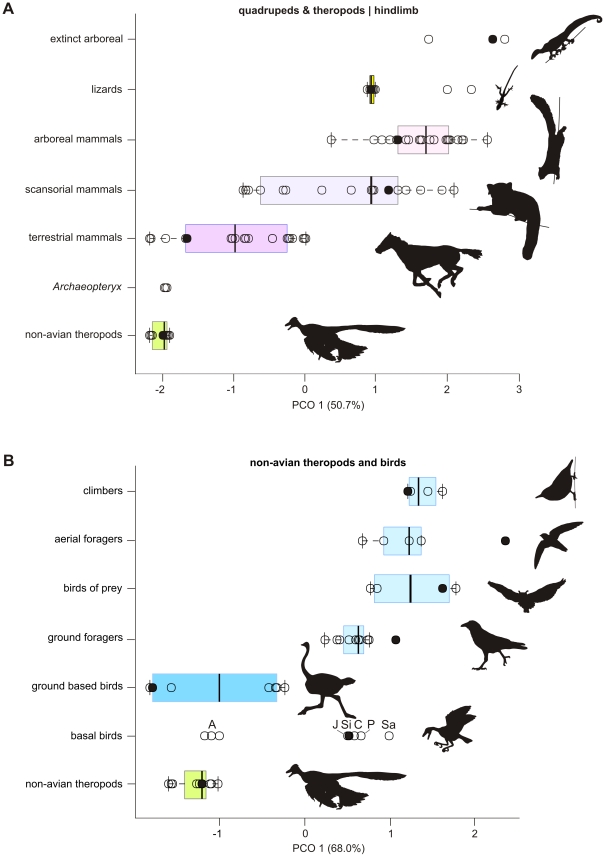
Box-plots of the first principal coordinate axis of discrete locomotory traits. (A) are extant quadrupedal mammals and reptiles compared to non-avian theropods and (B) extant birds to non-avian theropods and Mesozoic birds. PCO values are calculated in Euclidean. Note that non-avian theropods and *Archaeopteryx* cluster with terrestrial taxa at the extreme left of the graphs and have no overlap with scansorial or arboreal mammals and reptiles nor perching birds. The arboreal and scansorial chameleons are plotted to the right of the scansorial lizards. The variance explained by each PCO axis is given in parentheses after each axis label. Basal birds are labelled as: *Archaeopteryx*, A; *Confuciusornis*, C; *Jeholornis*, J; *Pengornis*, P; *Sapeornis*, Sa; *Sinornis*, Si. The filled circles represent positions for figured taxa. In (A), non-avian theropods = *Microraptor zhaoianus*, terrestrial = horse (*Equus*), scansorial = Red Panda (*Ailurus filgens*), arboreal = Grey Squirrel (*Sciurus carolinensis*), scansorial lizards – *Anolis carolinensis*, fossil arboreal – *Megalancosaurus*. In (B) non-avian theropods = *Microraptor zhaoianus*, basal birds = *Sinornis santensis*, ground based birds = Ostrich (*Struthio camelus*), ground foragers = Common Raven (*Corvus corax*), birds of prey = Great Horned Owl (*Bubo virginianus*), aerial foragers = Chimney Swift (*Chaetura pelagica*), climbers = Eurasian Nuthatch (*Sitta europaea*). Silhouettes of *Microrapor* and *Sinornis* are based on Hu and colleagues [27] and Sereno and Rao [33], respectively. Silhouettes are not to scale. [planned for page width].

All PCO and clustering analyses of the total and partitioned data revealed the same result; that non-avian theropods are most similar to extant terrestrial taxa (Figure 1, 2, S1, S2, S3, S4, S5, S6, S7, S8). In all PCO analyses, the first axis best discriminates terrestriality from arboreality in the quadrupedal and bird-specific datasets. It is noteworthy that all theropods, including all birds, are located in the same range of this axis as the most cursorial of mammals when examined together (Figure 1A, B). PCO plots readily separate terrestrial mammals and ground-based birds from their arboreal counterparts. There is some overlap between the terrestrial and scansorial mammal hulls, such as the terrestrially classified rat and scansorial mustelids, like the fisher and martin. The scansoreal and arboreal mammal hulls also partially overlap, with mammals such as the scansorial possum (*Didelphis*) with arboreal grip-based climbers like primates. As expected, all PCO and cluster analyses grouped lizards with scansorial and arboreal clawed-based climbers. In no analysis did lizards group with any non-avian theropod, bird, or cursorial mammal.

There is no overlap in the morphospaces of ground-based birds with all other bird groups when examined with both the quadrupedal and bipedal-specific morphological characters. When examined together, no bird or mammal group overlap, although ground-based birds and terrestrial mammals near each other at their most cursorial taxa, such as ratites and horses.

Non-avian theropods occupy a surprisingly small morphospace for their phylogenetic diversity and large body mass range. The morphospace of non-avian theropods maps within the terrestrial cursorial range of mammals in the total and quadrupedal-partitioned analyses. Non-avian theropods also map within the ground-based bird morphospace in the total and bird-partitioned analyses. *Archaeopteryx* always plots within the non-avian theropod morphospace. However, more derived Mesozoic birds cluster within the morphospace realm of perching birds, and most are positioned within the morphospace of the generalist, ground foraging birds, such as corvids and the European Starling (*Sturnus vulgaris*). These results support earlier proposals of the ecology of these basal birds [66]–[68].

Cluster analyses also group all non-avian theropods with terrestrial mammals and ground-based birds. Arboreal quadrupedal taxa are divided into two main groups: claw-based (sciurids and carnivores) and grip-based (primates, chameleon, marsupials, and the kinkajou) climbers. This division supports the hypothesis that each strategy enforces differential selection pressures. Claw-based climbers generally clustered closer to terrestrial and scansorial taxa, suggesting they are less specialized for arboreality. All non-avian theropods cluster within the terrestrial “cursorial” species grouping. When hindlimb and tail characters are partitioned from the total data, this partition yields a similar pattern, but with less resolution than the whole body dataset. As expected, if only the forelimbs of quadrupeds and non-avian theropods and *Archaeopteryx* are examined, the hulls of each group broadly overlap each other and there is little distinction between each group in the cluster analysis (Figure S9, S10). In general, non-avian theropods clustered with scansorial and grip-based climbing taxa, though the compsognathids and tyrannosaurs grouped with the lizards. This clustering is likely due to anatomical similarities between the predatory function of non-avian theropod forelimbs with grip-based climbers, such as the high MPI and a divergent pollex, both of which allow for enhanced gripping of small diameter objects or prey items.

#### Forelimb and hindlimb lengths

Relative fore- and hindlimb lengths of some extant arboreal taxa are significantly longer when compared to their terrestrial counterparts (Table S1). Much of this elongation is from the stylopodial bone, resulting in a reduced BI and CI, to create a long limb that effectively folds the stylopodium and zeugopodium together. Yet non-arboreal cursors and specialist jumpers also show significant hindlimb elongation but with elongated zeugopodial and metapodial bones. The purported “arboreal” theropods, *Microraptor*, *Anchiornis* and *Epidendrosaurus*, have significantly increased fore- and hindlimb indices when compared to other non-avian theropods (uneven t-test _t_forelimb = −10.971, P_(α = 0.05)_<0.0001, hindlimb = −3.4059, P = 0.005). This may be partly due to the small body size of these taxa as within theropods there is a strong negative correlation between trunk length and limb length (Dececchi and Larsson unpublished data). However, their elongate limbs are largely due to their elongated zeugopodial and metapodial bones, suggesting a rather cursorial mode of locomotion.

**Figure 3:**
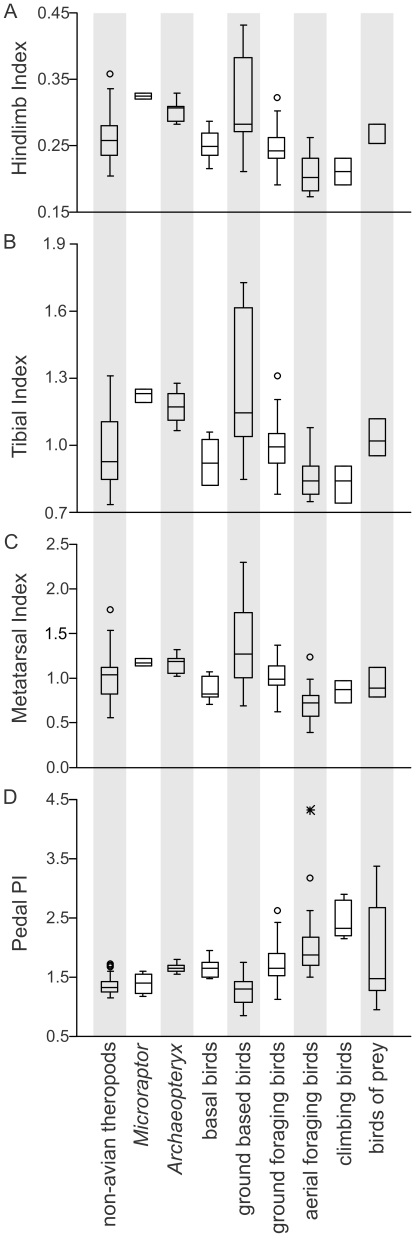
Box-plots for four major bird-specific hindlimb indices. Data are plotted for (A) hindlimb, (B) tibial, (C) tarsometarsus, and (D) pedal phalangeal indices for non-avian theropods and basal and extant birds. To reduce allometric influences, only non-avian theropod taxa less than 111 kg (mass of the largest bird in the sample) are plotted for all indexes except PPI. Outliers 1.5 times the standard deviation above or below the box are denoted by a circle, those 3 times by a star. Note the only taxon more than three times is the Chimney Swift for PPI. Note that *Microraptor* and *Archaeopteryx* are within the range of ground based and ground foraging birds. [planned for single column width].

The bird dataset indicates that total leg length was significantly longer in GB birds, with C and A birds having the lowest scores. Within theropods, *Microraptor*, *Anchiornis* and *Archaeopteryx* show scores more similar to GB birds than any other avian category (Figure 3A). The purported “arboreal” theropods have values greater than 120% the non-avian theropod average, comparable to other small terrestrial theropods, and much larger than basal avians.

#### Brachial index (BI)

Increased levels of arboreality are associated with decreased BI values [25], [42] i.e. relatively longer zeugopodial elements in the forelimb. Microraptorines have significantly larger BI values compared to non-avian theropods (unequal variance t-test t = −4.4725, P = (same)<0.0001) (Figure 3A). Theropods increase their BI values along the lineage toward Aves, most notably within maniraptorans (Table S8, S14). Within Theropoda, there is an allometric scaling component to this signal, although the preponderance of reduced forelimbs in some of the largest taxa (tyrannosaurids and abelisaurids) could be influencing this.

#### Crural index (CI)

CI elongation, i.e. relatively longer zeugopodial elements of the hindlimb, often corresponds to increased cursoriality or leaping in extant clades [69], [70]. Microraptorines have significantly increased CI values compared to other non-avian theropods (unequal variance t-test = −6.2351, P<0.0001). Within theropods, CI is strongly correlated to femur length (Figure S12). The low CI of *Epidendrosaurus* may be due to its hatchling age because crural ratios increase during ontogeny in extant avians [71], [72] and other theropods [73]. It should be noted that the sister taxon to *Epidendrosaurus*, *Epidexipteryx* (CI = 1.24), a nearly mature individual [26], does not show a similar reduction in CI. There is a general increase in CI throughout the evolution of non-avian theropods toward Aves (Table S9, S14), although this may be linked to decreased body sizes in advanced maniraptorans.

Ground based birds showed the highest tibial and tarsometatarsal index values, significantly higher than either climbing or arboreal feeders (Figure 3A, B). Similarly, *Microraptor*, *Anchiornis* and *Archaeopteryx* have high levels of relative elongation in these bones, larger than similar sized non-avian theropods, with levels of metatarsal elongation similar to cursorial taxa such as tyrannosaurs, ornithomimids, and *Caudipteryx*. Basal birds more advanced than *Archaeopteryx* have reduced tibial and tarsometatarsal index values compared to non-avian theropods and *Archaeopteryx*.

#### Manual phalangeal index (MPI)

Claw and grip-based climbing taxa tend to have enlarged MPI values [34], [74]. Microraptorines have significantly lower indices of approximately 1.0 (t = 8.7188, P<0.0001) compared to a mean non-avian theropod value of 1.45. The ratio of the penultimate to proximal phalanx lengths gives a similar result, with microraptorines having significantly smaller values (unequal. var. t = 2.7862, P = 0.017) and *Archaeopteryx* not significantly different than the non-avian theropod mean (unequal. var. t = 0.081254, P = 0.94). There is no significant correlation between metacarpal length and manual MPI in theropods (r = 0.16242, n = 69 P(uncorrelated) = 0.18241). Phylogenetic reconstructions of the evolution of this value toward birds indicated the general gripping manus present in non-avian basal coelurosaurs is not enhanced in purported “arboreal” taxa; in fact, microraptorine dromaeosaurs and the earliest birds show a trend of reduced grasping ability relative to other theropods (Table S12, S14). Although these results are based on the central digital ray, as in previous analyses [32], using digit III, which in all maniraptorans is extremely gracile compared to digit II, yielded similar results. *Microraptor* has the second lowest MPI of all non-avian theropods, and it's penultimate to other phalanges ratio is the lowest of any deinonychosaurs (Table S12). *Archaeopteryx* had a similarly low MPI, but a relatively high penultimate phalangeal ratio, that is comparable to the cursorial ornithomimids. These values for putative arboreal non-avian theropods are directly contrary to what is expected for a grip- or claw-based climber, which have enlarged MPIs to aid in branch grasping.

#### Pedal phalangeal index (PPI)

The PPI shows a pattern similar to the manus. Although *Anchiornis* ( = 1.67) and *Archaeopteryx* ( = 1.54–1.79) are well above the non-avian theropod average (1.44), the majority of microraptorine specimens are below, with only one exception (Figure 3D, Table S13). The values of *Anchiornis* and *Archaeopteryx* are not exceptional with values similar to unquestioned terrestrial taxa, such as *Coelophysis* ( = 1.58), *Huaxignathus* ( = 1.70) and *Procompsognathus* ( = 1.59). Microraptorines have PPI values that are identical to terrestrial non-avian theropods (mean = 1.36, unequal variance t-test, t = −0.05196, P = 0.96) but significantly smaller than basal avians (t = 4.3525, P = 0.002). Unlike in the manus, PPI has a positive correlation with pes length (r = −0.537, P(_uncorrelated_)<0.001, n = 57) and *Epidendrosaurus* has a slightly smaller than expected phalangeal index.

Within extant avians, there is a clear separation between terrestrial and arboreal taxa [63] with arboreal taxa and climbing birds having significantly larger PPI values than either ground based or ground foragers (un-equal variance t-test A/GB = 9.7524, C/Gb = 10.623, G/C = 6.615, P<0.0001, G/A = 3.8172, P = 0.0003). Comparison of the purported “arboreal” theropods to extant birds shows that theropods have a PPI below that expected for arboreal taxa, even after accounting for allometry. Putative “arboreal” non-avian paravians have PI values similar to those expected for terrestrial avians of similar body size (Figure 3D).

### Qualitative analyses

#### Forearm

Although the shoulder joint of non-avian paravians has a laterally facing glenoid, this articulation still restricted the humerus to motions beneath the horizontal plane, unlike the full dorsal extension possible in extant birds for flapping flight [4], [11], [75] and arboreal mammals [76]. Pronation and supination of the forelimb gives greater freedom of movement and allows animals to grip and manipulate small objects. Although the ability to freely pronate and supinate is not restricted to arboreal taxa, its presence allows climbers to utilize branches regardless of their orientation [36], [76]. No theropods can freely pronate or supinate their forelimb, because of the absence of a “radial notch” in the distal contact surface of the ulna and a circular “roller” shape for the distal radius [39], [77]). These morphologies restrict the manus to a medially facing neutral position [77]. Within advanced maniraptorans, there is a degree of pronation-supination between the carpals and the radius [39], yet this is not “active” because this motion is associated with wrist extension [39], [77]. These restrictive movements of the shoulder, elbow, and wrist would limit the climbing ability of non-avian theropods.

#### Hindlimb

Femoral abductive abilities are critical for all arboreal, limbed tetrapods because of the requirements of a large range of motion to select and secure holds, and to reduce the distance between the centre of mass and climbing substrate on small diameter or non-horizontal surfaces [75]. The dinosaurian acetabulum is restricted to an erect stance, verses the semi-sprawling quadrupedal gait of basal archosaurs, with a well-defined supra-acetabular crest that would have limited femoral abduction [76], [78]. The maximal range of hip motion in non-avian theropods is estimated to have been similar to that of extant birds, whose soft tissues further restrict abductive motion, such that most femoral mediolateral movement is done via rotation along the bone's long axis [78]. It is important to note that the hips of *Microraptor* are not significantly different from other theropods with regard to osteological inhibitors of leg abduction and estimated size of leg abductor muscle [79], [80] (pers. obs.). In addition, the basal troodontid *Anchiornis huxleyi*, the oldest deinonychosaur [27], [81], has a distinct and well formed supra-acetabular crest [27], [81]: see fig. S4 therein). A similar crest is present on the well preserved ilium of *Microraptor zhaoianus* (Chinese Academy of Geological Sciences, CAGS 20-8-001) [79]) and the well preserved ilium of the London specimen of *Archaeopteryx lithographica* (British Museum of Natural History, NMH 37001) (Figure S11). The presence of this crest would restrict femoral abductive capability preventing the “splayed” posture typically seen in “four-winged” theropod reconstructions. Despite this osteological restriction, we reran our PCO analyses with scores for non-avian paravian theropods with more mobility in the hips (scoring them as 1, similar to the house cat) to account for different interpretations of this crest. This permutation does not significantly alter the results (Figure S6, S7).

#### Claw morphology

Microraptorines and scansoriopterigids do not have pedal claw curvature values within the range of extant climbing birds, but are more similar to ground-based foragers (e.g. pigeon) [18]. This contradicts previous work on these taxa [15], [17], which relied on the claw curvature data from Feduccia [82]. Feduccia's dataset is unreliable, in part because “[b]irds with unusual adaptations - such as raptors, long-legged marsh birds, long-legged birds (for example, seriamas) that roost and nest low in bushes or trees, birds that resemble *Archaeopteryx*, and so forth - were avoided to eliminate as much as possible birds with claws adapted for strange habits or perceived to be generally convergent with those of *Archaeopteryx* for whatever reason.” [82] pg. 790. The exclusion of morphologically convergent taxa eliminates extant behavioural analogues.

Additionally, predatory bird claw geometry is indistinguishable from that of either perchers or climbers [43] and their inclusion would have significantly diminished Feduccia's categorization of *Archaeopteryx* as arboreal. Glen and Bennet's [18] data encompasses a broader range of bird ecomorphologies/behaviours and limit claw morphometrics to only the dorsal arch of the ungual rather than including the variable and rarely preserved ventral arch and joint of the toe pad (that were included by Feduccia [82], see [18] for a discussion). Recently, Manning and colleagues [83] suggested that dromaeosaur claws were capable of climbing based on finite element analysis of a *Velociraptor* manual claw. Besides our reservations of the use of only manual unguals, the extant “climbing” species used for comparison was the Eagle Owl (*Bubo bubo*), a raptorial bird which uses its claws for prey capture, not climbing [43]. Birn-Jeffery and Rayfield [84] compared pedal claws of dromaeosaurs and trunk climbing birds and found no similarities in design or function. Manual claws of theropods are rarely used in analyses of locomotor function because their primary use is assumed to have been prey capture, and resemble the claws of raptorial birds. Highly recurved manual claws are found in a number of large bodied, undoubtedly terrestrial theropods, including, but not limited to, spinosauroids [85], [86], allosauroids [87]. therizinosaurs [88], [89], and oviraptorids [90].

#### Ankle

Mobility in multiple planes in the ankle joint is critical for arboreal locomotion in extant organisms [25], [70], [91]. All theropods have a mesotarsal ankle joint inherited from their ornithodiran ancestors, which consists of a simple, transversely oriented hinge with movement restricted to the anteroposterior plane [92]. This architecture makes an efficient running joint [36] but is highly ineffective for arboreal locomotion because it limits the mediolateral movement required on the complex surfaces of the arboreal canopy and precludes the hind foot rotation required for head-first descent.

### Requirements for arboreal locomotion

Extant quadrupedal climbers and scansors show modifications to minimize the energy expended during ascent, movement on and between branches and, most critically, descent [25], [93]. The ability to reverse the hindlimb and descend head first is a signature trait of arboreal specialists [35], [94]. In descending head first, the animal gains the ability to accurately gauge and modify the speed and location of its descent [25], [94]. Additionally the ability to freely pronate and supinate the forelimb [35], [36] and highly recurved pedal claws to ensure interlocking with the substrate [18], [57] are necessary to secure a purchase in the complex three-dimensional environment of the canopy. Other characters, such as highly mobile joints (especially the hips and shoulder) and reduction of crural and brachial indices, allowing the centre of mass to be brought closer to the substrate [61], [95] [and references therein] evolved in multiple lineages of advanced climbers and scansors [25], [42], [69], [70]. A notable exception are the brachiating primates, whose locomotion is grip-based and unique among arboreal specialists. Paravian taxa also had long manual feathers that would not have permitted trunk hugging [96], as observed in some modern non-arboreal specialists such as bears and viverids [91], [97]. Thus non-avian theropods would have been unable to descend using either head or tail first methods, a necessary function in any pre-gliding non-avian taxa.

All non-avian theropods lack the level of flexibility in the hindlimbs, especially the ankle joint, present in advanced climbers. Without the ability to rotate their ankles or even to slightly invert them, non-avian theropods would not have been able to grip branches with their hindfeet except when standing orthogonal to it. All non-avian theropods, and the first bird *Archaeopteryx*, lack a reversed hallux [20]. A reversed hallux has been argued for the London specimen of *Archaeopteryx*, but this state is the result of disarticulation [21] (pers. obs.). A definitive, reversed hallux first appeared in the basal avian *Sapeornis* [67], is present in many other Cretaceous birds [66], and is crucial for arboreal locomotion in extant birds [19], [25] because they cannot rotate their ankles. In addition, the hallucal ungual is often hypertrophied to maximize the digital distance angle and adductive forces applied by the foot, which combined with reduced hindlimb length, minimize energy expenditures while climbing [95], [98].

The presence of long feathers on the tibia and tarsus of non-avian paravians has been cited as evidence for a four winged gliding origin of flight and an arboreal stage in avian evolution [13], [16]. It has been argued these feathers would have interfered with terrestrial locomotion which would have induced feather damage [9]. This argument fails to account for the fact that even if *Microraptor* was an arboreal animal it would have to move within the branches (either as a biped or a quadruped), thus engendering the same degree of damage as in a terrestrial setting. Thus any argument that the hindlimb feathers would reduce locomotory ability are equally applicable to a terrestrial or an arboreal context. In addition any crouching posture, as seen in theropods at rest [99] or would occur during climbing [100], would be hampered by such long feathers.

Extant avian and mammalian climbers reduce the distance between their centre of gravity and the substrate by crouching [25], [61], [62]. In this position, the metatarsal feathers of non-avian paravians (some over twice the length of the metatarsus [101]) would be in constant contact with the surface and at risk of being damaged and interfering with arboreal locomotion if the metatarsus was held at any angle less than upright. In climbing animals an upright distal limb segment is not seen during climbing as it raises the centre of mass away from the substrate (the vertical trunk or along sub horizontal thin branches) and increasing the effort during climbing and the rotational forces during branch walking which leads to increased likelihood of falling [25]. This would be particularly acute in non-avian theropod like *Microraptor*, given the lack of a reversed hallux to aid in securing a purchase [19], [20]. Conversely, within extant birds, increased running speed is associated with an increasingly upright stance [102], [103]. The most cursorial of ratites exhibit a highly extended metatarsus with the distal segment at low angles only when raised during the stride [104]. This mechanical behaviour suggests that the simplest way to ensure that feather damage and locomotory interference is reduced is, paradoxically, to be highly cursorial, which agrees with both the limb proportions and relative leg lengths seen in basal deinonychosaurs [27], Dececchi and Larsson, unpublished data.

### WAIR

A terrestrial ecology of avian antecedents suggests the flight stroke evolved on the ground. The origin of powered flight is a more difficult question to address (see Introduction). WAIR behaviour has been documented in at least twenty species of extant birds, including both paleognath and neognaths [23], including their non-volant chicks. This behaviour has been presented as a potential evolutionary narrative for the development of the complex biomechanics that underlie the avian flight stroke with a terrestrial based bird antecedent [11]. WAIR alone cannot be invoked to shore up the trees-down hypothesis because it would only allow limited access to low branches (∼5 m [11]) and does not facilitate movement within or between trees. WAIR also requires a full and powerful flight stroke, with wing force estimates in chukars of up to 220% of body mass and induced velocities comparable to flight [105]. Without modifications to aid in-tree mobility (to permit, for example, prey capture) trees could remain no more than an occasional refuge for non-volant paravians. Although WAIR has been recorded in the Tinamou and some neognaths, the origin of crown Aves (Neornithes) is estimated at approximately 86.5 MYA, using fossils [106], and 130 MYA, using molecular dating [107]. These dates are minimally 30 million years after the origin of birds [27]. Advanced ornithurans, which have musculoskeletal morphologies indicative of powerful flight comparable to extant birds [5], [108], [109] occur at least 50 million years before the oldest known neornithine [110], [111]. This gap indicates a fully developed flight stroke is plesiomorphic for neornithines. Because WAIR is a behavioural trait without osteological specializations, the phylogenetic placement of the flight stroke before the divergence of Neornithes makes it impossible to determine if WAIR is ancestral to the avian flight stroke or derived from it. WAIR is a terrestrial based behaviour to aid in steep incline running and potentially an important step in avian evolution. Yet the uncertainty around its optimization along with its inability to aid with movement along thin diameter substrates (i.e. branches) make WAIR in and of itself insufficient to compensate for the lack of arboreal adaptations seen in non-avian theropods.

### Are Tree Kangaroos good analogues for bird antecedents?

Chatterjee and Templin [14], [100] suggested non-avian theropods need not show significant anatomical changes to be arboreal, citing the tree kangaroo (*Dendrolagus*) as an extant analog, because “adaptation[s are] not apparent in the skeletal features of these animals except for the recurved pedal claws” ([100] pg.165). This comparison fails on multiple fronts: first tree kangaroos are bipedal on the ground and quadrupedal in the trees [112]; second, tree kangaroos are highly modified from their terrestrial counterparts [113], [114]; and they are herbivorous with few natural predators [115]. Moreover, tree kangaroos do have apparent skeletal differences from there more terrestrial conterparts. Tree kangaroos have a modified calcaneocuboid joint to accommodate increased mediolateral rotation of the ankle [116]–[118], reduced curial indices (between 80%–55% those of terrestrial kangaroos) and hindlimb lengths [119], and increased forelimb and axial column flexibility, most notably via highly mobile shoulder and wrist joints [120], [121]. Arboreal adaptations of *Dendrolagus* follow similar trends to other arboreal mammals [113], [114], [118], [121] and in this analysis clearly distinguish them from their terrestrial counterparts (Figure 1, 2, S1, S2, S3, S4, S5, S6, S7). The folivorous diet and lack of arboreal predators of *Dendrolagus* might not have supplied adaptive pressures strong enough to evolve the specialized branch-walking or climbing adaptations present in related marsupial predators [35], [117]. Paravian theropods and most basal birds included in our analysis, in addition to being small [51], were active hunters and would thus require a more extensive range of morphological adaptations to inhabit the canopy.

### Summary

Analysis of discrete skeletal characters throughout the body indicates all non-avian theropods examined here have little to no similarity with modern arboreal taxa, regardless if they employ a mammal-like or bird-like locomotion within the branches. Clustering analyses groups all non-avian theropods and *Archaeopteryx* with terrestrial taxa with a large separation between non-avian theropods and even the more cursorial mammalian scansors (Figure 1, 2, S1, S2, S3, S4, S5, S6, S7). This pattern is repeated using bird specific traits with non-avian theropods clustering closest to ground based birds and far from perching or climbing taxa. Analysis of the hindlimb, as well as the brachial, crural, manual and pedal phalangeal indices all demonstrate none of the expected deviations from the general non-avian theropod condition in putatively “arboreal” non-avian theropods (Figure 3). This lack of change presents no evidence that the undisputed terrestrial locomotion of early theropods was modified for scansorial or arboreal modes of locomotion within non-avian paravians. Nearly all limb metrics have opposing evolutionary trends from what is expected in a clade evolving towards a highly arboreal lifestyle. Unlike non-avian theropods and *Archaeopteryx*, other early Mesozoic birds cluster with perching birds and have limb indices and hindfoot adaptations that suggest they were adept at perching in trees [66].

Our results find no anatomical evidence for a scansorial behaviour for non-avian paravians and *Archaeopteryx*. Instead, these taxa group well with highly cursorial mammals and birds, such as dogs and the ostrich, respectively. Non-avian paravians do not even share the scansorial morphologies of even the least scansorial of mammals, such as the housecat. Although housecats do climb, they have little affinity for movement within the canopy and have particular problems with descent [93] (pers. obs.). If we only look to avian taxa, *Microraptor* and *Archaeopteryx* are morphologically between ratites and galliforms, two avian clades that are definitively ground dwelling. Our results indicate that non-avian, paravian theropods and *Archaeopteryx* did not have adaptations for quadrupedal nor bipedal arboreal climbing, branch-walking, nor descent. The classic protoavis caricature of Heilmann [122] or trunk-clinging dromaeosaurs of Chatterjee [100] and Chatterjee and Templin [14] can be dismissed.

The oldest known birds with definitive arboreal adaptations lived approximately125 million years ago [68]. The five Cretaceous birds included here all cluster well with the the morphologies of extant generalist, ground foraging birds, such as corvids. In Early Cretaceous times, the trees were full of arboreal mammals and reptiles [28], [29], [31], [32], [123] and probably offered some resistance to a novel arboreal clade. No non-avian theropod had the morphological adaptations present in these extinct or extant arboreal taxa. We find no support for arboreality as an ecological strategy of theropods and, therefore, no evidence for the trees down scenario for the origin of the flight stroke in birds. Our results suggest that the ecological setting for the origin of traits required for powered flight, such as the flight stroke, flight feathers, and small body size, is terrestrial.

A terrestrial origin of birds may differentiate them from the other two known flying vertebrate clades. Bats had arboreal, gliding antecedents [124] and pterosaurs, whose origins are unknown [125], are suspected to have also been arboreal gliders [8], [126]. These alternative ecological pathways may have influenced the evolution of their aerofoils (feathers versus skin membranes), which has implications for wing kinematics, aerodynamics, and body size constraints [127], [128]. Thus, the differences between how birds and bats fly may be linked to the ecological setting of their evolutionary origins.

## Supporting Information

Figure S1 **Cluster analysis of total data set.** A) Correlation setting (score 0.8403). B) Euclidean setting (0.7918). Colour coding: Black = theropods, Light Blue = arboreal birds, Blue –green = Fossil arboreal taxa, Dark Blue = basal birds, Brown = lizards, Gold = ground birds, Green = arboreal mammals and the chameleon, Grey = Climbing birds, Khaki = ground based birds, Pink = Archaeopteryx, Purple = birds of prey, Red = terrestrial mammals, Yellow = scansorial mammals.
(PDF)

Figure S2 **Cluster analysis of hindlimb and tail characters from total data set.** A) Correlation setting (score 0.8471) B) Euclidean (score 0.824). Colour coding: Black = theropods, Light Blue = arboreal birds, Blue –green = Fossil arboreal taxa, Dark Blue = basal birds, Brown = lizards, Gold = ground birds, Green = arboreal mammals and the chameleon, Grey = Climbing birds, Khaki = ground based birds, Pink = Archaeopteryx, Purple = birds of prey, Red = terrestrial mammals, Yellow = scansorial mammals.
(PDF)

Figure S3 **Cluster analysis of characters from quadrupedal data set.** A) Correlation setting (score 0.7939) B) Euclidean (score 0.8215). Colour coding: Black = theropods, Light blue = Fossil arboreal taxa, Brown = lizards, Green = arboreal mammals and the chameleon, Pink = Archaeopteryx, Red = terrestrial mammals, Yellow = scansorial mammals.
(PDF)

Figure S4 **Cluster analysis of hindlimb and tail characters from quadrupedal data set.** A) Correlation setting (score 0.8116) B) Euclidean (score 0.8317). Colour coding: Black = theropods, Light blue = Fossil arboreal taxa, Brown = lizards, Green = arboreal mammals and the chameleon, Pink = Archaeopteryx, Red = terrestrial mammals, Yellow = scansorial mammals.
(PDF)

Figure S5 **Correlation box plots for PCO 1.** A) all qudrupedial B) qudrupedial hindlimb only and C) Avian only datasets. In A) and B) stars denote the two chameleon data points while fossil climbers are denoted by (‡).
(PDF)

Figure S6 **PCO total data set with paravian hips scored as 1 (moderately flexible).** A) Correlation setting B) Euclidean setting. Colour coding: Black = theropods, Light Blue = arboreal birds, Blue –green = Fossil arboreal taxa, Dark Blue = basal birds, Brown = lizards, Gold = ground birds, Green = arboreal mammals and the chameleon, Grey = Climbing birds, Khaki = ground based birds, Pink = Archaeopteryx, Purple = birds of prey, Red = terrestrial mammals, Yellow = scansorial mammals.
(PDF)

Figure S7 **Cluster analysis of total dataset with paravian hips set at 1 (moderately flexible).** (A) Correlation setting (score 0.8358) B) Euclidean setting (0.7832). Colour coding: Black = theropods, Light Blue = arboreal birds, Blue –green = Fossil arboreal taxa, Dark Blue = basal birds, Brown = lizards, Gold = ground birds, Green = arboreal mammals and the chameleon, Grey = Climbing birds, Khaki = ground based birds, Pink = Archaeopteryx, Purple = birds of prey, Red = terrestrial mammals, Yellow = scansorial mammals.
(PDF)

Figure S8 **Cluster analysis of avian only data set.** A) Correlation setting (score 0.8478) B) Euclidean setting (0.8547). Colour coding: Black = theropods, Light Blue = arboreal birds, Dark Blue = basal birds, Gold = ground birds, Grey = Climbing birds, Khaki = ground based birds, Pink = Archaeopteryx, Purple = birds of prey.
(PDF)

Figure S9 **PCO of quadrupedal data set using only forelimb characters.** A) correlation setting B) Euclidean (score 0.8317). Colour coding: Black = theropods, Light blue = Fossil arboreal taxa, Brown = lizards, Green = arboreal mammals and the chameleon, Pink = Archaeopteryx, Red = terrestrial mammals, Yellow = scansorial mammals.
(PDF)

Figure S10 **Cluster analysis of quadrupedal data set using forelimb only characters.** A) Correlation setting (score 0.7169) B) Euclidean (score 0.7843). Colour coding: Black = theropods, Light blue = Fossil arboreal taxa, Brown = lizards, Green = arboreal mammals and the chameleon, Pink = Archaeopteryx, Red = terrestrial mammals, Yellow = scansorial mammals.
(PDF)

Figure S11 **The right ilium in lateral aspect of the London specimen of** ***Archaeopteryx lithographica*** **(BMNH 37001).** Note the presence of a well developed supra acetabulum crest (sac), contra [24].
(PDF)

Figure S12 **Bivariate plots of metrics used to measure functional morphologies.** “Terrestrial” theropods (filled circles), microrpatorines (x's), *Archaeopteryx* (open squares), avian theropods (crosses), and Scansoriopterygidae (open circles). A) Relative forelimb length (humerus+ulna) to trunk length. Log_FL_ = −0.6642 Log_Trunk_ (+/−0.063783)+2.2913(+/−0.15463), r = −0.76487, P_(uncorrelated)_<0.001, N = 47. B) Relative hindlimb length to trunk length. Log_TL_ = −0.4968 Log_Trunk_ (+/−0.061771)+2.2919(+/−0.15153), r = −0.52284, P_(uncorrelated)_ = 0.001, N = 49. C) BI regressed against humeral length. Log_BI_ = −0.36873 Log_Humerus_ (+/−0.035123)+1.5559(+/−0.071713), r = −0.48769, P_(uncorrelated)_<0.001, N = 86. D) CI regressed against log femoral length. Log_CI_ = −0.34557 Log_Femur_ (+/−0.022899)+1.9191(+/−0.052394), r = −0.74601, P_(uncorrelated)_<0.001, N = 103. E) Manual PI regressed against log metacarpal length. Log_PI_ = −0.7549 Log_Metacarpal_ (+/−0.091001)+0.11887(+/−0.15101), r = −0.16242, P_(uncorrelated)_ = 0.18241, N = 69. F) Pedal PI regressed against log pes length. Log_PI_ = −0.44659 Log_Pes_ (+/−0.049735)+2.2501(+/−0.090887), r = −0.5638, P_(uncorrelated)_<0.001, N = 57.
(PDF)

Figure S13 **Boxplots comparing within non-avian theropods and basal birds under hindlimb, tibial, and metatarsal indices.**
(PDF)

Table S1 **Cluster analysis of total data**. A = Arboreal, A fossil = extinct fossil taxa, An = ankle mobility (0 = anterioposterior only, 1 = moderate movement in all 3 planes, 2 = highly mobile),BB = basal bird, BOP = bird of prey, C = climbing bird, CI = Crural index, C = Claw geometry (0 = straight, 1 = recurved, 2 = highly recurved), Fl = relative forelimb length (Humerus+Ulna/trunk), G = ground forager, GB = ground based, Hal = Hallux orientation (0 = in line with other digits, 1 = divergent, 2 = opposable, 3 = zygodactyl), HFR = Hindfoot reversal (0 = no, 1 = yes), Hip = femoral abduction ability (0 = low, little to no abduction occurs, 1 = moderate, limited abduction ability during locomotion, 2 = highly mobile), HL = relative hindlimb length (femur+tibia/trunk), H/U = Humerus divided by ulna, MPI = Manual phalangeal index(non-ungual phalanges/metacarpal), PPI = Pedal phalangeal index (non-ungual phalanges/metatarsals, in theropods PhIII 2+3/Ph III-1), Pol = Pollex orientation(0 = inline, 1 = capable of securing item with a “scissor grip”, 2 = opposable), Pro = forearm pronation/supination (0 = none, 1 = yes), Scan = Scansorial, SH = Shoulder (humerus-glenoid joint mobility) (0 = limited to anterioposterior movement, 1 = moderate movement in all 3 planes, 2 = highly mobile even cricumduction), ST = Stance (0 = plantigrade, 1 = sub ungaligrade, 2 = digigrade), Terr = Terrestrial, Tail = tail prehensile/support ability (0 = none, 1 = yes). For all extant avians and *Sinornis*, *Confuciusornis* and *Pengornis*, given the horizontal position of the femora CI index was taken as the tarsometatarsus/tibia.
(PDF)

Table S2 **Cluster analysis of bird data.** A = arboreal forager, BB = basal birds, Bop = bird of prey, C = climbing birds, Claw = Claw geometry (0 = straight, 1 = recurved, 2 = highly recurved), G = ground forager, GB = ground based, Hallux (hallux 0 = non reversed, 1 = reversed but raised, 2 = reversed), Leg L = relative leg length (F+T+Tmt/mass∧0.33), PPI = Pedal phalangeal index (Ph II+III/PhI), TF = tail feathers show adaption for weight supporting adaptation (0 = absent, present = 1), TL = relative tibia length, TMTL = relative tarsometatarsal length, Zy = zygodactyls = (0 = absent, 1 = present).
(PDF)

Table S3 **PCO loadings for first 3 axes for total dataset.** Percentage of variance explained by the first three axes for Euclidean setting: 39.0, 23.9 and 10.3%. For Correlation setting: 35.3, 16.7 and 7.4%. All other axes explain less than 5% of the variance.
(PDF)

Table S4 **PCO loadings for first 3 axes for total dataset using hindlimb characters only.** Percentage of variance explained by the first three axes for Euclidean setting: 42.6, 25.3 and 12.6%. For Correlation setting: 40.5, 18.0 and 8.0%. All other axes explain less than 5% of the variance.
(PDF)

Table S5 **PCO loadings for first axes for the quadrupedal only dataset.** Percentage of variance explained by the first four axes for Euclidean setting: 47.2, 16.9, 10.2 and 5.2% For Correlation setting the first three axes explained: 39.0, 16.1 and 4.9%. All other axes explain less than 5% of the variance.
(PDF)

Table S6 **PCO loadings for first 3 axes for the quadrupedal only dataset using hindlimb characters only.** Percentage of variance explained by the first four axes for Euclidean setting: 50.7, 16.7, 10.5 and 6.4% For Correlation setting the first three axes explained: 47.5, 14.9 and 5.3%. All other axes explain less than 5% of the variance.
(PDF)

Table S7 **PCO loadings for first 3 axes for bipedal (avian) only dataset.** Percentage of variance explained by the first three axes for Euclidean setting: 68.0, 14.1 and 7.9%. For Correlation setting: 60.4, 10.5 and 6.7%. All other axes explain less than 5% of the variance.
(PDF)

Table S8 **Forelimb measurements and BI for theropods and basal birds.**
(PDF)

Table S9 **Hindlimb measurements and CI for theropods and basal birds.**
(PDF)

Table S10 **Bird hindlimb lengths and relative length scores.** M = mass, F = femur length, T = tibia length, TMT = tarsometatarsus length, Leg L = leg length (F+T+TMT), Rleg L = (F+T+TMT)/M0.33, TL = T/M0.33, RTL = TL/Avg, TMTL = TMT/M0.33, R TMTL = TMTL/Avg.
(PDF)

Table S11 **Theropod hindlimb lengths and relative length scores.** Est C = mass estimate based on femoral circumference from Christiansen and Farina 2004, Est L = estimate based on femoral length from Christiansen and Farina 2004, M = mass used in kg, F = femur length, T = tibia length, Mt = metatarsus length, Leg L = leg length (F+T+Mt), Rleg L = (F+T+TMT)/M0.33, TL = T/M0.33, RTL = TL/Avg, TMTL = TMT/M0.33, R TMTL = TMTL/Avg.
(PDF)

Table S12 **Manual phalangeal indices of non-avian theropods and early avians.** A) Digit II B) Digit III.
(PDF)

Table S13 **Pedal phalangeal indices of non-avian theropods and early avians.**
(PDF)

Table S14 **Phylogenetic nodal reconstructions across Theropoda into basal birds.**
(PDF)

Text S1 **References cited in all supplementary information.**
(PDF)
