# Supplementary figures and images for: Assessing Arboreal Adaptations of Bird Antecedents: Testing the Ecological Setting of the Origin of the Avian Flight Stroke
Source: PLoS One. 2011 Aug 9;6(8):e22292. doi: 10.1371/journal.pone.0022292 (PMC3153453; doi:10.1371/journal.pone.0022292)

A)

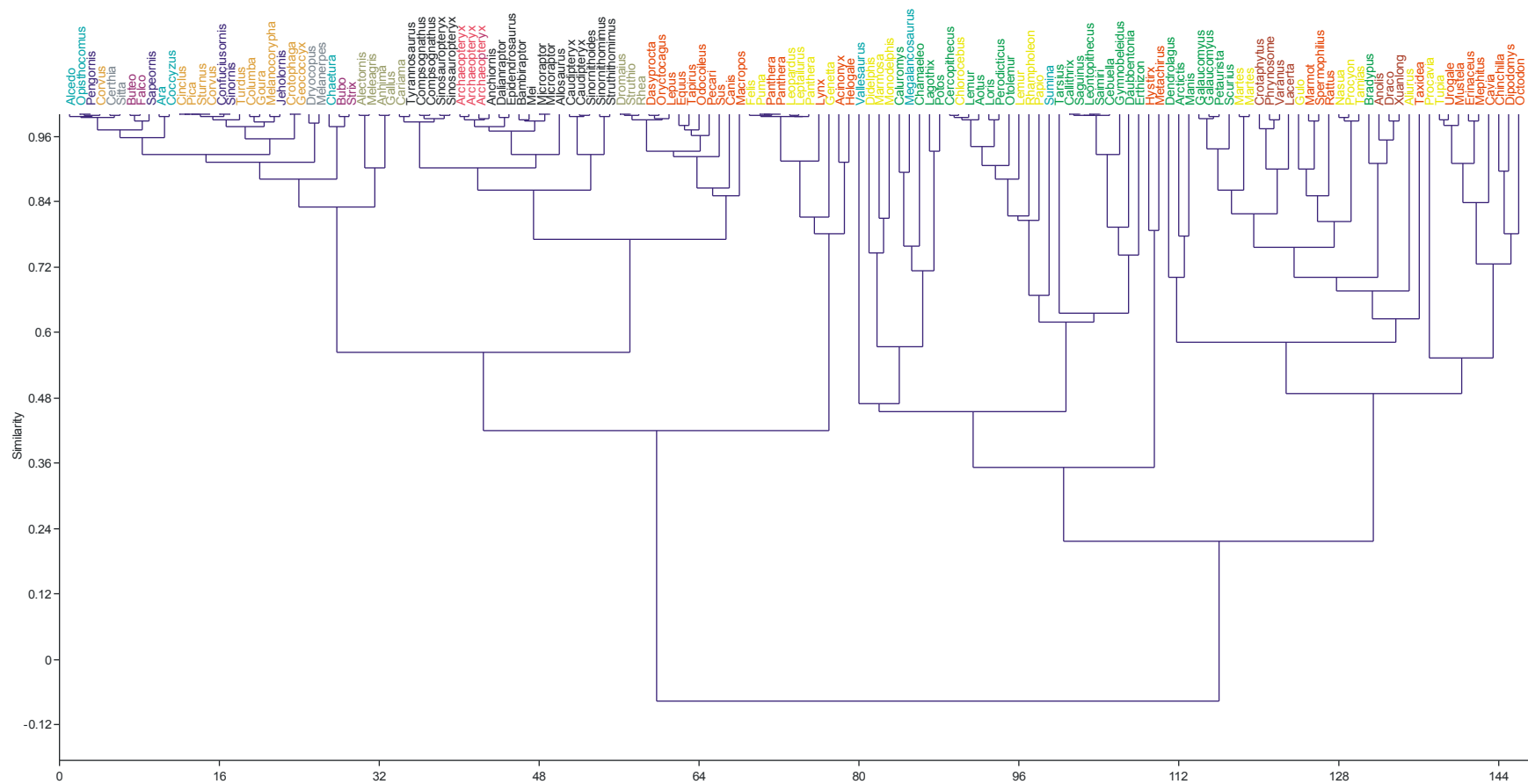

B)

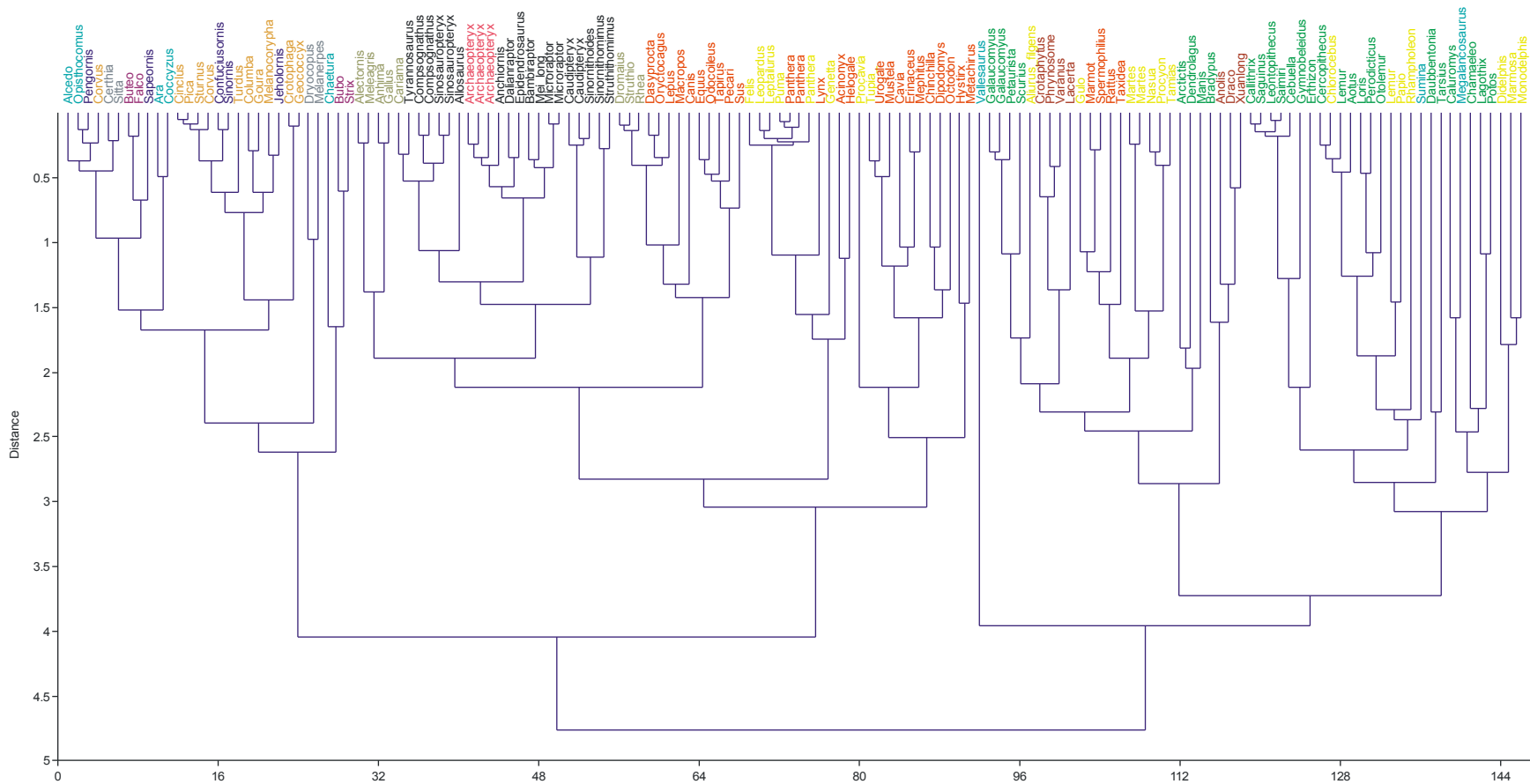

Supplement: Figure S1 — Cluster analysis of total data set. A) Correlation setting (score 0.8403). B) Euclidean setting (0.7918). Colour coding: Black = theropods, Light Blue = arboreal birds, Blue –green = Fossil arboreal taxa, Dark Blue = basal birds, Brown = lizards, Gold = ground birds, Green = arboreal mammals and the chameleon, Grey = Climbing birds, Khaki = ground based birds, Pink = Archaeopteryx, Purple = birds of prey, Red = terrestrial mammals, Yellow = scansorial mammals. (PDF) [file pone.0022292.s001.pdf]

A)

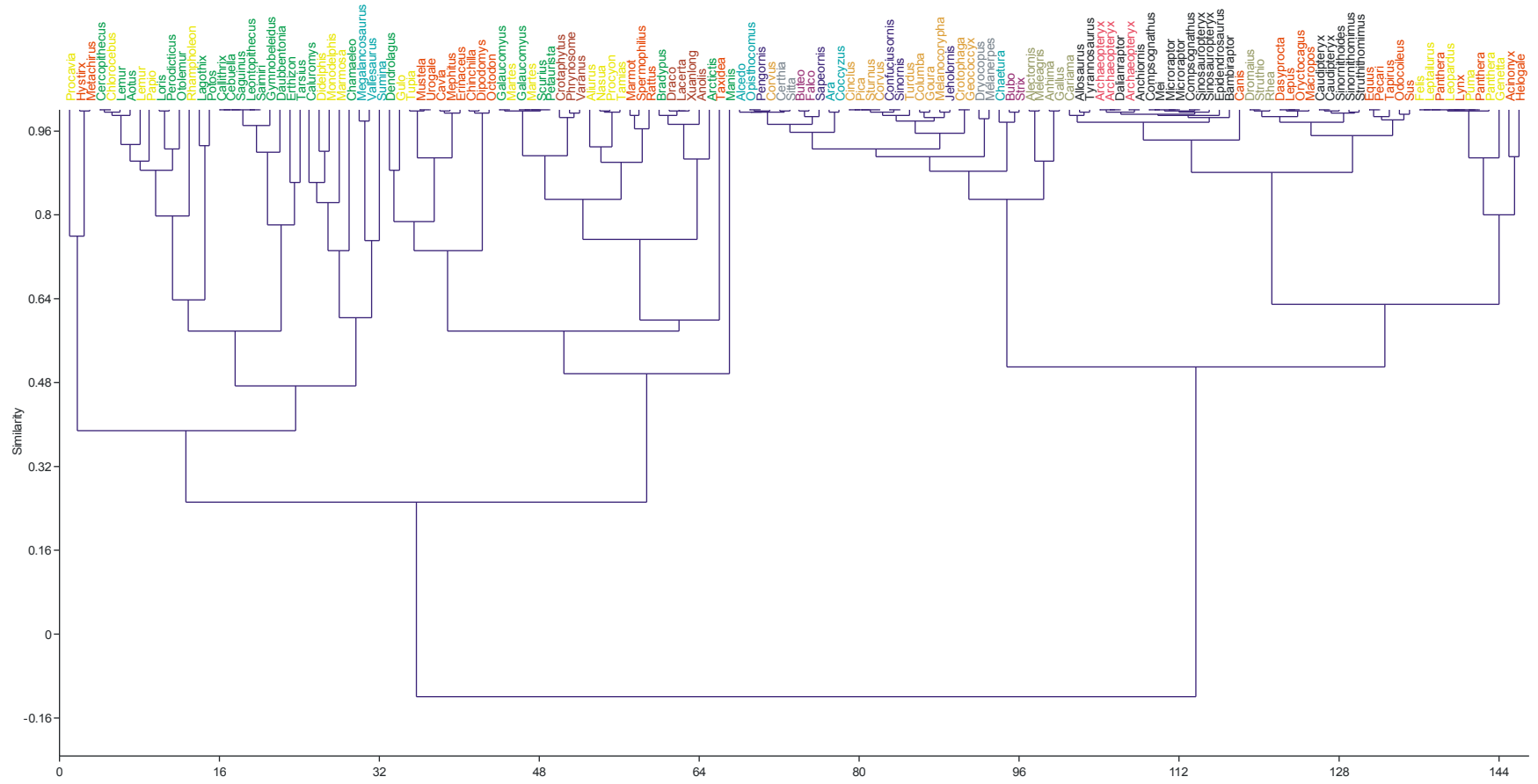

B)

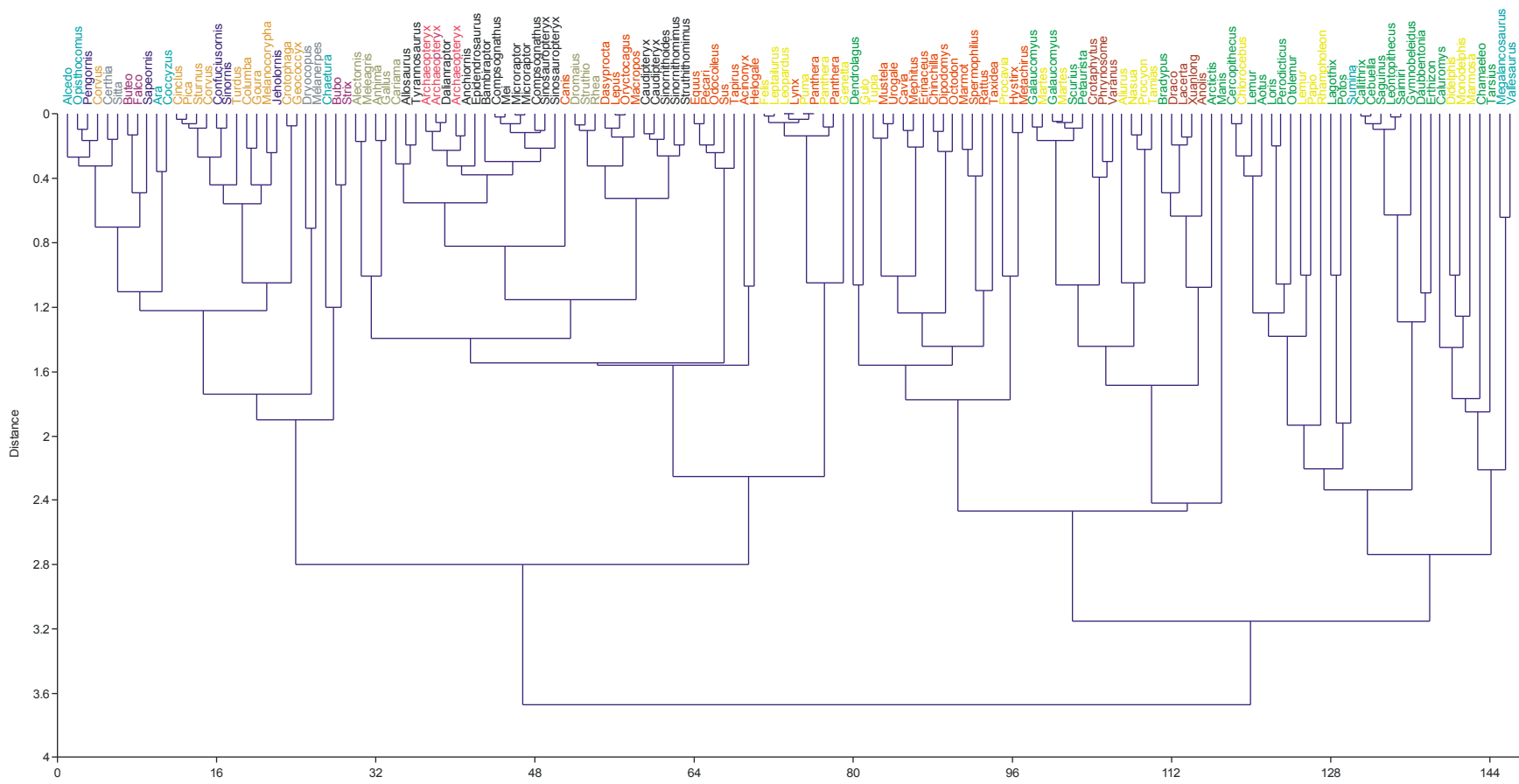

Supplement: Figure S2 — Cluster analysis of hindlimb and tail characters from total data set. A) Correlation setting (score 0.8471) B) Euclidean (score 0.824). Colour coding: Black = theropods, Light Blue = arboreal birds, Blue –green = Fossil arboreal taxa, Dark Blue = basal birds, Brown = lizards, Gold = ground birds, Green = arboreal mammals and the chameleon, Grey = Climbing birds, Khaki = ground based birds, Pink = Archaeopteryx, Purple = birds of prey, Red = terrestrial mammals, Yellow = scansorial mammals. (PDF) [file pone.0022292.s002.pdf]

A)

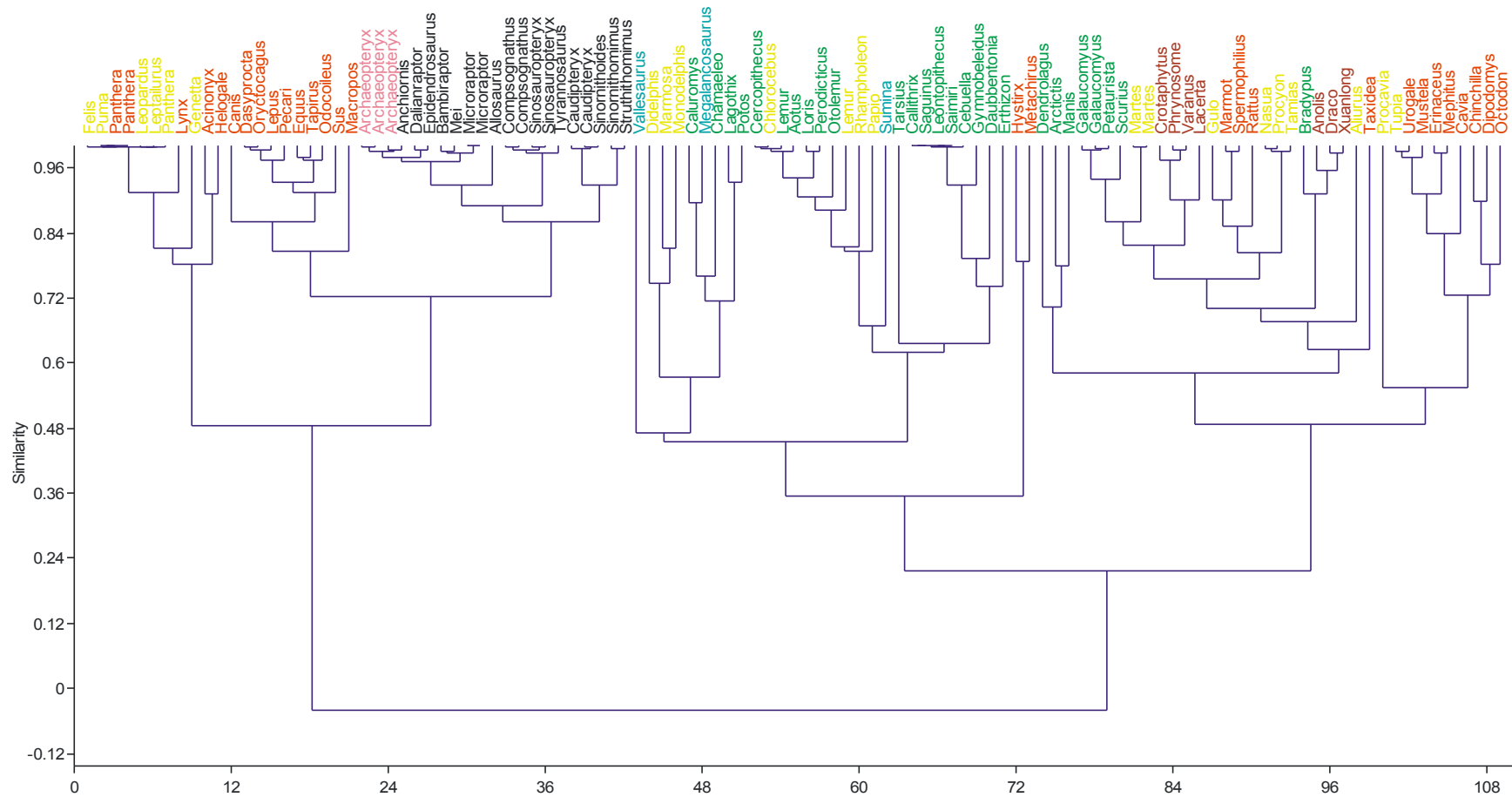

B)

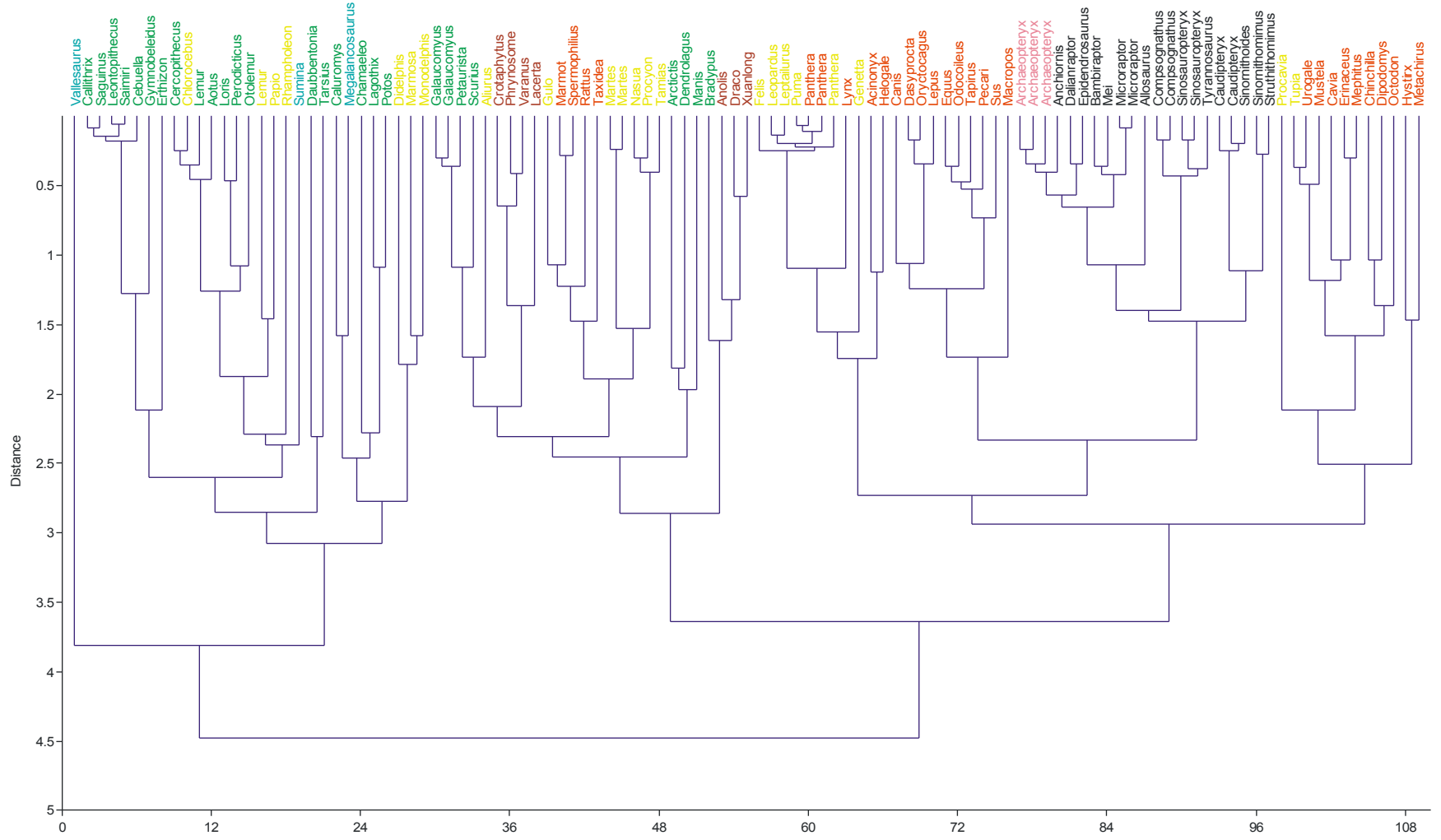

Supplement: Figure S3 — Cluster analysis of characters from quadrupedal data set. A) Correlation setting (score 0.7939) B) Euclidean (score 0.8215). Colour coding: Black = theropods, Light blue = Fossil arboreal taxa, Brown = lizards, Green = arboreal mammals and the chameleon, Pink = Archaeopteryx, Red = terrestrial mammals, Yellow = scansorial mammals. (PDF) [file pone.0022292.s003.pdf]

A)

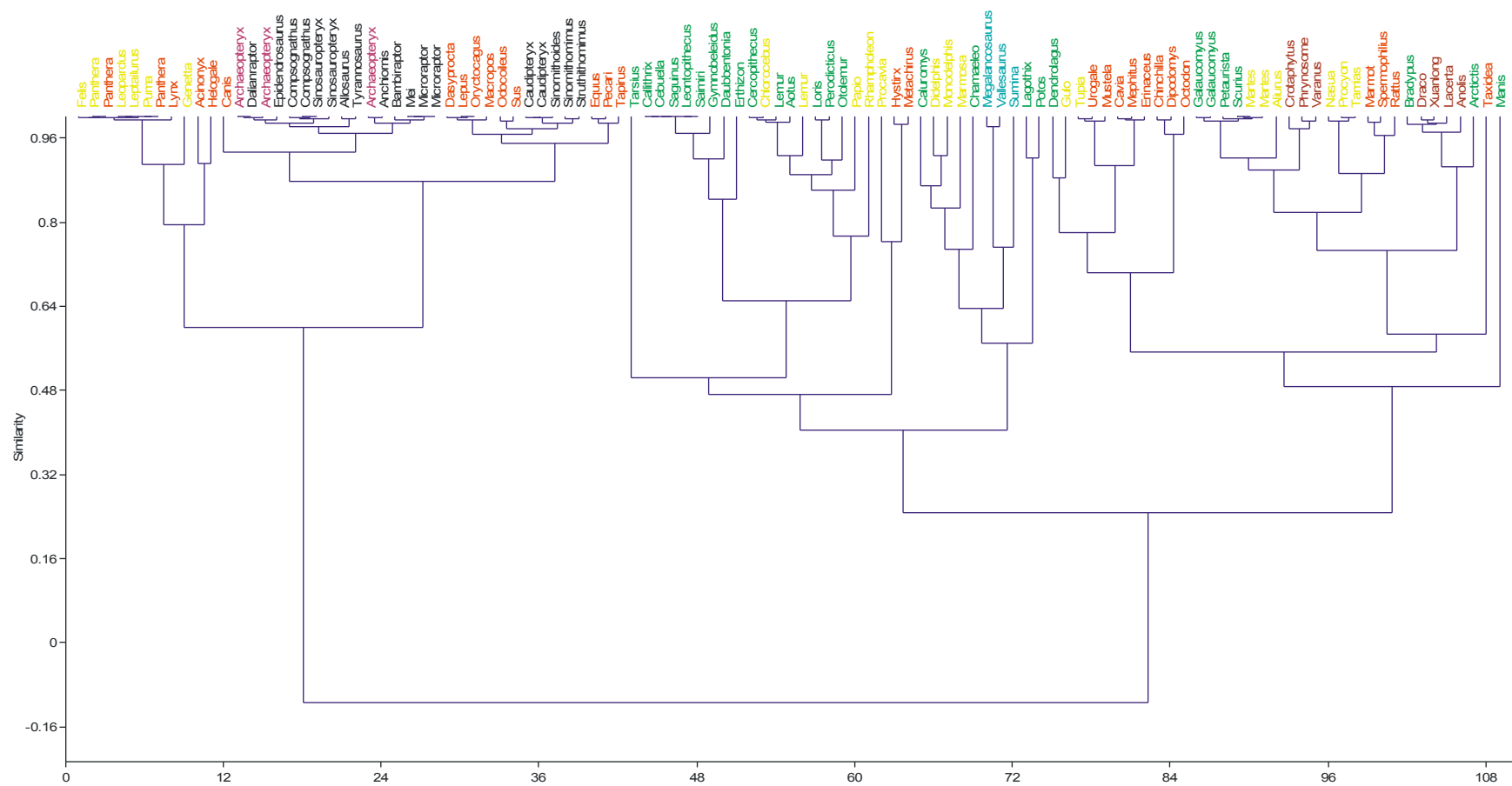

B)

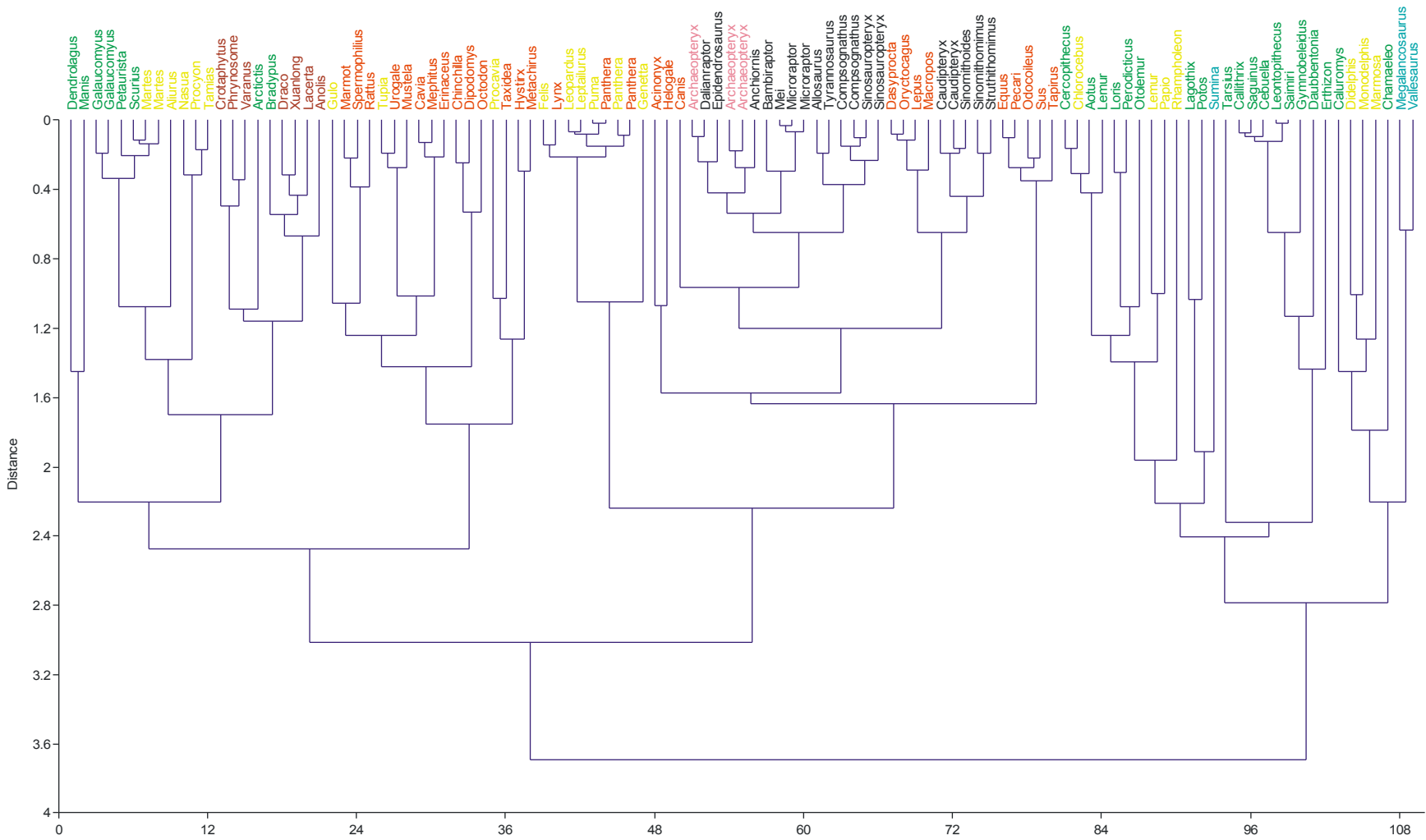

Supplement: Figure S4 — Cluster analysis of hindlimb and tail characters from quadrupedal data set. A) Correlation setting (score 0.8116) B) Euclidean (score 0.8317). Colour coding: Black = theropods, Light blue = Fossil arboreal taxa, Brown = lizards, Green = arboreal mammals and the chameleon, Pink = Archaeopteryx, Red = terrestrial mammals, Yellow = scansorial mammals. (PDF) [file pone.0022292.s004.pdf]

A)

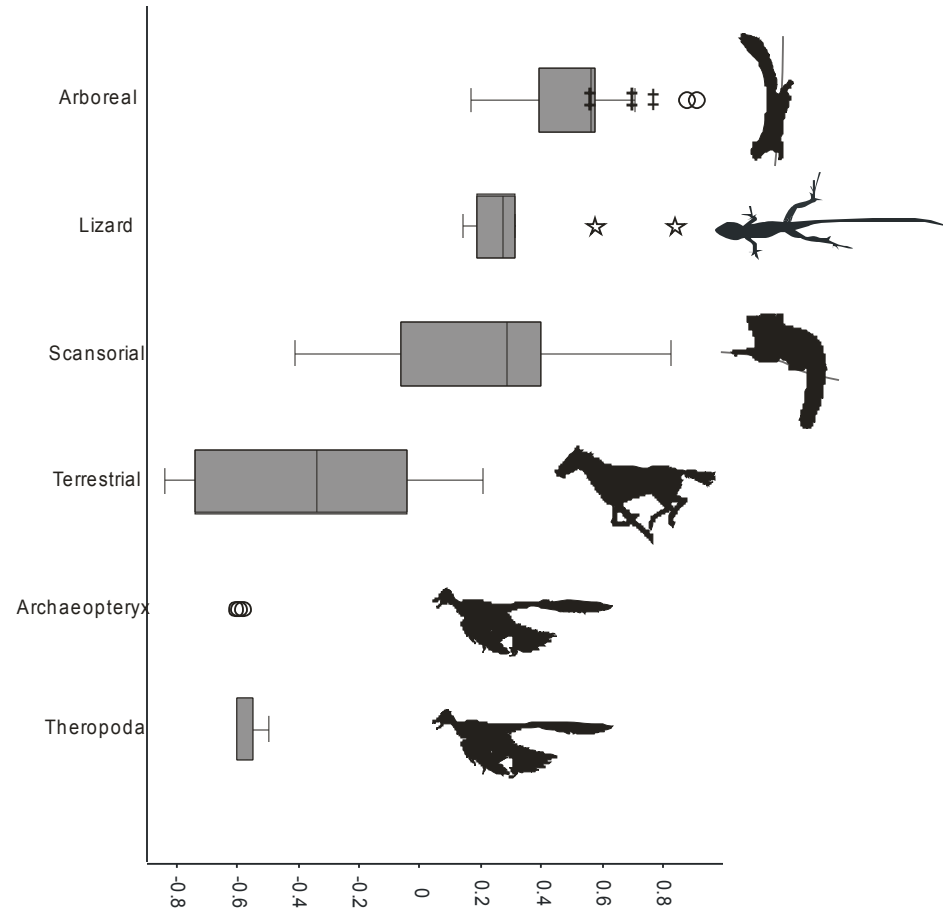

B)

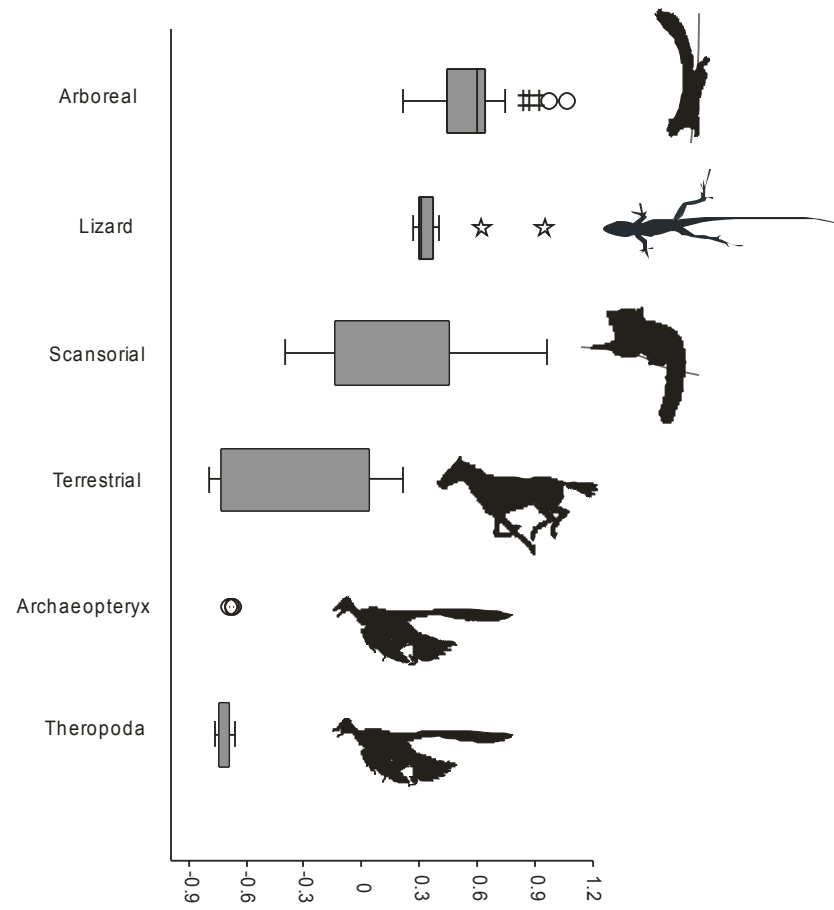

C)

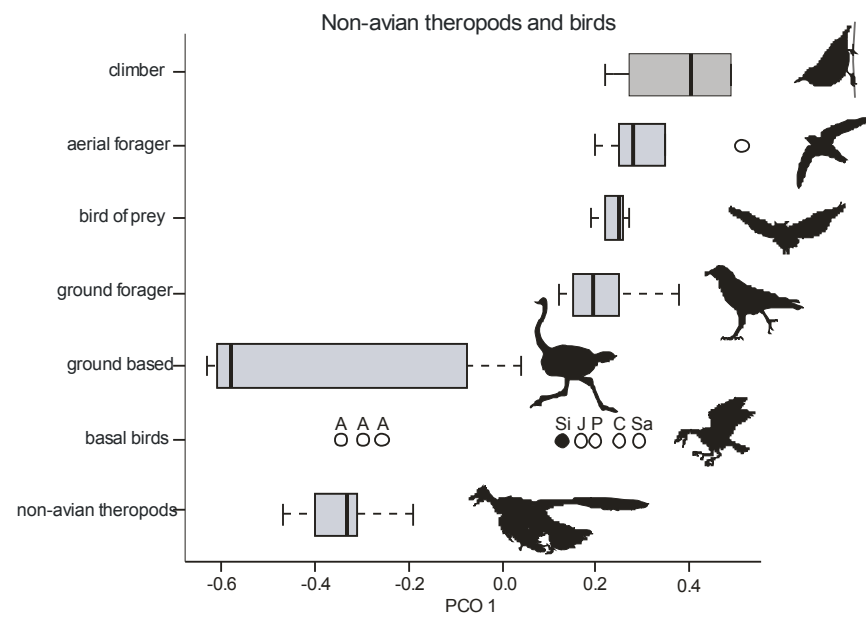

Supplement: Figure S5 — Correlation box plots for PCO 1. A) all qudrupedial B) qudrupedial hindlimb only and C) Avian only datasets. In A) and B) stars denote the two chameleon data points while fossil climbers are denoted by (‡). (PDF) [file pone.0022292.s005.pdf]

A)

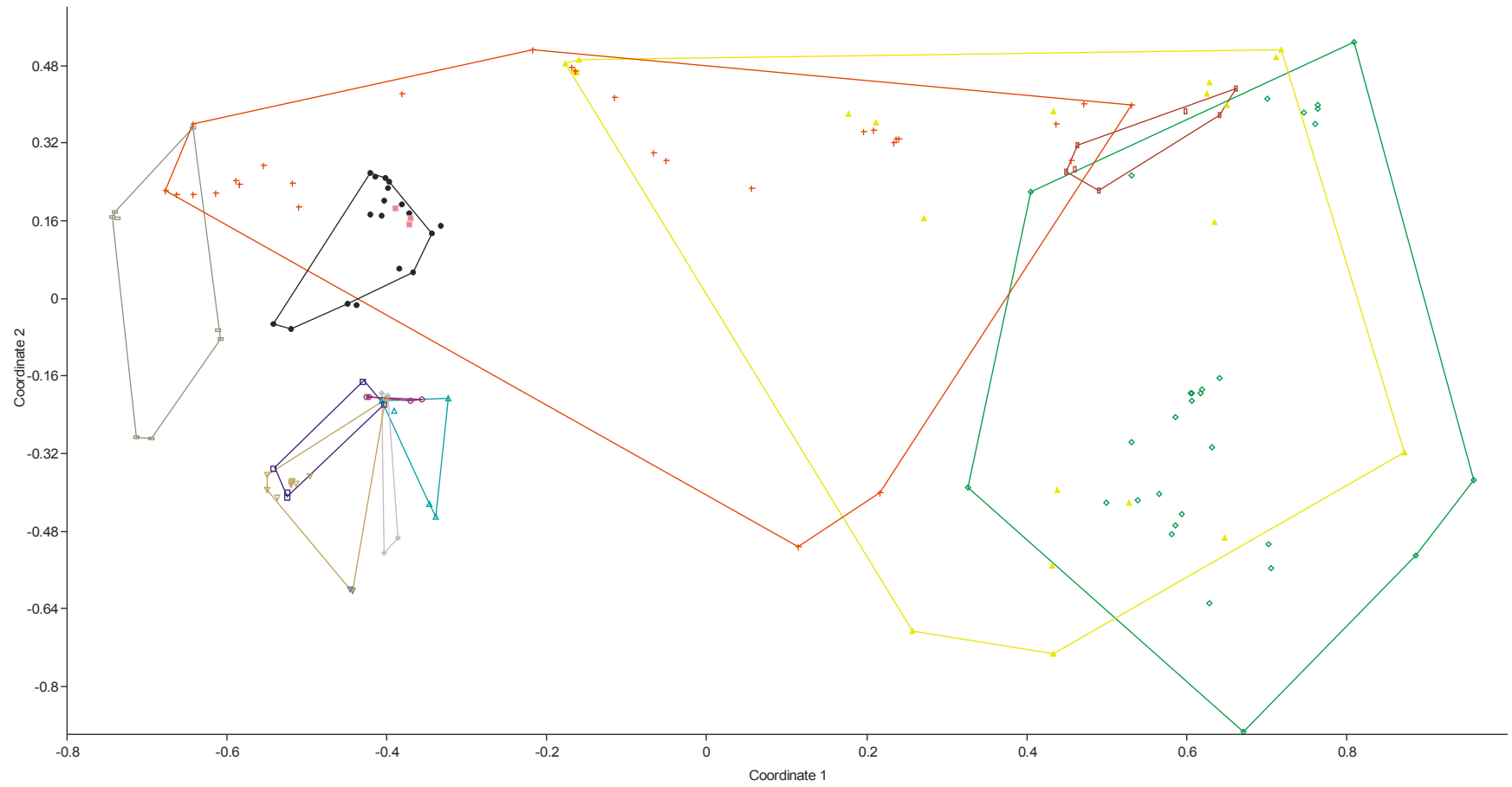

B)

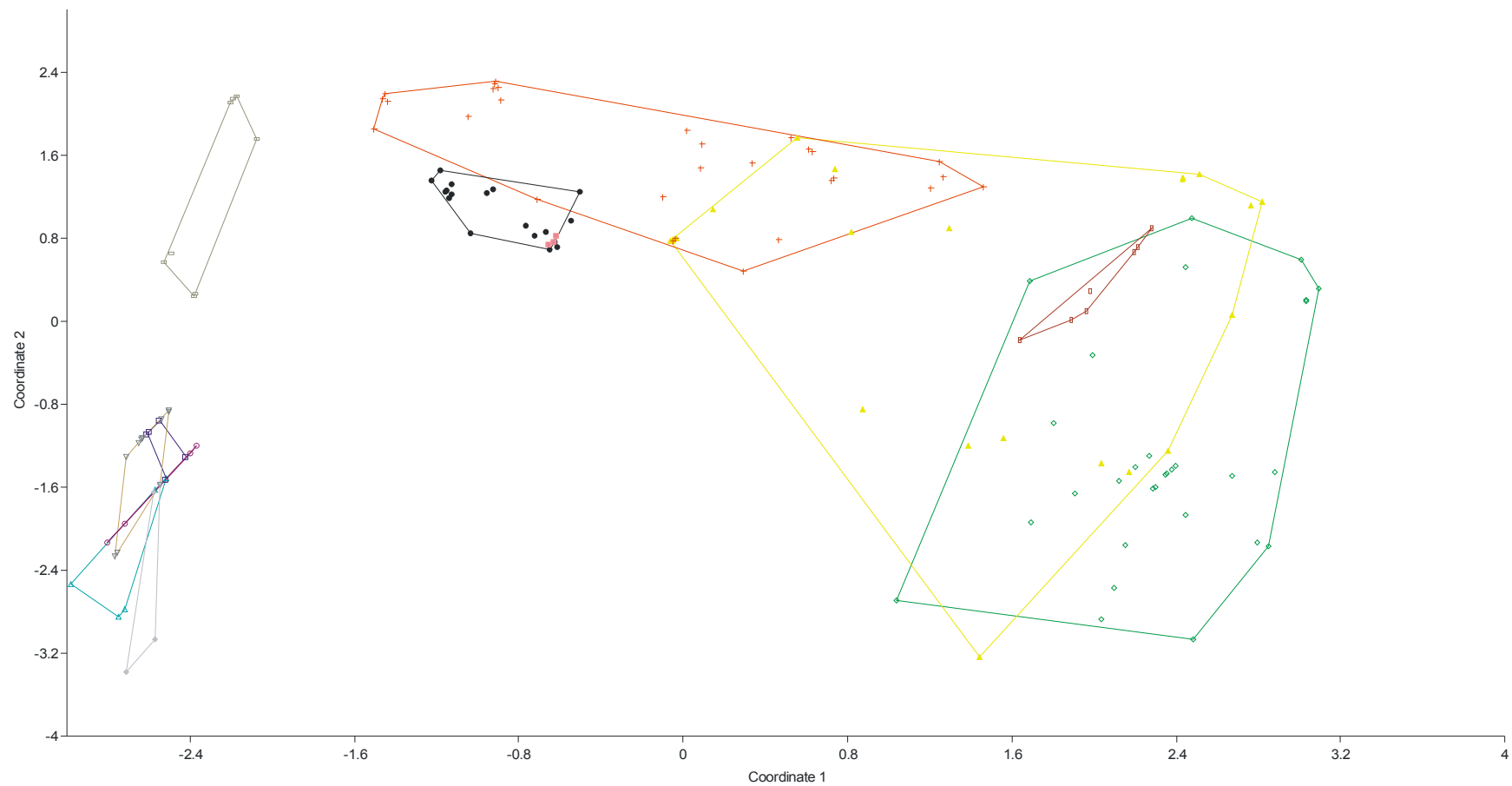

Supplement: Figure S6 — PCO total data set with paravian hips scored as 1 (moderately flexible). A) Correlation setting B) Euclidean setting. Colour coding: Black = theropods, Light Blue = arboreal birds, Blue –green = Fossil arboreal taxa, Dark Blue = basal birds, Brown = lizards, Gold = ground birds, Green = arboreal mammals and the chameleon, Grey = Climbing birds, Khaki = ground based birds, Pink = Archaeopteryx, Purple = birds of prey, Red = terrestrial mammals, Yellow = scansorial mammals. (PDF) [file pone.0022292.s006.pdf]

A)

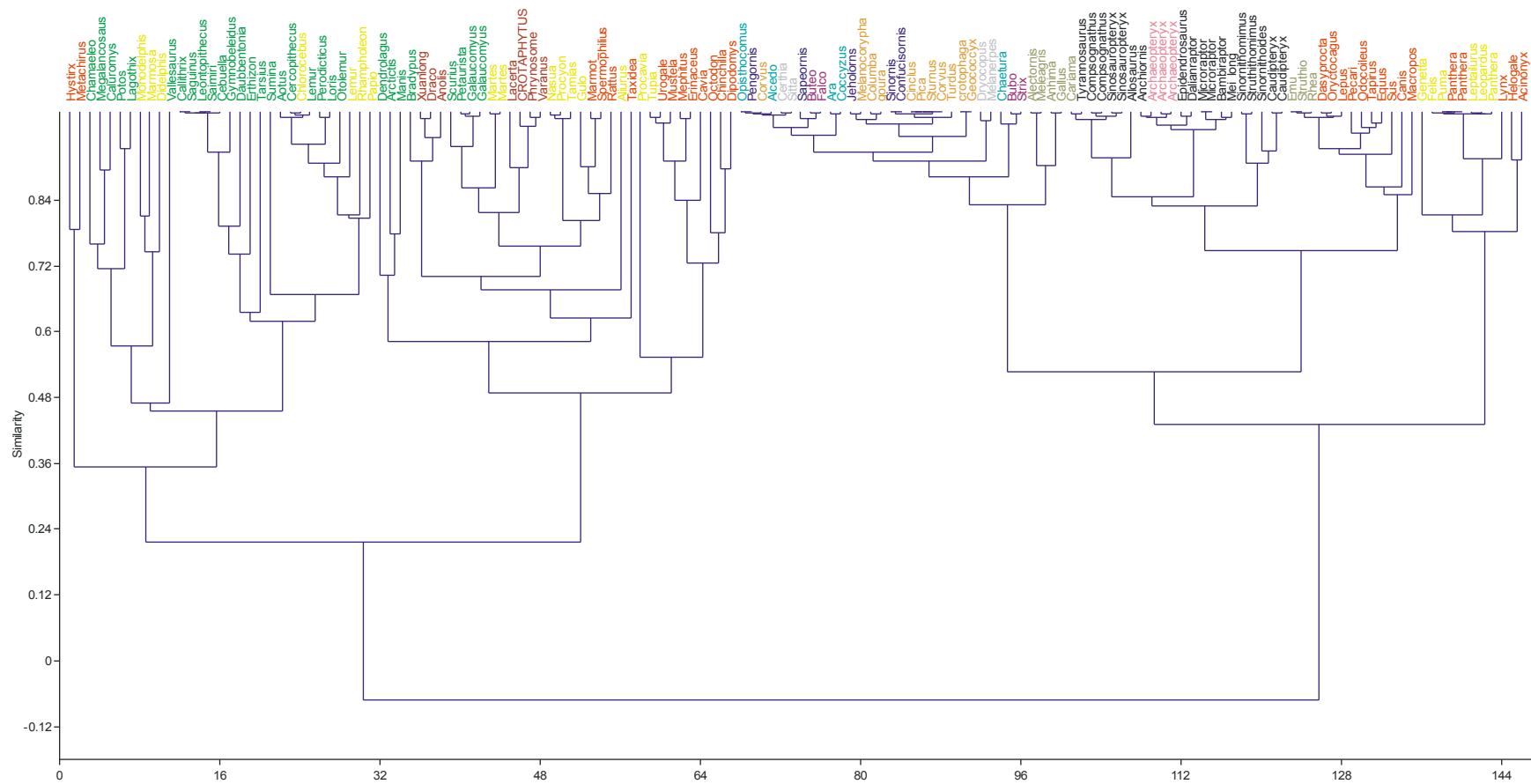

B)

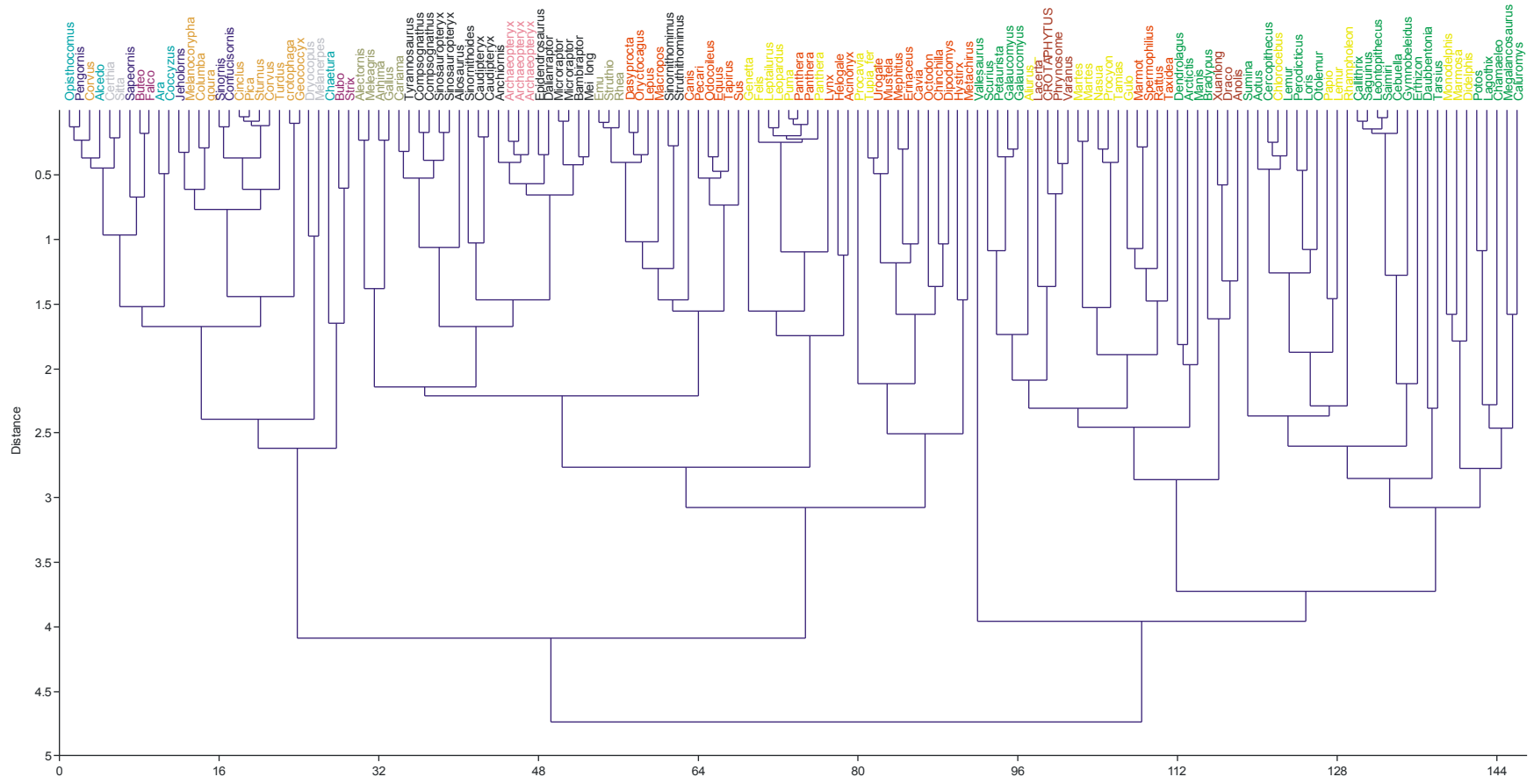

Supplement: Figure S7 — Cluster analysis of total dataset with paravian hips set at 1 (moderately flexible). (A) Correlation setting (score 0.8358) B) Euclidean setting (0.7832). Colour coding: Black = theropods, Light Blue = arboreal birds, Blue –green = Fossil arboreal taxa, Dark Blue = basal birds, Brown = lizards, Gold = ground birds, Green = arboreal mammals and the chameleon, Grey = Climbing birds, Khaki = ground based birds, Pink = Archaeopteryx, Purple = birds of prey, Red = terrestrial mammals, Yellow = scansorial mammals. (PDF) [file pone.0022292.s007.pdf]

A)

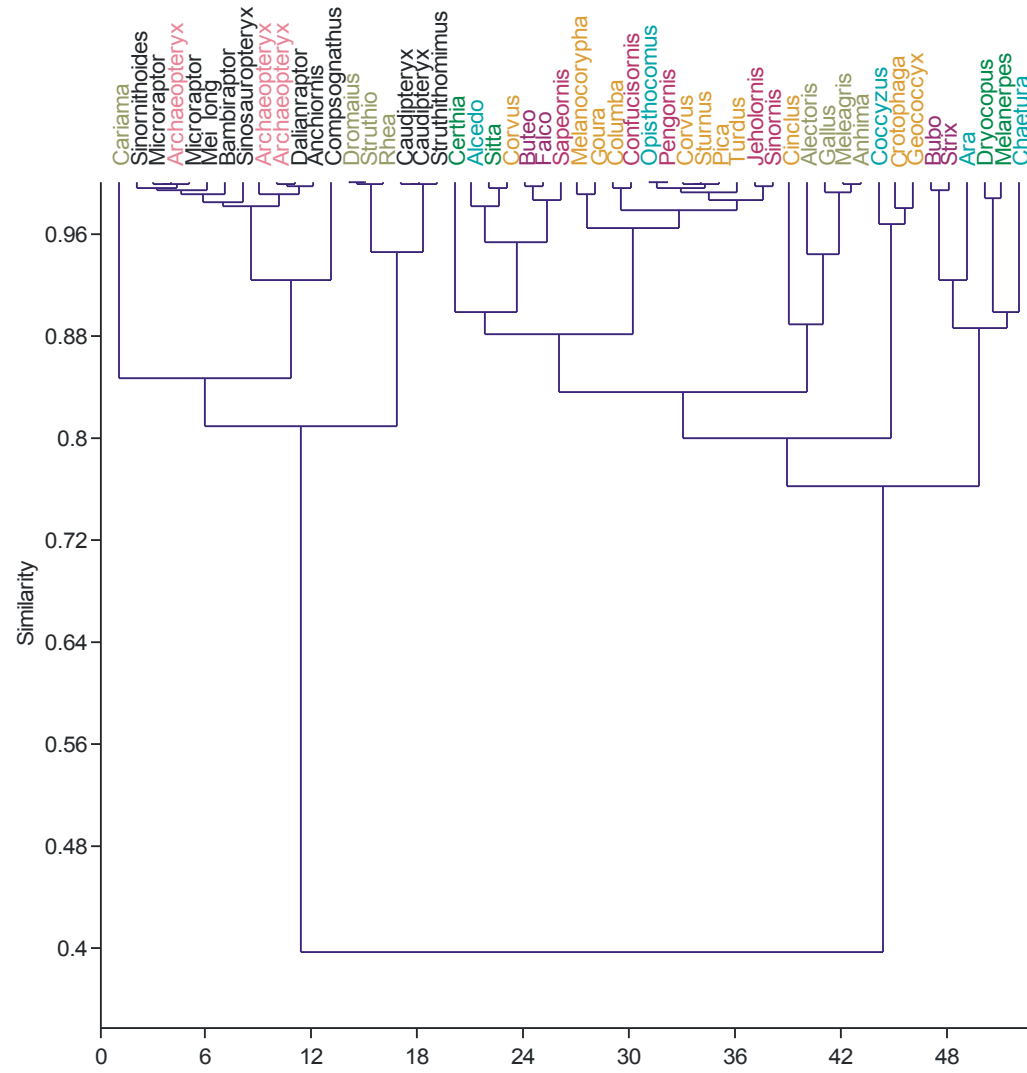

B)

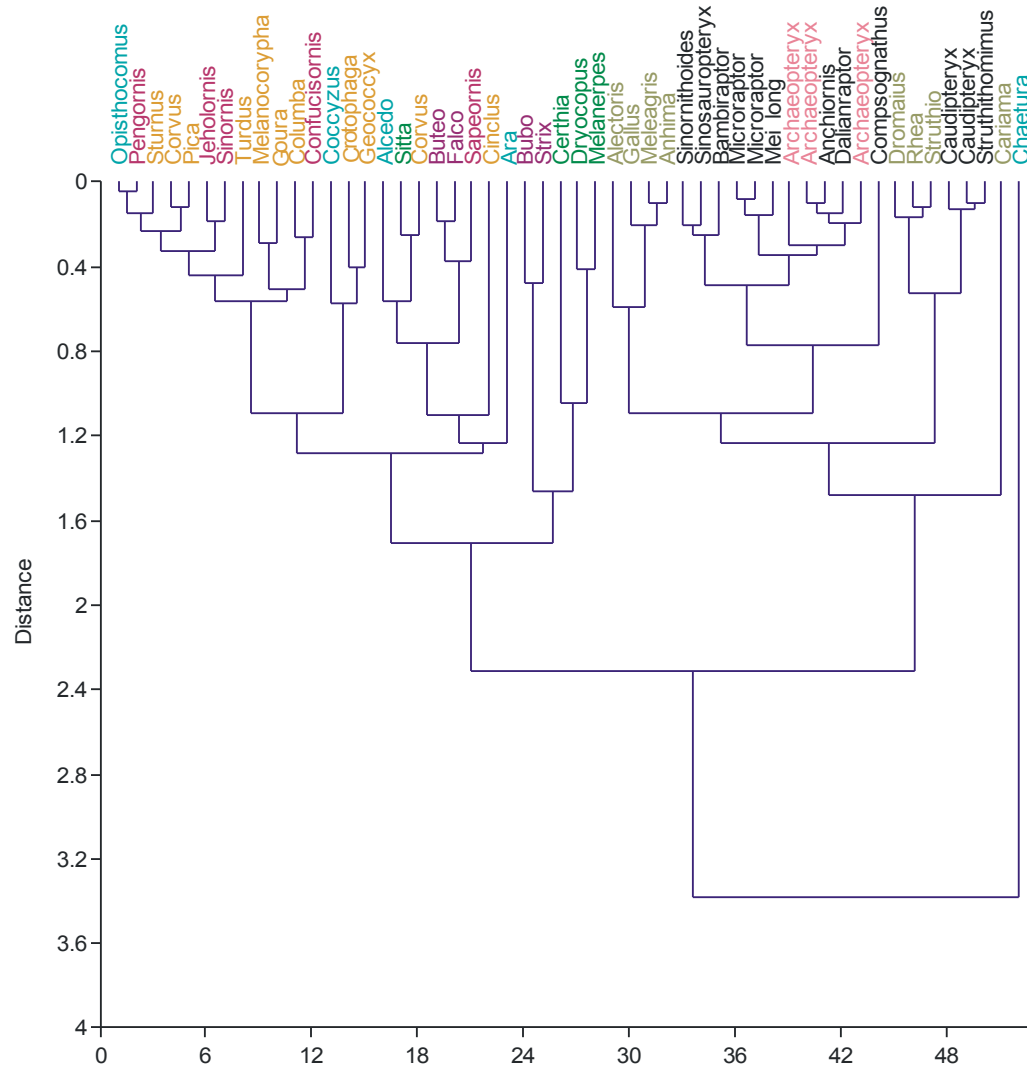

Supplement: Figure S8 — Cluster analysis of avian only data set. A) Correlation setting (score 0.8478) B) Euclidean setting (0.8547). Colour coding: Black = theropods, Light Blue = arboreal birds, Dark Blue = basal birds, Gold = ground birds, Grey = Climbing birds, Khaki = ground based birds, Pink = Archaeopteryx, Purple = birds of prey. (PDF) [file pone.0022292.s008.pdf]

A)

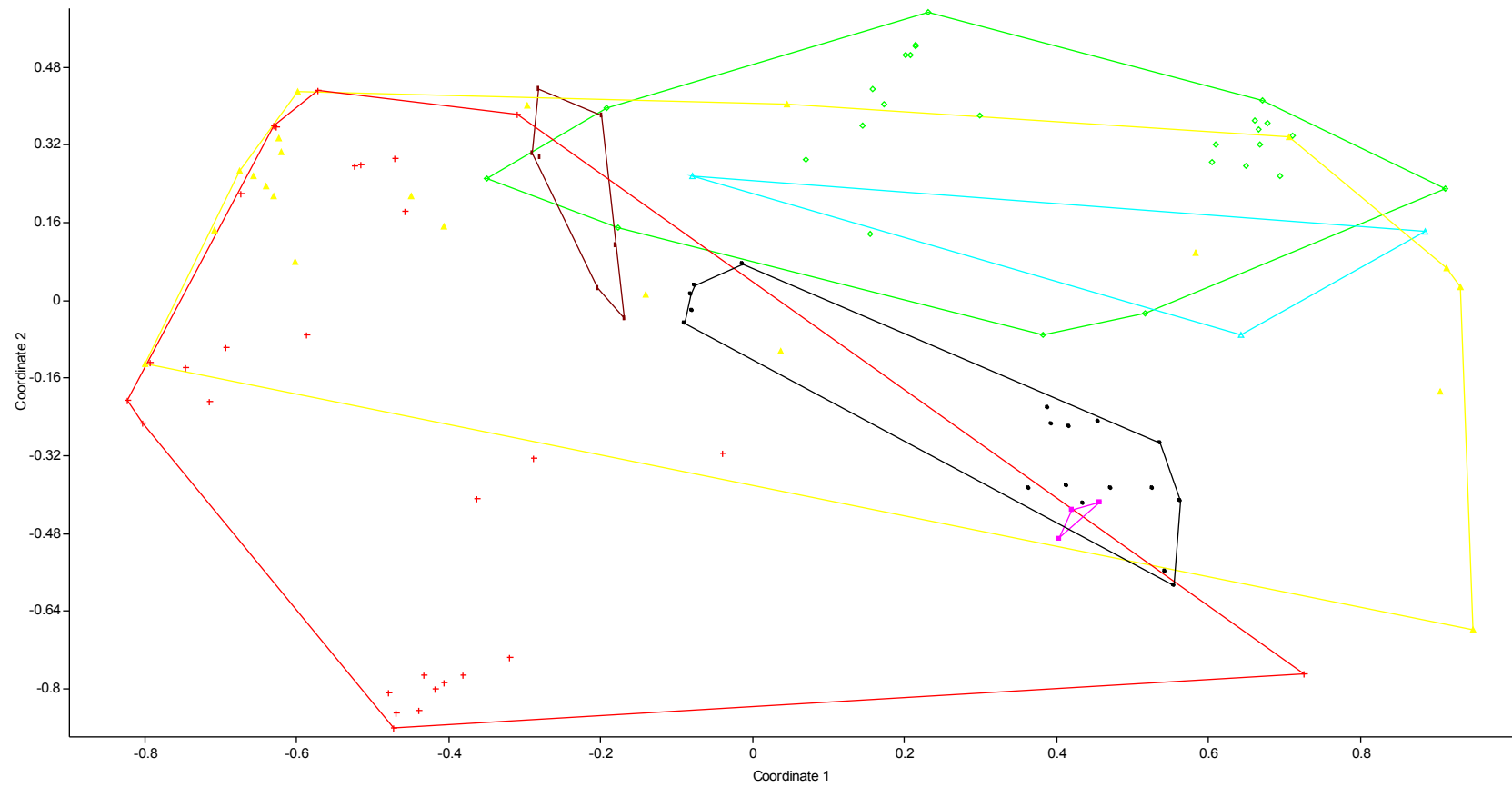

B)

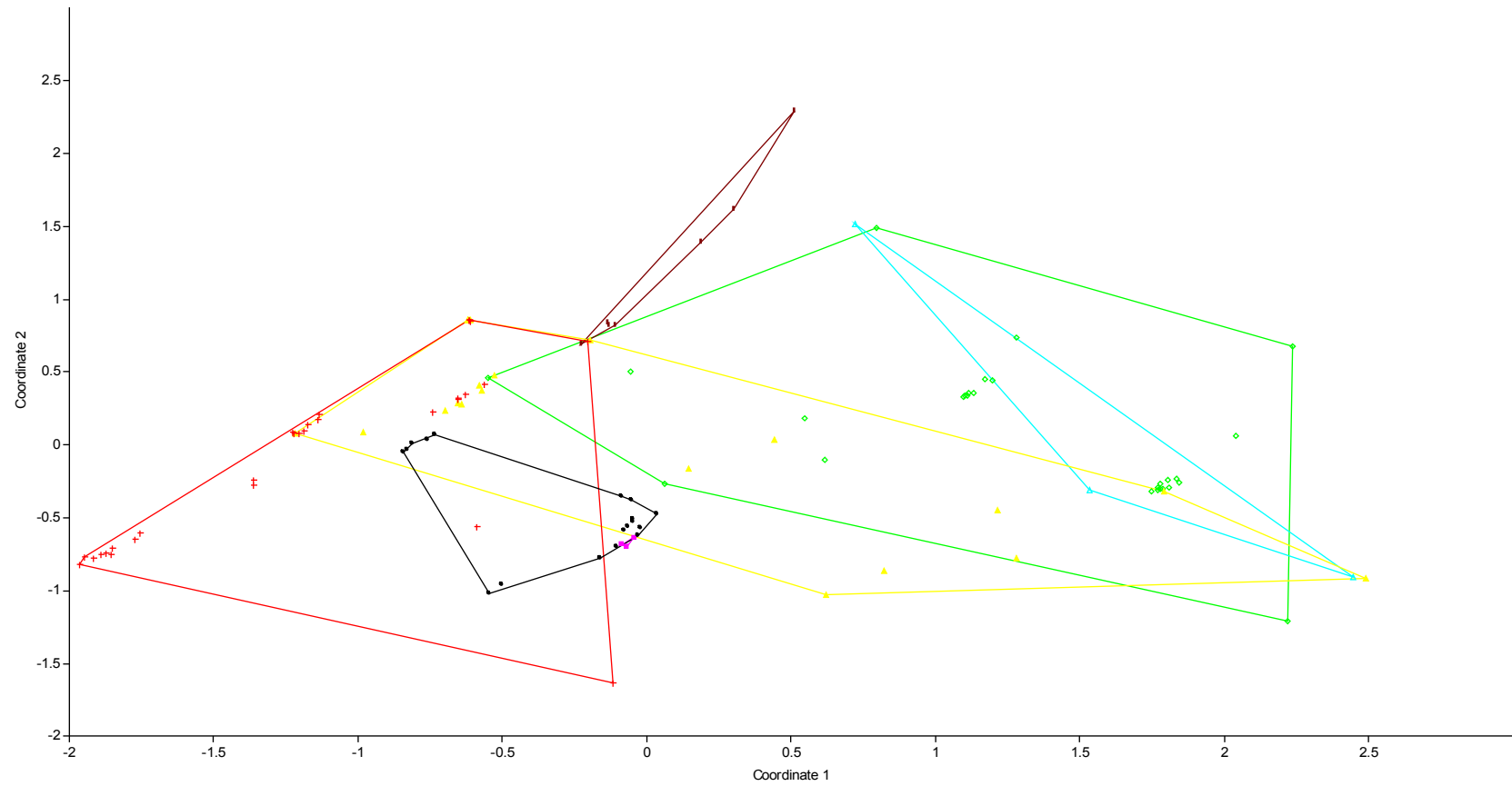

Supplement: Figure S9 — PCO of quadrupedal data set using only forelimb characters. A) correlation setting B) Euclidean (score 0.8317). Colour coding: Black = theropods, Light blue = Fossil arboreal taxa, Brown = lizards, Green = arboreal mammals and the chameleon, Pink = Archaeopteryx, Red = terrestrial mammals, Yellow = scansorial mammals. (PDF) [file pone.0022292.s009.pdf]

A)

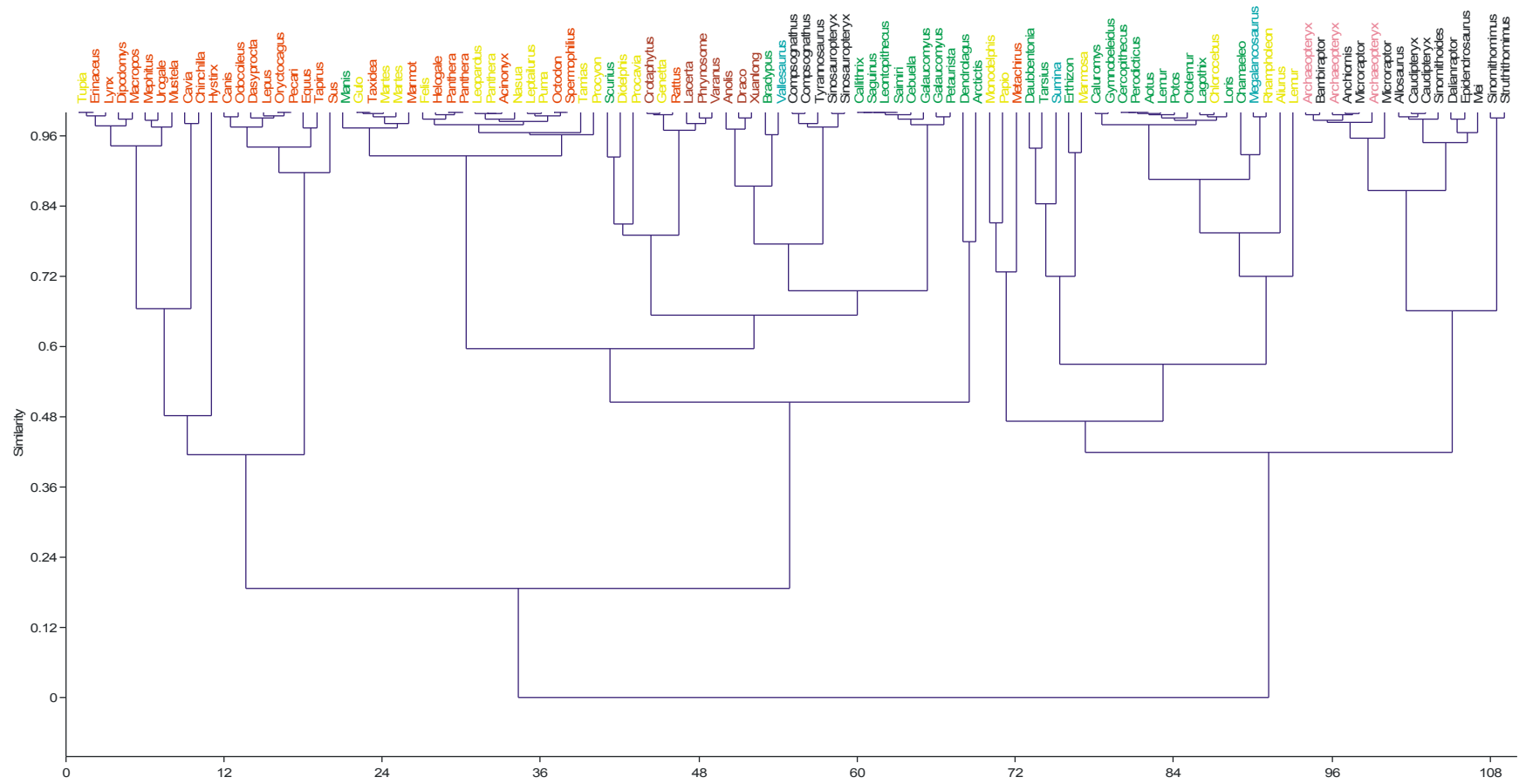

B)

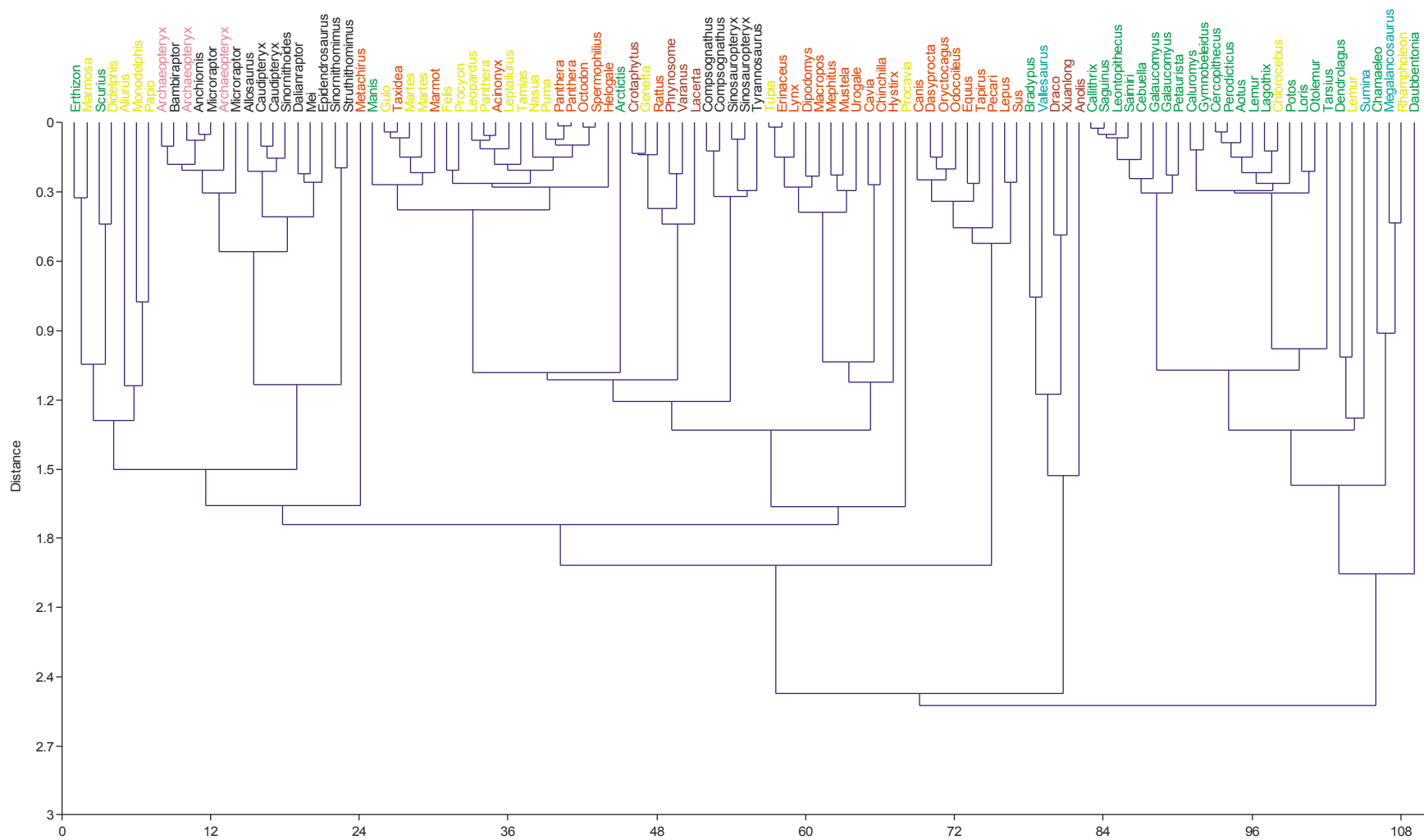

Supplement: Figure S10 — Cluster analysis of quadrupedal data set using forelimb only characters. A) Correlation setting (score 0.7169) B) Euclidean (score 0.7843). Colour coding: Black = theropods, Light blue = Fossil arboreal taxa, Brown = lizards, Green = arboreal mammals and the chameleon, Pink = Archaeopteryx, Red = terrestrial mammals, Yellow = scansorial mammals. (PDF) [file pone.0022292.s010.pdf]

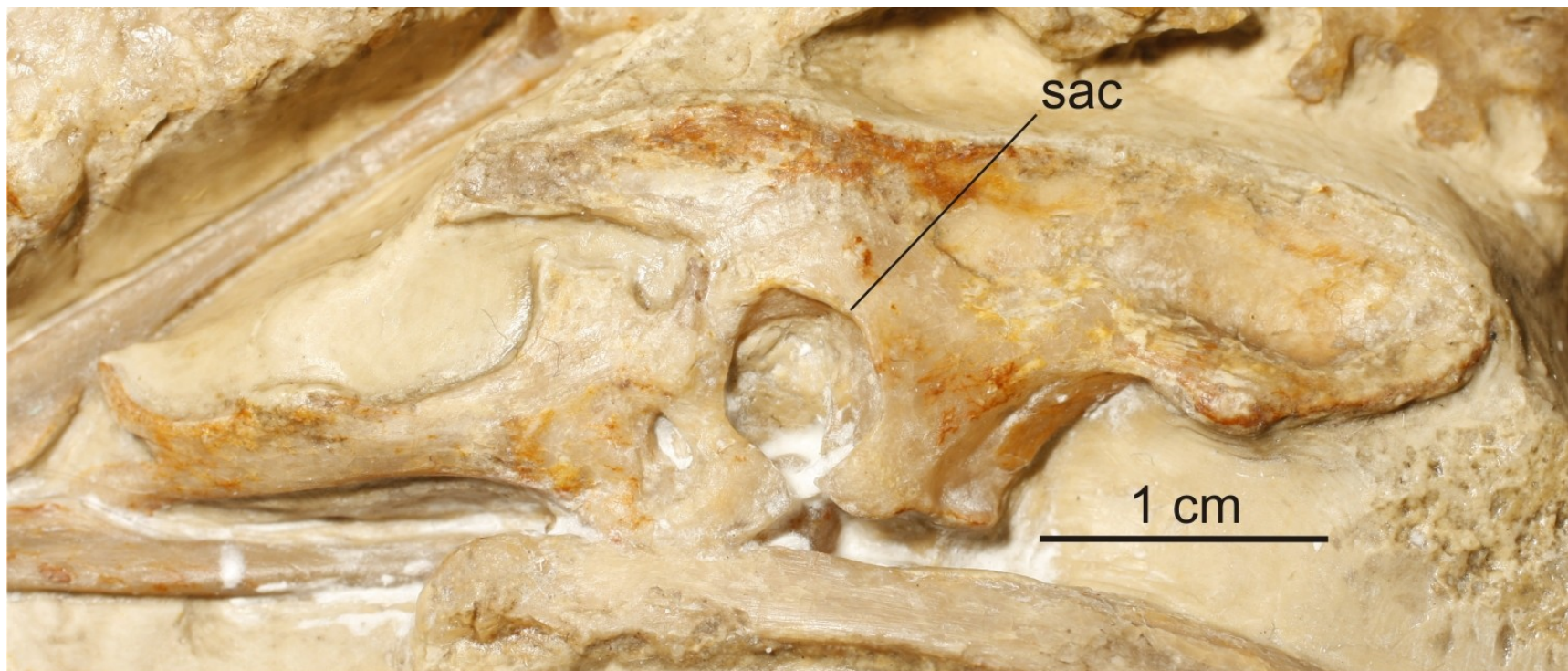

Supplement: Figure S11 — The right ilium in lateral aspect of the London specimen of Archaeopteryx lithographica (BMNH 37001). Note the presence of a well developed supra acetabulum crest (sac), contra [24]. (PDF) [file pone.0022292.s011.pdf]

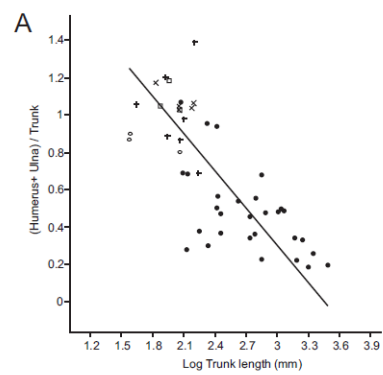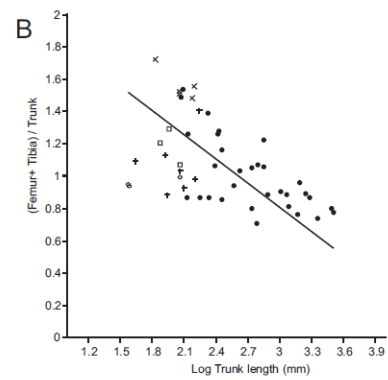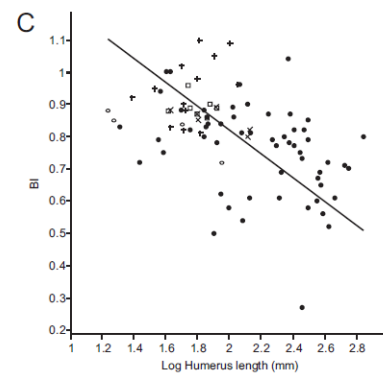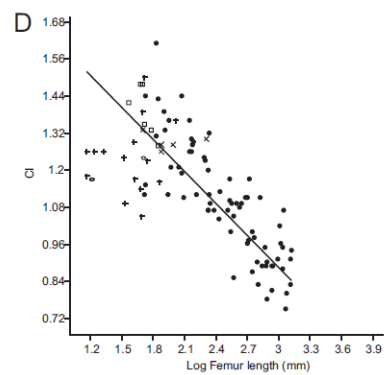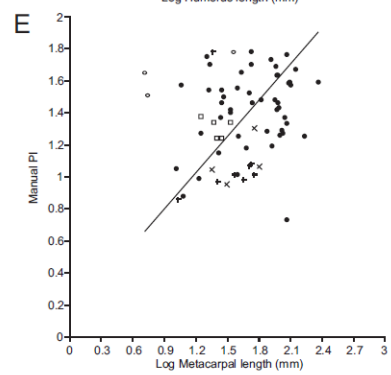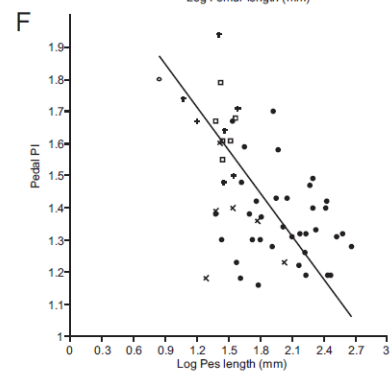

Supplement: Figure S12 — Bivariate plots of metrics used to measure functional morphologies. “Terrestrial” theropods (filled circles), microrpatorines (x's), Archaeopteryx (open squares), avian theropods (crosses), and Scansoriopterygidae (open circles). A) Relative forelimb length (humerus+ulna) to trunk length. LogFL = −0.6642 LogTrunk (+/−0.063783)+2.2913(+/−0.15463), r = −0.76487, P(uncorrelated)<0.001, N = 47. B) Relative hindlimb length to trunk length. LogTL = −0.4968 LogTrunk (+/−0.061771)+2.2919(+/−0.15153), r = −0.52284, P(uncorrelated) = 0.001, N = 49. C) BI regressed against humeral length. LogBI = −0.36873 LogHumerus (+/−0.035123)+1.5559(+/−0.071713), r = −0.48769, P(uncorrelated)<0.001, N = 86. D) CI regressed against log femoral length. LogCI = −0.34557 LogFemur (+/−0.022899)+1.9191(+/−0.052394), r = −0.74601, P(uncorrelated)<0.001, N = 103. E) Manual PI regressed against log metacarpal length. LogPI = −0.7549 LogMetacarpal (+/−0.091001)+0.11887(+/−0.15101), r = −0.16242, P(uncorrelated) = 0.18241, N = 69. F) Pedal PI regressed against log pes length. LogPI = −0.44659 LogPes (+/−0.049735)+2.2501(+/−0.090887), r = −0.5638, P(uncorrelated)<0.001, N = 57. (PDF) [file pone.0022292.s012.pdf]

Taxa less than 1 kg

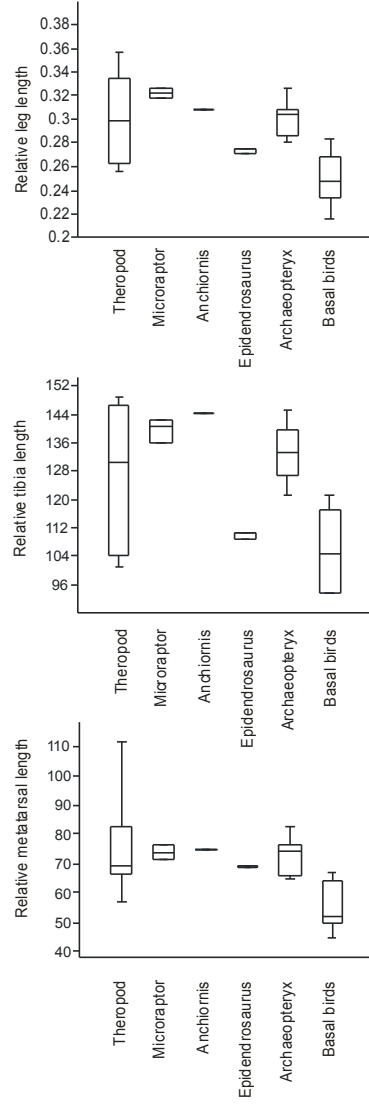

Taxa less than 5 kg

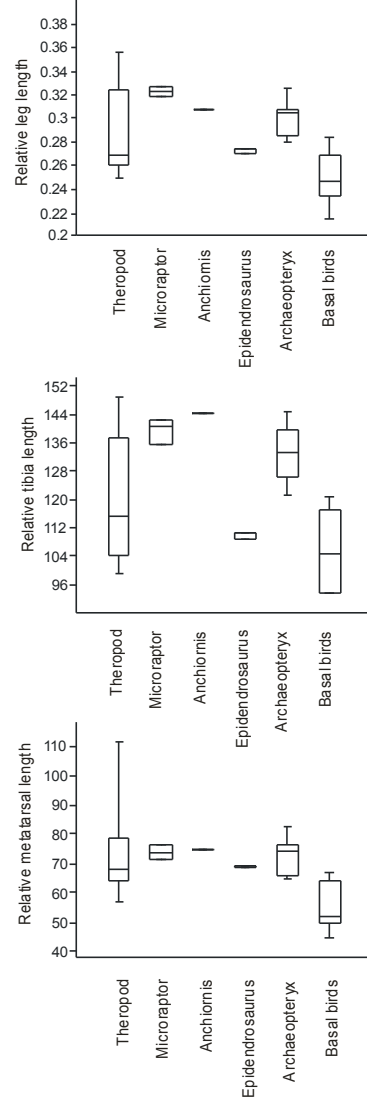

Supplement: Figure S13 — Boxplots comparing within non-avian theropods and basal birds under hindlimb, tibial, and metatarsal indices. (PDF) [file pone.0022292.s013.pdf]
