# Supplementary material for: Assessing Arboreal Adaptations of Bird Antecedents: Testing the Ecological Setting of the Origin of the Avian Flight Stroke
Source: PLoS One. 2011 Aug 9;6(8):e22292. doi: 10.1371/journal.pone.0022292 (PMC3153453; doi:10.1371/journal.pone.0022292)
Supplement: Table S1 — Cluster analysis of total data. A = Arboreal, A fossil = extinct fossil taxa, An = ankle mobility (0 = anterioposterior only, 1 = moderate movement in all 3 planes, 2 = highly mobile),BB = basal bird, BOP = bird of prey, C = climbing bird, CI = Crural index, C = Claw geometry (0 = straight, 1 = recurved, 2 = highly recurved), Fl = relative forelimb length (Humerus+Ulna/trunk), G = ground forager, GB = ground based, Hal = Hallux orientation (0 = in line with other digits, 1 = divergent, 2 = opposable, 3 = zygodactyl), HFR = Hindfoot reversal (0 = no, 1 = yes), Hip = femoral abduction ability (0 = low, little to no abduction occurs, 1 = moderate, limited abduction ability during locomotion, 2 = highly mobile), HL = relative hindlimb length (femur+tibia/trunk), H/U = Humerus divided by ulna, MPI = Manual phalangeal index(non-ungual phalanges/metacarpal), PPI = Pedal phalangeal index (non-ungual phalanges/metatarsals, in theropods PhIII 2+3/Ph III-1), Pol = Pollex orientation(0 = inline, 1 = capable of securing item with a “scissor grip”, 2 = opposable), Pro = forearm pronation/supination (0 = none, 1 = yes), Scan = Scansorial, SH = Shoulder (humerus-glenoid joint mobility) (0 = limited to anterioposterior movement, 1 = moderate movement in all 3 planes, 2 = highly mobile even cricumduction), ST = Stance (0 = plantigrade, 1 = sub ungaligrade, 2 = digigrade), Terr = Terrestrial, Tail = tail prehensile/support ability (0 = none, 1 = yes). For all extant avians and Sinornis, Confuciusornis and Pengornis, given the horizontal position of the femora CI index was taken as the tarsometatarsus/tibia. (PDF) [file pone.0022292.s014.pdf]

category	taxon	FL	St	HL	SH	BI	Pro	Wr	MPI	Pol	Hip	CI	HFR	An	C	PPI	Hal	Tail	Ref
A	*Aotus trivirgatus*	0.67	0	0.9	2	0.91	1	2	1.52	2	2	0.98	0	2	0	0.9	2	0	ROM 94422
A	*Arctictis binturong*	0.71	0	0.77	2	0.79	1	1	1.08	0	1	0.91	1	2	2	?	0	1	[1]
A	*Bradypus tridactylus*	1.12	0	0.59	2	0.82	1	2	2.56	0	1	0.88	1	2	2	2.57	0	0	RM 5027
A	*Callithrix jacchus*	0.71	0	0.93	2	0.88	1	2	1.4	1	2	1.05	0	2	2	0.8	2	0	ROM 101192
A	*Caluromys lanatus*	0.56	0	0.74	2	1.08	1	2	1.57	2	2	1.01	1	2	2	1.44	2	2	ROM 1029
A	*Cebuella pygmea*	0.86	0	1.01	2	0.9	1	2	?	1	2	1.04	0	2	2	?	2	0	ROM 44689
A	*Cercopithecus cephus*	0.73	0	0.9	2	0.97	1	2	1.52	2	2	0.97	0	2	0	1.17	2	0	ROM 57228
A	*Chamaeleo calyptratus*	0.49	0	0.47	2	0.9	1	2	0.87	3	1	0.84	1	2	1	0.85	3	2	ROM 7981
A	*Daubbentonia madagascarensis*	0.78	0	1.06	2	1	1	2	3.41	2	2	0.9	1	2	2	1.49	2	0	RM 2367
A	*Dendrolagus insutus*	0.63	0	0.81	2	1.02	1	1	1.23	1	1	1.07	0	1	2	0.83	0	1	ROM 86035
A	*Erthizon dorsatum*	0.62	0	0.69	1	0.95	1	1	1.2	1	1	0.93	1	2	2	1	2	0	RM 5036
A	*Galaucomyus sabrinus*	0.8	0	0.98	2	1.12	1	2	1.47	1	2	1.23	1	2	2	0.68	0	0	ROM 684
A	*Galaucomyus volans*	0.84	0	1.01	2	1.1	1	2	1.7	1	2	1.13	1	2	2	0.84	0	0	ROM 92110
A	*Gymnobeleidus leadbeateri*	0.63	0	0.89	2	1.13	1	2	1.65	2	2	1.07	0	2	2	1.67	2	0	ROM 101194
A	*Lagothix sp.*	0.94	0	0.96	2	0.87	1	2	1.58	2	2	1.01	0	2	0	1.11	2	2	ROM 196
A	*Lemur fuluvus*	0.8	0	1.16	2	1.06	1	2	1.54	2	2	0.96	0	2	0	1.46	2	0	Rom 68120
A	*Leontopithecus sp.*	0.71	0	0.92	2	0.9	1	2	1.44	1	2	1.02	0	2	2	0.95	2	0	RM 2376
A	*Loris tardigradius*	1.03	0	1.07	2	1.06	1	2	1.68	2	2	0.96	1	2	0	1.52	2	0	RM 2369
A	*Manis tetradactyla*	0.44	0	0.58	1	0.58	1	1	1.35	0	1	0.85	0	2	2	0.72	0	2	RM 5024
A	*Otolemur sp.*	0.82	0	1.14	2	1.03	1	2	1.71	2	1	0.91	1	2	0	?	2	0	ROM 64976
A	*Perodicticus potto*	0.75	0	0.84	2	0.95	1	2	1.49	2	2	0.96	1	2	0	1.72	2	0	ROM 1184
A	*Petaurista grandis*	0.63	0	0.81	2	1.01	1	2	1.67	1	2	1.05	1	2	2	0.86	0	0	ROM 588
A	*Potos flavus*	0.62	0	0.7	2	0.8	1	2	1.45	2	2	0.96	1	2	0	1.07	2	2	ROM 1192
A	*Saguinus sp.*	0.7	0	0.92	2	0.89	1	2	1.38	1	2	1	0	2	2	0.86	2	0	ROM

																			20131
A	*Saimiri sciureus*	0.74	0	0.91	2	0.94	1	2	1.41	1	2	1.02	0	2	2	0.97	2	0	ROM 1027
A	*Scurius carolinesis*	0.54	0	0.72	1	0.95	1	2	1.4	1	2	1.15	1	2	2	0.79	0	0	ROM 87572
A	*Tarsius spectrum*	1.12	1	2.12	2	1.31	1	2	2.41	2	1	1.01	1	2	2	1.57	2	1	RM 2400
A-Fossil	*Megalancosaurus*	0.53	0	0.54	2	0.68	1	2	1.71	3	2	0.62	1	2	2	2.5	2	2	[2]
A-Fossil	*Vallesaurus*	0.4	0	0.55	2	0.6	1	2	2.5	0	2	0.69	?	2	2	3.1	2	2	[3]
A-Fossil	*Sumina*	0.61	0	0.8	2	0.84	1	1	2.32	2	2	0.82	?	1	1	1.6	2	1	[4], pers com.
B-A	*Alcedo atthis*	?	2	?	?	?	?	?	?	?	0	0.36	0	0	2	2.11	2	0	[5,6]
B-A	*Ara severus*	?	2	?	?	?	?	?	?	?	0	0.37	0	0	2	1.96	3	0	[5,6]
B-A	*Chaetura pelagica*	?	2	?	?	?	?	?	?	?	0	0.5	0	0	2	4.32	2	0	[5,6]
B-A	*Coccyzus erythrophthalmus*	?	2	?	?	?	?	?	?	?	0	0.64	0	0	2	2.2	3	0	[5,7]
B-A	*Opisthocomus hoazin*	?	2	?	?	?	?	?	?	?	0	0.62	0	0	2	1.97	2	0	[5,6]
B-C	*Certhia familiaris*	?	2	?	?	?	?	?	?	?	0	0.81	0	0	2	2.16	2	0	[5,8]
B-C	*Dryocopus pileatus*	?	2	?	?	?	?	?	?	?	0	0.67	0	0	2	2.89	3	1	[5.9]
B-C	*Melanerpes erythrocephalus*	?	2	?	?	?	?	?	?	?	0	0.65	0	0	2	2.18	3	1	[5.9]
B-C	*Sitta europaea*	?	2	?	?	?	?	?	?	?	0	0.72	0	0	2	2.29	2	0	[5.10]
B-G	*Cinclus cinclus*	?	2	?	?	?	?	?	?	?	0	0.7	0	0	1	1.9	2	0	[5,11]
B-G	*Columba livia*	?	2	?	?	?	?	?	?	?	0	0.55	0	0	1	1.45	2	0	[5,12]
B-G	*Corvus corax*	?	2	?	?	?	?	?	?	?	0	0.63	0	0	2	2.11	2	0	[5,11]
B-G	*Corvus frugilegus*	?	2	?	?	?	?	?	?	?	0	0.64	0	0	1	1.97	2	0	[5,6]
B-G	*Crotophaga ani*	?	2	?	?	?	?	?	?	?	0	0.69	0	0	1	1.70	3	0	[5,7]
B-G	*Geococcyx sp.*	?	2	?	?	?	?	?	?	?	0	0.74	0	0	1	1.64	3	0	[5,7]
B-G	*Goura crisata*	?	2	?	?	?	?	?	?	?	0	0.74	0	0	1	1.35	2	0	[5,13]
B-G	*Melanocorypha calandra*	?	2	?	?	?	?	?	?	?	0	1	0	0	1	1.43	2	0	[5.10]
B-G	*Pica Pica*	?	2	?	?	?	?	?	?	?	0	0.72	0	0	1	1.93	2	0	[5,6]
B-G	*Sturnus vulgaris*	?	2	?	?	?	?	?	?	?	0	0.66	0	0	1	1.88	2	0	[5,11]
B-G	*Turdus_philomelos*	?	2	?	?	?	?	?	?	?	0	0.71	0	0	1	2.28	2	0	[5.10]
B-GB	*Alectornis sp.*	?	2	?	?	?	?	?	?	?	0	0.56	0	0	1	1.52	1	0	[5,6]
B-GB	*Anhima cornuta*	?	2	?	?	?	?	?	?	?	0	0.68	0	0	0	1.59	1	0	[5,6]
B-GB	*Cariama cristata*	?	2	?	?	?	?	?	?	?	0	0.94	0	0	1	1.05	0	0	[5,6]
B-GB	*Dromaius*	?	2	?	?	?	?	?	?	?	0	0.92	0	0	0	1	0	0	[5,14]

	novaehollandiae																	
B-GB	*Gallus gallus*	?	2	?	?	?	?	?	?	?	0	0.73	0	0	0	1.43	1	0	[5,11]
B-GB	*Meleagris gallopavo*	?	2	?	?	?	?	?	?	?	0	0.73	0	0	1	1.54	1	0	[5,6]
B-GB	*Rhea sp.*	?	2	?	?	?	?	?	?	?	0	0.99	0	0	0	0.9	0	0	[5,14]
B-GB	*Struthio camelus*	?	2	?	?	?	?	?	?	?	0	0.91	0	0	0	0.93	**0**	0	[5,14]
BOP	*Bubo virginanus*	?	2	?	?	?	?	?	?	?	0	0.53	0	0	2	2.9	2	0	[5,15]
BOP	*Buteo jamaicensis*	?	2	?	?	?	?	?	?	?	0	0.77	0	0	2	1.35	2	0	[5,15]
BOP	*Falco sparverius*	?	2	?	?	?	?	?	?	?	0	0.74	0	0	2	1.48	2	0	[5,15]
BOP	*Strix varia*	?	2	?	?	?	?	?	?	?	0	0.56	0	0	2	3.34	2	0	[5,15]
Liz	*Anolis sp.*	0.44	0	0.62	1	0.77	1	2	4.00	0	1	1.00	1	2	2	1.67	0	0	RM
Liz	*Crotaphytus collaris*	0.42	0	0.74	1	0.8	1	2	0.93	0	1	1.01	1	2	2	0.75	0	0	RM
Liz	*Draco sp.*	0.47	0	0.49	1	0.85	1	2	3.00	0	1	0.67	1	2	2	2	0	0	RM 4789
Liz	*Lacerta agilis*	0.22	0	0.23	1	0.63	1	2	1.33	0	1	0.64	1	2	2	2.2	0	0	RM
Liz	*Phrynosome solare*	0.51	0	0.55	1	0.86	1	2	1.33	0	1	1.00	1	2	2	1.2	0	0	RM 4790
Liz	*Varanus niloticus*	0.33	0	0.37	1	0.99	1	2	1.3	0	1	0.78	1	2	2	1	0	0	RM 5003
Liz	*Xuanlong zhaoi*	0.33	0	0.75	1	0.67	1	2	2.57	0	1	0.78	1	2	2	2.15	0	0	[16]
Scan	*Aliurus filgens*	0.57	0	0.62	1	0.79	1	2	1.52	2	2	0.98	0	2	2	1.06	0	0	ROM 663
Scan	*Chlorocebus pygerythrus*	0.92	0	1.05	2	0.98	1	2	1.52	2	2	0.93	0	2	0	1.22	2	0	ROM 011301
Scan	*Didelphis sp.*	0.63	0	0.77	1	1.04	1	2	0.98	1	1	1.05	1	2	2	0.98	2	2	RM 5019
Scan	*Felis catus*	0.68	2	0.8	1	0.99	1	1	0.73	0	1	1.07	0	1	2	0.67	0	0	RM 5071
Scan	*Genetta genetta*	0.52	1	0.65	1	0.8	1	2	1.02	0	1	1.02	0	1	2	0.6	0	0	ROM 114614
Scan	*Gulo gulo*	0.55	0	0.6	1	0.82	1	1	?	0	1	1	0	1	2	?	0	0	[17-19] pers.obs.
Scan	*Lemur catta*	0.8	0	1.18	2	1.04	1	1	1.28	2	1	0.94	0	2	0	0.97	2	0	ROM 114614
Scan	*Leopardus pardal*	0.50	2	0.59	1	1.15	1	1	?	0	1	1.00	0	1	2	?	0	0	[20]
Scan	*Leptailurus serval*	0.49	2	0.62	1	1.04	1	1	?	0	1	1.06	0	1	2	?	0	0	[20]
Scan	*Marmosa mexicana*	0.61	0	0.77	1	1.08	1	1	1.5	1	1	1.12	0	1	2	1.3	2	2	ROM 96318
Scan	*Martes americana*	0.48	0	0.62	1	0.78	1	1	1.42	0	2	1.15	1	2	2	0.69	0	0	RM 621
Scan	*Martes pennanti*	0.5	0	0.62	1	0.78	1	1	1.22	0	2	1.09	1	2	2	0.81	0	0	RM 623
Scan	*Monodelphis sp.*	0.52	0	0.65	1	0.93	1	1	0.94	2	1	1.06	0	2	2	0.92	2	2	ROM 98909
Scan	*Nasua narica*	0.63	0	0.7	1	1	1	1	0.89	0	2	0.97	0	2	1	0.82	0	0	ROM 749

Scan	*Panthera pardus*	0.55	2	0.71	1	1.09	1	1	?	0	1	1.20	0	1	2	?	0	0	[20]
Scan	*Papio papio*	1.04	0	1.13	1	0.98	1	1	1.52	2	1	0.93	0	1	0	0.91	2	0	ROM 88612
Scan	*Procavia capensis*	0.46	0	0.5	0	0.58	0	2	0.83	0	0	0.96	0	1	0	0.83	0	0	RM 4073
Scan	*Procyon lotor*	0.79	0	0.92	1	1.03	1	1	0.9	0	2	1.1	0	2	1	0.82	0	0	RM 624
Scan	*Puma concolor*	0.52	2	0.69	1	0.98	1	1	?	0	1	1.03	0	1	2	?	0	0	[20]
Scan	*Rhampholeon brevicaudatus*	0.83	0	0.74	2	0.99	1	2	1.76	3	1	1.04	1	2	1	1.76	3	0	RM uncat.
Scan	*Tamias minus*	0.68	0	0.96	1	1.05	1	1	1.17	0	2	1.22	0	2	1	0.7	0	0	ROM 82664
Scan	*Tupia ferruginea (gillis)*	0.64	0	0.84	0	0.88	1	1	0.81	0	0	1.08	0	1	2	0.61	0	0	RM 3053
Terr	*Acinonyx jubatus*	0.57	2	0.67	1	1.14	1	1	?	0	1	1.20	0	0	1	?	0	0	[20]
Terr	*Canis familiaris*	0.72	2	0.78	0	1	0	0	0.72	0	0	1.02	0	0	1	0.67	0	0	Rm 7212
Terr	*Cavia porcellus*	0.45	0	0.6	0	0.89	0	1	1.02	0	0	1.03	0	1	1	0.59	0	0	ROM R525
Terr	*Chinchilla sp.*	0.64	0	1.04	0	1.07	0	1	0.95	0	0	1.37	0	0	1	0.71	0	0	ROM 742
Terr	*Dasyprocta sp.*	0.56	2	0.71	0	0.84	0	0	0.71	0	0	1.2	0	0	0	0.72	0	0	RM 2460
Terr	*Dipodomys ordii*	0.65	0	1.26	0	1.2	1	1	0.9	0	0	1.36	0	0	1	0.82	0	0	ROM 91990
Terr	*Equus caballus*	0.48	1	0.55	0	1.14	0	0	0.41	0	0	0.93	0	0	0	0.41	0	0	ROM R 929
Terr	*Erinaceus europaneus*	0.62	0	0.69	0	0.89	1	1	0.81	0	0	1.2	0	1	1	0.74	0	0	RM 2503
Terr	*Helogale parvula*	0.52	1	0.59	1	0.85	1	1	0.63	0	1	1.03	0	0	1	0.55	0	0	ROM 58388
Terr	*Hystirx cristata*	0.51	0	0.61	0	0.77	1	1	0.85	1	0	0.97	0	1	0	0.73	1	0	RM 2464
Terr	*Lepus americannus*	0.57	2	0.81	0	1.01	0	0	1.05	0	0	1.17	0	0	0	0.81	0	0	RM 5029
Terr	*Lynx lynx*	0.69	2	0.89	0	1.02	1	1	0.84	0	1	1	0	1	2	0.76	0	0	RM 5070
Terr	*Macropos sp*	0.45	2	1	0	1.11	1	1	0.82	0	0	1.32	0	0	0	0.78	0	0	RM 3052
Terr	*Marmot monax*	0.49	0	0.63	1	0.76	1	1	1.05	0	1	1.02	0	1	1	0.92	0	0	RM 620
Terr	*Mephitus mephistus*	0.54	0	0.68	0	0.77	1	1	1	0	0	1.02	0	1	1	0.69	0	0	RM 631
Terr	*Metachirus sp.*	0.59	0	0.88	0	1.02	0	1	0.87	2	0	1.09	0	1	0	0.72	1	0	ROM 114155
Terr	*Mustela erminea*	0.42	0	0.55	0	0.7	1	1	1.18	0	0	1.08	0	1	2	0.73	0	0	RM 4043
Terr	*Octodon degu*	0.48	0	0.67	1	0.9	1	1	0.95	0	0	1.14	0	0	1	0.78	0	0	ROM R4045
Terr	*Odocoileus sp.*	0.53	1	0.65	0	0.91	0	0	0.57	0	0	0.77	0	0	0	0.53	0	0	RM 7211
Terr	*Oryctocagus sp.*	0.48	2	0.72	0	0.92	0	0	0.81	0	0	1.17	0	0	0	0.8	0	0	RM 5031
Terr	*Panthera leo*	0.55	2	0.67	1	0.93	1	1	?	0	1	1.03	0	1	2	?	0	0	[20]

Terr	*Panthera tigris*	0.56	2	0.69	1	0.92	1	1	?	0	1	1.12	0	1	2	?	0	0	[20]
Terr	*Pecari ta jacu*	0.34	1	0.47	0	0.63	0	0	0.53	0	0	0.95	0	0	0	0.47	0	0	RM 2461
Terr	*Rattus sp.*	0.46	0	0.64	1	0.92	1	2	0.99	0	1	1.11	0	1	1	0.43	0	0	RM 7213
Terr	*Spermophilius franklini*	0.46	0	0.61	1	0.9	1	1	0.95	0	1	1.05	0	1	1	0.7	0	0	Rm 2611
Terr	*Sus sp.*	0.54	1	0.69	0	0.77	0	0	1.15	0	0	0.92	0	0	0	0.69	0	0	RM 6026
Terr	*Tapirus sp.*	0.43	1	0.58	0	1.13	0	0	0.67	0	0	1.21	0	0	0	0.59	0	0	RM 256
Terr	*Taxidea taxus*	0.55	0	0.54	1	0.78	1	1	1.27	0	1	0.76	0	1	0	0.96	0	0	ROM 722
Terr	*Urogale everetti*	0.59	0	0.78	0	0.99	1	1	1.11	0	0	1.1	0	1	2	0.79	0	0	ROM 1183
BB	*Archaeopteryx*	1.03	2	1.07	1	0.87	0	1	1.24	1	0	1.36	0	0	1	1.79	0	0	[21]
BB	*Archaeopteryx*	1.05	2	1.21	1	0.88	0	1	1.38	1	0	1.43	0	0	1	1.67	0	0	[21]
BB	*Archaeopteryx*	1.18	2	1.29	1	0.96	0	1	1.24	1	0	1.54	0	0	1	1.55	0	0	[21]
BB	*Confuciusornis*	?	2	?	?	?	?	?	?	?	0	0.49	0	0	1	1.67	2	0	[7]
BB	*Jeholornis*	?	2	?	?	?	?	?	?	?	0	1.1	0	0	1	1.65	2	0	[7]
BB	*Pengornis*	?	2	?	?	?	?	?	?	?	0	0.53	0	0	2	1.94	2	0	[22]
BB	*Sapeornis*	?	2	?	?	?	?	?	?	?	0	1.15	0	0	2	1.7	2	0	[7]
BB	*Sinornis*	?	2	?	?	?	?	?	?	?	0	0.55	0	0	1	1.74	2	0	[23]
Ther	*Allosaurus*	0.33	2	0.89	1	0.85	0	1	1.57	1	0	0.81	0	0	1	1.42	0	0	[24]
Ther	*Anchiornis*	1.07	2	1.48	1	0.8	0	1	1.42	1	0	1.61	0	0	1	1.67	0	0	[25]
Ther	*Bambiraptor*	0.95	2	1.38	1	0.89	0	1	1.18	1	0	1.44	0	0	1	1.16	0	0	[26]
Ther	*Caudipteryx*	0.45	2	1.14	1	0.88	0	1	1.54	1	0	1.26	0	0	0	1.3	0	0	Pers. com Zhonghe
Ther	*Caudipteryx*	0.51	2	1.27	1	0.86	0	1	1.46	1	0	1.3	0	0	0	1.42	0	0	Pers. com. Zhonghe
Ther	*Compsognathus*	0.38	2	0.86	1	0.75	0	1	1.27	0	0	1.33	0	0	1	1.48	0	0	[27], pers. com
Ther	*Compsognathus*	0.36	2	0.85	1	0.82	0	1	1.37	0	0	1.21	0	0	1	1.48	0	0	[27], pers. com
Ther	*Dalianraptor*	0.86	2	1.03	1	0.88	0	1	1.74	1	0	1.39	0	0	1	1.71	0	0	[28]
Ther	*Epidendrosaurus*	0.9	2	0.94	1	0.85	0	1	1.51	1	0	1.17	0	0	1	1.8	0	0	[29]
Ther	*Mei long*	0.69	2	1.53	1	1	0	1	?	1	0	1.31	0	0	1	?	0	0	[30]
Ther	*Microraptor gui*	1.1	2	1.59	1	0.84	0	1	?	1	0	1.28	0	0	1	1.4	0	0	[7] pers. obs.
Ther	*Microraptor zhaoianus*	1.04	2	1.53	1	0.85	0	1	0.95	1	0	1.28	0	0	1	1.39	0	0	[31]
Ther	*Sinornithoides*	0.56	2	1.28	1	0.78	0	1	1.4	1	0	1.41	0	0	0	1.3	0	0	[32]
Ther	*Sinornithomimus*	0.47	2	0.88	1	0.69	0	0	1.46	1	0	1.07	0	0	0	1.38	0	0	[33]

Ther	*Sinosauropteryx*	0.28	2	0.86	1	0.83	0	1	1.05	0	0	1.18	0	0	1	1.38	0	0	Pers. com. Currie
Ther	*Sinosauropteryx*	0.3	2	0.86	1	0.79	0	1	0.99	0	0	1.16	0	0	1	1.23	0	0	Pers. com Currie
Ther	*Struthithomimus*	0.48	2	0.88	1	0.79	0	0	1.29	1	0	1.11	0	0	0	1.19	0	0	[34]
Ther	*Tyrannosaurus*	0.2	2	0.86	1	0.56	0	1	1.15	0	0	0.94	0	0	1	1.28	0	0	Pers. com Currie
