# Supplementary material for: Assessing Arboreal Adaptations of Bird Antecedents: Testing the Ecological Setting of the Origin of the Avian Flight Stroke
Source: PLoS One. 2011 Aug 9;6(8):e22292. doi: 10.1371/journal.pone.0022292 (PMC3153453; doi:10.1371/journal.pone.0022292)
Supplement: Table S2 — Cluster analysis of bird data. A = arboreal forager, BB = basal birds, Bop = bird of prey, C = climbing birds, Claw = Claw geometry (0 = straight, 1 = recurved, 2 = highly recurved), G = ground forager, GB = ground based, Hallux (hallux 0 = non reversed, 1 = reversed but raised, 2 = reversed), Leg L = relative leg length (F+T+Tmt/mass∧0.33), PPI = Pedal phalangeal index (Ph II+III/PhI), TF = tail feathers show adaption for weight supporting adaptation (0 = absent, present = 1), TL = relative tibia length, TMTL = relative tarsometatarsal length, Zy = zygodactyls = (0 = absent, 1 = present). (PDF) [file pone.0022292.s015.pdf]

category	taxon	Leg L	PPI	TL	Claw	Hallux	Zy	TF	TMTL
A	*Ara macao*	0.18	1.96	0.78	2	2	1	0	0.40
A	*Chaetura pelagica*	0.18	4.32	0.76	2	2	1	1	0.54
A	*Opisthocomus hoazin*	0.23	1.97	0.91	1	2	0	0	0.80
A	*Alcedo atthis*	0.17	2.11	0.78	2	2	0	0	0.39
A	*Coccyzus erythrophthalmus*	0.25	2.20	1.00	1	2	1	0	0.91
BOP	*Bubo virginanus*	0.25	2.90	1.06	2	2	1	0	0.79
BOP	*Buteo jamaicensis*	0.28	1.35	1.02	2	2	0	0	1.11
BOP	*Falco sparverius*	0.25	1.48	0.95	2	2	0	0	1.00
BOP	*Strix varia*	0.27	3.37	1.12	2	2	1	0	0.89
C	*Certhia familiaris*	0.21	2.16	0.84	2	2	0	1	0.96
C	*Dryocopus pileatus*	0.19	2.53	0.74	2	2	1	1	0.71
C	*Melanerpes erythrocephalus*	0.23	2.18	0.91	2	2	1	1	0.86
C	*Sitta europaea*	0.22	2.29	0.87	2	2	0	0	0.89
G	*Cinclus cinclus*	0.24	1.90	1.01	2	1	0	0	1.01
G	*Columba livia*	0.19	1.45	0.78	1	2	0	0	0.61
G	*Corvus corax*	0.25	2.11	1.04	2	2	0	0	0.92
G	*Corvus frugilegus*	0.25	1.97	1.05	1	2	0	0	0.96
G	*Crotophaga ani*	0.21	1.70	0.85	1	2	1	0	0.84
G	*Geococcyx sp.*	0.28	1.63	1.10	1	2	1	0	1.15
G	*Goura crisata*	0.23	1.35	0.92	1	2	0	0	0.97
G	*Melanocorypha calandra*	0.25	1.43	0.87	1	2	0	0	1.25
G	*Pica pica*	0.26	1.93	1.05	1	2	0	0	1.08
G	*Sturnus vulgaris*	0.23	1.88	0.97	1	2	0	0	0.91
G	*Turdus philomelos*	0.25	2.28	1.03	1	2	0	0	1.04
GB	*Alectoris rufa*	0.21	1.52	0.85	1	1	0	0	0.68
GB	*Anhima cornuta*	0.28	1.59	1.18	1	1	0	0	1.15
GB	*Cariama cristata*	0.43	1.05	1.71	1	0	0	0	2.30
GB	*Dromaius novaehollandiae*	0.30	1.00	1.14	0	0	0	0	1.49

GB	*Gallus gallus*	0.31	1.42	1.20	1	1	0	0	1.26
GB	*Meleagris gallopavo*	0.27	1.54	1.09	1	1	0	0	1.13
GB	*Rhea sp.*	0.29	0.90	1.04	0	0	0	0	1.46
GB	*Struthio camelus*	0.27	0.93	1.04	0	0	0	0	1.34
BB	*Confuciusornis*	0.21	1.67	0.87	1	2	0	0	0.73
BB	*Jeholornis*	0.26	1.65	1.07	1	2	0	0	0.96
BB	*Pengornis*	0.23	1.94	0.88	1	2	0	0	0.82
BB	*Sapeornis*	0.23	1.70	0.87	2	2	0	0	0.83
BB	*Sinornis*	0.28	1.74	1.13	1	2	0	0	1.11
BB	*Archaeopteryx*	0.29	1.79	1.19	1	0	0	0	1.10
BB	*Archaeopteryx*	0.30	1.67	1.26	1	0	0	0	1.28
BB	*Archaeopteryx*	0.33	1.55	1.37	1	0	0	0	1.38
Ther	*Anchiornis*	0.31	1.67	1.36	1	0	0	0	1.25
Ther	*Bambiraptor*	0.27	1.16	1.19	1	0	0	0	1.00
Ther	*Caudipteryx*	0.26	1.30	1.04	0	0	0	0	1.12
Ther	*Caudipteryx*	0.27	1.42	1.07	0	0	0	0	1.13
Ther	*Compsognathus*	0.26	1.49	0.67	1	0	0	0	0.76
Ther	*Dalianraptor*	0.31	1.71	1.23	1	0	0	0	1.38
Ther	*Mei_long*	0.33	1.28	1.38	1	0	0	0	1.32
Ther	*Microraptor gui*	0.32	1.40	1.28	1	0	0	0	1.28
Ther	*Microraptor zhaoianus*	0.33	1.39	1.33	1	0	0	0	1.21
Ther	*Sinornithoides*	0.27	1.30	1.11	1	0	0	0	1.15
Ther	*Sinosauropteryx*	0.16	1.23	0.95	1	0	0	0	1.11
Ther	*Struthithomimus*	0.27	1.40	0.99	0	0	0	0	1.19
