# Supplementary material for: Assessing Arboreal Adaptations of Bird Antecedents: Testing the Ecological Setting of the Origin of the Avian Flight Stroke
Source: PLoS One. 2011 Aug 9;6(8):e22292. doi: 10.1371/journal.pone.0022292 (PMC3153453; doi:10.1371/journal.pone.0022292)
Supplement: Table S3 — PCO loadings for first 3 axes for total dataset. Percentage of variance explained by the first three axes for Euclidean setting: 39.0, 23.9 and 10.3%. For Correlation setting: 35.3, 16.7 and 7.4%. All other axes explain less than 5% of the variance. (PDF) [file pone.0022292.s016.pdf]

		Euclidean				Correlation		
category	taxon	axis 1	axis 2	axis 3		axis 1	axis 2	axis 3
A	*Aotus trivirgatus*	2.3294	-1.2625	1.922		0.57886	-0.41539	0.32696
A	*Arctictis binturong*	2.4596	0.57812	-1.3004		0.80383	0.53612	-0.09461
A	*Bradypus tridactylus*	2.0203	-0.28325	-1.7099		0.40516	0.22547	-0.02093
A	*Callithrix jacchus*	2.4658	-1.3621	-0.0421		0.63249	-0.1907	-0.23175
A	*Caluromys lanatus*	2.924	-2.115	0.19749		0.96395	-0.37629	-0.23613
A	*Cebuella pygmea*	2.5287	-1.8346	-0.71783		0.61983	-0.2137	-0.27367
A	*Cercopithecus cephus*	2.2601	-1.3777	1.8395		0.55177	-0.42795	0.31798
A	*Chamaeleo calyptratus*	2.1176	-2.8174	1.8169		0.66002	-0.88486	-0.05785
A	*Daubbentonia madagascarensis*	2.186	-2.5395	-0.32231		0.59167	-0.24926	-0.04673
A	*Dendrolagus insutus*	1.6972	0.42659	-0.31757		0.52967	0.25494	-0.10692
A	*Erthizon dorsatum*	1.8575	-0.93637	-0.1578		0.63178	-0.29107	-0.41743
A	*Galaucomyus sabrinus*	3.1129	0.37787	-0.39315		0.77099	0.3878	0.16621
A	*Galaucomyus volans*	3.0568	0.25418	-0.46963		0.75339	0.37168	0.16461
A	*Gymnobeleidus leadbeateri*	2.2333	-2.1307	0.038403		0.54157	-0.30228	-0.15902
A	*Lagothix sp.*	2.7328	-1.4395	1.98		0.71188	-0.51985	0.42752
A	*Lemur fuluvus*	2.1834	-1.5077	1.7584		0.5116	-0.43302	0.32642
A	*Leontopithecus sp.*	2.424	-1.4357	-0.09327		0.62039	-0.19827	-0.2348
A	*Loris tardigradius*	2.3615	-1.5675	1.69		0.60476	-0.45878	0.42401
A	*Manis tetradactyla*	2.4837	1.0588	-0.7237		0.70014	0.41966	-0.18354
A	*Otolemur sp.*	1.9598	-1.6099	1.4967		0.57521	-0.48691	0.42893
A	*Perodicticus potto*	2.3491	-1.5778	1.6142		0.59743	-0.47999	0.37187
A	*Petaurista grandis*	3.0556	0.25718	-0.49698		0.76802	0.34828	0.13337
A	*Potos flavus*	2.9378	-1.3953	1.9426		0.89549	-0.54452	0.50984
A	*Saguinus sp.*	2.4443	-1.3914	-0.06116		0.63035	-0.19821	-0.24056
A	*Saimiri sciureus*	2.4174	-1.4415	-0.09536		0.61864	-0.19873	-0.23613
A	*Scurius carolinesis*	3.0235	0.6457	-0.49206		0.77478	0.3806	0.09356
A	*Tarsius spectrum*	1.1149	-2.662	-0.10006		0.31697	-0.38091	-0.13047
A-Fossil	*Megalancosaurus*	2.5753	-3.0112	0.12878		0.70873	-0.56086	-0.18477
A-Fossil	*Vallesaurus*	2.8774	-2.0786	-1.5839		0.64945	-0.16119	-0.26368
A-Fossil	*Sumina*	1.7684	-1.9311	0.87226		0.64322	-0.64441	0.12978
B-A	*Alcedo atthis*	-2.4966	-1.6879	-0.98055		-0.38891	-0.22156	-0.32469
B-A	*Ara severus*	-2.6105	-2.8438	-0.38283		-0.33948	-0.43917	-0.29817
B-A	*Chaetura pelagica*	-2.9013	-2.6111	-2.6561		-0.32131	-0.19841	-0.16905
B-A	*Coccyzus erythrophthalmus*	-2.6432	-2.9099	-0.50574		-0.34705	-0.41427	-0.25705
B-A	*Opisthocomus hoazin*	-2.4504	-1.5804	-0.89356		-0.40394	-0.20137	-0.31234
B-C	*Certhia familiaris*	-2.4781	-1.6368	-0.99034		-0.40411	-0.18571	-0.27875
B-C	*Dryocopus pileatus*	-2.6033	-3.4454	-0.86321		-0.38835	-0.48295	-0.26971
B-C	*Melanerpes erythrocephalus*	-2.4624	-3.1309	-0.41097		-0.40754	-0.51241	-0.33093
B-C	*Sitta europaea*	-2.5106	-1.7109	-1.0701		-0.39686	-0.19223	-0.27694
B-G	*Cinclus cinclus*	-2.5735	-1.1997	0.40802		-0.51553	-0.37519	-0.06047
B-G	*Columba livia*	-2.4857	-1.0118	0.61924		-0.53432	-0.40476	-0.10364

B-G	*Corvus corax*	-2.4791	-1.6434	-0.97001		-0.39987	-0.19991	-0.29969
B-G	*Corvus frugilegus*	-2.5916	-1.2409	0.36974		-0.50961	-0.37837	-0.06261
B-G	*Crotophaga ani*	-2.6764	-2.3323	1.0261		-0.4424	-0.59629	-0.0667
B-G	*Geococcyx sp.*	-2.6612	-2.2979	1.0551		-0.44524	-0.59436	-0.06488
B-G	*Goura crisata*	-2.4513	-0.93253	0.67072		-0.54536	-0.38853	-0.08907
B-G	*Melanocorypha calandra*	-2.4534	-0.92966	0.64725		-0.54654	-0.35926	-0.05609
B-G	*Pica pica*	-2.5791	-1.2115	0.39176		-0.51419	-0.37225	-0.05675
B-G	*Sturnus vulgaris*	-2.5718	-1.1969	0.41673		-0.51546	-0.37958	-0.06552
B-G	*Turdus_philomelos*	-2.6526	-1.3741	0.19499		-0.49244	-0.36138	-0.03995
B-GB	*Alectornis sp.*	-2.359	0.18276	0.096851		-0.60091	-0.0787	-0.13217
B-GB	*Anhima cornuta*	-2.5142	0.50377	1.3544		-0.6866	-0.28847	0.23831
B-GB	*Cariama cristata*	-2.0928	1.6985	-0.15937		-0.63336	0.35749	-0.12719
B-GB	*Dromaius novaehollandiae*	-2.2302	2.0543	1.1428		-0.7267	0.16465	0.27397
B-GB	*Gallus gallus*	-2.4759	0.58834	1.4292		-0.70425	-0.28623	0.24268
B-GB	*Meleagris gallopavo*	-2.3515	0.20335	0.096991		-0.60305	-0.06143	-0.11228
B-GB	*Rhea sp.*	-2.2027	2.1151	1.1816		-0.72886	0.17818	0.28353
B-GB	*Struthio camelus*	-2.2147	2.0872	1.1672		-0.7329	0.16878	0.27427
BOP	*Bubo virginanus*	-2.6441	-2.0142	-1.474		-0.36887	-0.20373	-0.248
BOP	*Buteo jamaicensis*	-2.3064	-1.2626	-0.58896		-0.42437	-0.19153	-0.34955
BOP	*Falco sparverius*	-2.3371	-1.3299	-0.64708		-0.42104	-0.19361	-0.34191
BOP	*Strix varia*	-2.7254	-2.1989	-1.7999		-0.35443	-0.19967	-0.21707
Liz	*Anolis sp.*	1.675	-0.1446	-1.8561		0.46238	0.32074	0.071733
Liz	*Crotaphytus collaris*	2.2848	0.95174	-0.87062		0.66127	0.43797	-0.09302
Liz	*Draco sp.*	1.9182	0.06	-1.6658		0.46006	0.27151	-0.02877
Liz	*Lacerta agilis*	2.0022	0.3354	-1.4648		0.49309	0.23013	-0.17558
Liz	*Phrynosome solare*	2.2044	0.71775	-1.0466		0.5982	0.39175	-0.08576
Liz	*Varanus niloticus*	2.2225	0.77106	-1.0061		0.64108	0.38496	-0.11061
Liz	*Xuanlong zhaoi*	1.9821	0.14133	-1.6032		0.45016	0.26648	-0.04665
Scan	*Aliurus filgens*	2.6998	0.1	-0.16914		0.6435	0.14843	0.076766
Scan	*Chlorocebus pygerythrus*	2.2338	-1.4187	1.8382		0.54013	-0.43371	0.32722
Scan	*Didelphis sp.*	2.4108	-1.1875	-0.04787		0.86152	-0.29345	-0.48073
Scan	*Felis catus*	0.16036	1.0575	-0.97777		-0.15032	0.49778	-0.22171
Scan	*Genetta genetta*	0.83726	0.86224	-0.91819		0.18318	0.38311	-0.18854
Scan	*Gulo gulo*	1.3103	0.92071	-1.5624		0.44034	0.38843	-0.29684
Scan	*Lemur catta*	1.606	-1.0841	1.9724		0.4316	-0.55382	0.31902
Scan	*Leopardus pardal*	-0.03827	0.74178	-1.8205		-0.1532	0.47029	-0.24069
Scan	*Leptailurus serval*	-0.02874	0.75526	-1.8167		-0.15293	0.4722	-0.23912
Scan	*Marmosa mexicana*	1.442	-1.1635	-0.10152		0.43701	-0.38102	-0.43655
Scan	*Martes americana*	2.8319	1.1932	-0.83883		0.73528	0.50398	0.001666
Scan	*Martes pennanti*	2.7794	1.1565	-0.85745		0.73035	0.48911	-0.03385
Scan	*Monodelphis sp.*	2.0847	-1.3174	0.43129		0.64161	-0.47854	-0.35993
Scan	*Nasua narica*	2.4412	1.4006	-0.00592		0.67236	0.38482	0.22336
Scan	*Panthera pardus*	-0.01254	0.77499	-1.7996		-0.16659	0.49003	-0.21697

Scan	*Papio papio*	0.92439	-0.8337	1.9485		0.26079	-0.69062	0.34413
Scan	*Procavia capensis*	0.54337	1.7916	0.81971		0.26615	0.16858	0.4128
Scan	*Procyon lotor*	2.4477	1.4059	0.019856		0.64608	0.40932	0.2608
Scan	*Puma concolor*	-0.02896	0.75175	-1.8126		-0.15564	0.47471	-0.24103
Scan	*Rhampholeon brevicaudatus*	1.5361	-3.1912	1.3529		0.42763	-0.72843	0.012076
Scan	*Tamias minus*	2.5235	1.4531	0.042856		0.6481	0.43121	0.30507
Scan	*Tupia ferruginea (gillis)*	0.73518	1.4902	-0.74623		0.20573	0.38069	-0.32655
Terr	*Acinonyx jubatus*	-0.6936	1.1195	-0.86671		-0.36951	0.42352	0.035536
Terr	*Canis familiaris*	-1.5072	1.798	-0.04235		-0.63294	0.36682	-0.14802
Terr	*Cavia porcellus*	0.51549	1.7883	0.11012		0.23041	0.33741	0.05678
Terr	*Chinchilla sp.*	0.000157	1.8518	0.17897		-0.05118	0.29299	0.02498
Terr	*Dasyprocta sp.*	-1.4643	2.144	0.75976		-0.6673	0.22502	0.21573
Terr	*Dipodomys ordii*	0.081164	1.7099	0.072099		-0.06747	0.31041	-0.01021
Terr	*Equus caballus*	-0.92638	2.2845	0.9351		-0.57642	0.23865	0.27129
Terr	*Erinaceus europaneus*	0.60463	1.6812	0.006049		0.20139	0.36369	0.002566
Terr	*Helogale parvula*	0.096755	1.4492	-0.0158		-0.10282	0.41218	0.002835
Terr	*Hystirx cristata*	0.47451	0.81426	1.3758		0.20541	-0.38534	0.29298
Terr	*Lepus americannus*	-1.4498	2.0679	0.72565		-0.63358	0.21592	0.22661
Terr	*Lynx lynx*	-0.08158	1.1756	-1.0482		-0.20669	0.52046	-0.25906
Terr	*Macropos sp*	-1.0558	1.935	0.63341		-0.54628	0.27904	0.24329
Terr	*Marmot monax*	1.2118	1.3011	-0.0482		0.44502	0.35606	0.10829
Terr	*Mephitus mephistus*	0.62429	1.664	-0.00187		0.23257	0.34567	-0.01386
Terr	*Metachirus sp.*	0.30184	0.50685	1.8974		0.10705	-0.50891	0.42882
Terr	*Mustela erminea*	0.73064	1.4054	-0.82981		0.22701	0.33964	-0.31565
Terr	*Octodon degu*	0.32546	1.5431	0.067904		0.05296	0.23733	-0.04654
Terr	*Odocoileus sp.*	-0.93753	2.2072	0.90234		-0.60563	0.22035	0.26159
Terr	*Oryctocagus sp.*	-1.477	2.098	0.73019		-0.65252	0.21696	0.21179
Terr	*Panthera leo*	-0.0281	0.75194	-1.8133		-0.15483	0.47401	-0.24407
Terr	*Panthera tigris*	-0.01878	0.76576	-1.8068		-0.1589	0.48037	-0.23424
Terr	*Pecari ta jacu*	-0.93296	2.2561	0.90444		-0.58005	0.24465	0.26681
Terr	*Rattus sp.*	1.4643	1.315	0.062325		0.53536	0.39642	0.19597
Terr	*Spermophilius franklini*	1.2671	1.4109	0.023044		0.47945	0.39745	0.13105
Terr	*Sus sp.*	-0.90311	2.1032	0.8414		-0.50207	0.19103	0.28248
Terr	*Tapirus sp.*	-0.91369	2.2261	0.89785		-0.5114	0.23948	0.29285
Terr	*Taxidea taxus*	1.2465	1.5613	0.72921		0.46489	0.27602	0.53811
Terr	*Urogale everetti*	0.71822	1.3857	-0.81718		0.18939	0.36185	-0.29248
BB	*Archaeopteryx*	-1.0707	0.77908	0.013525		-0.43758	0.11094	0.091936
BB	*Archaeopteryx*	-1.0471	0.80289	0.058802		-0.43616	0.12088	0.10693
BB	*Archaeopteryx*	-1.0373	0.86288	0.13212		-0.45536	0.13791	0.11768
BB	*Confuciusornis*	-2.5377	-1.1264	0.51507		-0.52075	-0.40289	-0.09566
BB	*Jeholornis*	-2.4947	-1.0165	0.55283		-0.53666	-0.34382	-0.03461
BB	*Pengornis*	-2.4499	-1.5813	-0.88123		-0.40134	-0.20888	-0.32359
BB	*Sapeornis*	-2.3595	-1.3669	-0.72968		-0.42642	-0.15987	-0.28016

BB	*Sinornis*	-2.5489	-1.1495	0.48323		-0.51948	-0.39474	-0.08495
Ther	*Allosaurus*	-1.0349	0.8183	0.049029		-0.37717	0.061911	0.054699
Ther	*Anchiornis*	-1.0485	0.80719	0.081756		-0.43553	0.12832	0.12396
Ther	*Bambiraptor*	-0.97202	1.0098	0.2601		-0.45526	0.1424	0.11469
Ther	*Caudipteryx*	-0.93858	1.2508	0.95466		-0.43071	-0.0157	0.37636
Ther	*Caudipteryx*	-0.96924	1.2152	0.93489		-0.44103	-0.01432	0.37657
Ther	*Compsognathus*	-1.1314	1.1873	-0.31904		-0.39438	0.25343	-0.01776
Ther	*Compsognathus*	-1.1387	1.1562	-0.33516		-0.38973	0.24613	-0.01962
Ther	*Dalianraptor*	-1.0297	0.7519	-0.01949		-0.39731	0.1067	0.10416
Ther	*Epidendrosaurus*	-1.0678	0.72498	-0.03994		-0.41055	0.091866	0.083726
Ther	*Mei long*	-1.2433	0.95717	-0.24948		-0.48884	0.14863	0.10501
Ther	*Microraptor gui*	-1.0893	0.89797	0.20133		-0.47756	0.12212	0.097557
Ther	*Microraptor zhaoianus*	-1.1611	0.87452	0.047291		-0.4961	0.1241	0.095057
Ther	*Sinornithoides*	-0.93473	1.2844	0.98987		-0.44543	-0.00429	0.38826
Ther	*Sinornithomimus*	-1.2334	1.3199	0.94617		-0.51228	-0.06699	0.33962
Ther	*Sinosauropteryx*	-1.1523	1.2245	-0.27884		-0.40608	0.25563	-0.03761
Ther	*Sinosauropteryx*	-1.1291	1.2841	-0.22535		-0.41242	0.26443	-0.04155
Ther	*Struthithomimus*	-1.1947	1.421	1.03		-0.53391	-0.05639	0.35251
Ther	*Tyrannosaurus*	-1.1587	1.2133	-0.26892		-0.3904	0.23153	-0.05488
