# Supplementary material for: Assessing Arboreal Adaptations of Bird Antecedents: Testing the Ecological Setting of the Origin of the Avian Flight Stroke
Source: PLoS One. 2011 Aug 9;6(8):e22292. doi: 10.1371/journal.pone.0022292 (PMC3153453; doi:10.1371/journal.pone.0022292)
Supplement: Table S4 — PCO loadings for first 3 axes for total dataset using hindlimb characters only. Percentage of variance explained by the first three axes for Euclidean setting: 42.6, 25.3 and 12.6%. For Correlation setting: 40.5, 18.0 and 8.0%. All other axes explain less than 5% of the variance. (PDF) [file pone.0022292.s017.pdf]

		Euclidean				Correlation		
category	taxon	axis 1	axis 2	axis 3		axis 1	axis 2	axis 3
A	*Aotus trivirgatus*	1.8378	-0.36511	1.6728		0.62904	0.36479	0.39572
A	*Arctictis binturong*	1.9109	0.69406	-1.1535		0.81128	-0.58338	-0.12537
A	*Bradypus tridactylus*	1.5113	0.36734	-1.2735		0.39488	-0.26051	-0.06572
A	*Callithrix jacchus*	2.1645	-0.71307	-0.00891		0.6688	0.14472	-0.16971
A	*Caluromys lanatus*	2.5578	-1.2679	-0.1342		1.1006	0.28817	-0.30836
A	*Cebuella pygmea*	2.3013	-1.073	-0.40804		0.65523	0.16807	-0.1937
A	*Cercopithecus cephus*	1.8134	-0.46857	1.6111		0.59959	0.37482	0.38361
A	*Chamaeleo calyptratus*	2.0721	-1.7355	1.2371		0.80688	0.87296	-0.05516
A	*Daubbentonia madagascarensis*	2.2668	-1.0066	-0.23969		0.7444	0.19448	-0.22068
A	*Dendrolagus insutus*	1.0717	0.86498	-0.59773		0.47172	-0.45717	-0.28572
A	*Erthizon dorsatum*	1.8392	-0.80929	-0.09381		0.69268	0.23122	-0.31633
A	*Galaucomyus sabrinus*	2.1526	1.0908	-0.7324		0.72277	-0.5287	0.095684
A	*Galaucomyus volans*	2.1378	1.016	-0.76514		0.71548	-0.50558	0.072266
A	*Gymnobeleidus leadbeateri*	2.0862	-1.0353	-0.22704		0.57839	0.17236	-0.15549
A	*Lagothix sp.*	2.1052	-0.73493	1.7164		0.7534	0.5076	0.49114
A	*Lemur fuluvus*	1.7873	-0.57762	1.5355		0.56582	0.38328	0.37184
A	*Leontopithecus sp.*	2.1508	-0.77435	-0.04069		0.65773	0.15388	-0.17522
A	*Loris tardigradius*	1.9469	-0.61425	1.4555		0.66928	0.42186	0.46427
A	*Manis tetradactyla*	1.7974	0.7562	-0.56849		0.68843	-0.42659	-0.13309
A	*Otolemur sp.*	1.5992	-0.64673	1.4222		0.69316	0.5285	0.41971
A	*Perodicticus potto*	1.929	-0.68706	1.3965		0.63891	0.42668	0.45507
A	*Petaurista grandis*	2.1357	0.99784	-0.77023		0.72042	-0.49562	0.056332
A	*Potos flavus*	2.2736	-0.74104	1.6621		0.95058	0.5803	0.62229
A	*Saguinus sp.*	2.1589	-0.74265	-0.02173		0.66735	0.15226	-0.17804
A	*Saimiri sciureus*	2.149	-0.78195	-0.04507		0.65587	0.15467	-0.17523
A	*Scurius carolinesis*	2.1424	1.0378	-0.75475		0.71914	-0.51197	0.07737
A	*Tarsius spectrum*	1.3369	-1.3134	-0.41943		0.46502	0.32649	-0.40807
A-Fossil	*Megalancosaurus*	2.4629	-1.6962	-0.49294		0.84754	0.37766	-0.29016
A-Fossil	*Vallesaurus*	2.5687	-2.0499	-0.80808		0.8199	0.41697	-0.22228
A-Fossil	*Sumina*	1.734	-1.0869	0.76274		0.8375	0.65167	0.012737
B-A	*Alcedo atthis*	-1.2447	-1.63	-0.6385		-0.3414	0.27529	-0.31528
B-A	*Ara severus*	-1.1405	-2.451	-0.22245		-0.28121	0.48103	-0.27565
B-A	*Chaetura pelagica*	-1.4333	-2.3303	-1.696		-0.27989	0.24631	-0.16929
B-A	*Coccyzus erythrophthalmus*	-1.1606	-2.5	-0.30506		-0.29016	0.45772	-0.24003
B-A	*Opisthocomus hoazin*	-1.2313	-1.5461	-0.58955		-0.35682	0.25735	-0.30296
B-C	*Certhia familiaris*	-1.2474	-1.5882	-0.65412		-0.35801	0.24247	-0.27186
B-C	*Dryocopus pileatus*	-1.0764	-2.8784	-0.54396		-0.32441	0.53271	-0.26598
B-C	*Melanerpes erythrocephalus*	-1.0146	-2.6384	-0.25974		-0.34025	0.56389	-0.31751
B-C	*Sitta europaea*	-1.2592	-1.6454	-0.7022		-0.35079	0.24808	-0.27081
B-G	*Cinclus cinclus*	-1.3825	-1.3125	0.27845		-0.45388	0.44524	-0.04893
B-G	*Columba livia*	-1.343	-1.1691	0.41337		-0.46992	0.47584	-0.08372

B-G	*Corvus corax*	-1.2437	-1.5943	-0.63759		-0.35311	0.25562	-0.29172
B-G	*Corvus frugilegus*	-1.3887	-1.3444	0.25578		-0.44808	0.44765	-0.05171
B-G	*Crotophaga ani*	-1.2734	-2.1156	0.70756		-0.3707	0.65139	-0.03958
B-G	*Geococcyx sp.*	-1.2683	-2.0889	0.72456		-0.37351	0.64988	-0.037
B-G	*Goura crisata*	-1.3333	-1.107	0.44142		-0.48141	0.46126	-0.06831
B-G	*Melanocorypha calandra*	-1.34	-1.1035	0.42136		-0.48433	0.43298	-0.03808
B-G	*Pica pica*	-1.3853	-1.3215	0.26786		-0.45279	0.44225	-0.04574
B-G	*Sturnus vulgaris*	-1.3809	-1.3106	0.28473		-0.45355	0.44946	-0.05351
B-G	*Turdus_philomelos*	-1.4162	-1.446	0.1449		-0.43283	0.42931	-0.0331
B-GB	*Alectornis sp.*	-1.4423	-0.31882	0.043681		-0.55159	0.16972	-0.12326
B-GB	*Anhima cornuta*	-1.6079	-0.13123	0.88293		-0.62317	0.39032	0.24858
B-GB	*Cariama cristata*	-1.4939	0.78012	-0.16869		-0.60803	-0.24758	-0.12178
B-GB	*Dromaius novaehollandiae*	-1.6492	0.99375	0.70124		-0.68921	-0.04397	0.28209
B-GB	*Gallus gallus*	-1.5934	-0.06595	0.92847		-0.6402	0.3902	0.25577
B-GB	*Meleagris gallopavo*	-1.4431	-0.30207	0.040202		-0.55457	0.15325	-0.1037
B-GB	*Rhea sp.*	-1.64	1.041	0.72376		-0.69247	-0.05752	0.29296
B-GB	*Struthio camelus*	-1.6429	1.019	0.71654		-0.69554	-0.04741	0.28356
BOP	*Bubo virginanus*	-1.3129	-1.8781	-0.95169		-0.32386	0.25646	-0.24477
BOP	*Buteo jamaicensis*	-1.1756	-1.3012	-0.4027		-0.3766	0.24949	-0.33252
BOP	*Falco sparverius*	-1.1873	-1.3531	-0.43823		-0.37334	0.25126	-0.32683
BOP	*Strix varia*	-1.3505	-2.0183	-1.1574		-0.31065	0.25115	-0.21554
Liz	*Anolis sp.*	1.5909	0.69986	-0.95484		0.50576	-0.37024	-0.06997
Liz	*Crotaphytus collaris*	1.6739	1.0453	-0.72646		0.63316	-0.47841	-0.06919
Liz	*Draco sp.*	1.5606	0.53794	-1.0632		0.48141	-0.29847	-0.11724
Liz	*Lacerta agilis*	1.5429	0.4635	-1.1334		0.45544	-0.2744	-0.11389
Liz	*Phrynosome solare*	1.633	0.87309	-0.82607		0.57385	-0.42705	-0.0744
Liz	*Varanus niloticus*	1.6504	0.91943	-0.78097		0.62145	-0.4256	-0.11971
Liz	*Xuanlong zhaoi*	1.5477	0.4995	-1.1146		0.45381	-0.29369	-0.0945
Scan	*Aliurus filgens*	1.9524	0.92719	-0.75168		0.59231	-0.36787	0.010904
Scan	*Chlorocebus pygerythrus*	1.8087	-0.49256	1.5984		0.59676	0.3812	0.37638
Scan	*Didelphis sp.*	2.1276	-1.0801	0.002428		0.96232	0.34331	-0.40817
Scan	*Felis catus*	-0.25107	0.76092	-1.0264		-0.21281	-0.4658	-0.13007
Scan	*Genetta genetta*	0.35283	0.93809	-0.81049		0.064668	-0.47459	-0.19903
Scan	*Gulo gulo*	0.92234	0.85472	-1.0513		0.41429	-0.40549	-0.25285
Scan	*Lemur catta*	1.3596	-0.38653	1.6792		0.53506	0.46679	0.31856
Scan	*Leopardus pardal*	-0.41005	0.49715	-1.5004		-0.23276	-0.44737	-0.14839
Scan	*Leptailurus serval*	-0.40983	0.50603	-1.4996		-0.23489	-0.4513	-0.13888
Scan	*Marmosa mexicana*	1.3597	-1.2019	0.032443		0.50127	0.39784	-0.41324
Scan	*Martes americana*	2.1515	1.0764	-0.73535		0.72966	-0.52039	0.079958
Scan	*Martes pennanti*	2.1404	1.0223	-0.75952		0.72251	-0.50424	0.065263
Scan	*Monodelphis sp.*	1.9681	-1.0413	0.078823		0.73122	0.2926	-0.31466
Scan	*Nasua narica*	1.8165	1.2153	0.14465		0.67332	-0.37739	0.33681
Scan	*Panthera pardus*	-0.40935	0.52678	-1.4977		-0.23909	-0.45932	-0.11663

Scan	*Papio papio*	0.79071	-0.39039	1.735		0.38977	0.64173	0.29599
Scan	*Procavia capensis*	0.13587	1.4024	1.1026		0.24415	-0.16248	0.50862
Scan	*Procyon lotor*	1.8169	1.2325	0.1461		0.66027	-0.38986	0.35823
Scan	*Puma concolor*	-0.40994	0.50159	-1.5		-0.23385	-0.44937	-0.14364
Scan	*Rhampholeon brevicaudatus*	1.7046	-1.7532	0.91284		0.52331	0.61299	-0.00434
Scan	*Tamias minus*	1.8281	1.2945	0.17122		0.66198	-0.41186	0.38347
Scan	*Tupia ferruginea (gillis)*	0.4737	1.1076	-0.55698		0.24165	-0.34803	-0.33551
Terr	*Acinonyx jubatus*	-1.2312	0.72187	-0.50082		-0.50743	-0.34435	0.10139
Terr	*Canis familiaris*	-1.4592	0.93603	-0.08886		-0.62288	-0.28244	-0.11898
Terr	*Cavia porcellus*	0.31774	1.3069	0.29034		0.27543	-0.33481	-0.0023
Terr	*Chinchilla sp.*	-0.26877	1.279	0.33016		-0.02916	-0.28664	-0.0972
Terr	*Dasyprocta sp.*	-1.623	1.1377	0.76267		-0.68494	-0.08949	0.32181
Terr	*Dipodomys ordii*	-0.27882	1.2354	0.30833		-0.0353	-0.27071	-0.09692
Terr	*Equus caballus*	-0.99773	1.3796	1.0172		-0.60801	-0.13021	0.37383
Terr	*Erinaceus europaneus*	0.3045	1.2712	0.26462		0.23547	-0.32116	0.038622
Terr	*Helogale parvula*	-0.3766	1.1308	0.099962		-0.24332	-0.41981	0.043656
Terr	*Hystirx cristata*	0.24022	0.56828	1.4583		0.26883	0.38911	0.34059
Terr	*Lepus americannus*	-1.6313	1.0991	0.74454		-0.68338	-0.08071	0.31483
Terr	*Lynx lynx*	-0.25948	0.71696	-1.0444		-0.21422	-0.455	-0.14123
Terr	*Macropos sp*	-1.6282	1.1304	0.75269		-0.67014	-0.09574	0.33063
Terr	*Marmot monax*	0.76154	1.1688	0.18914		0.42849	-0.3657	0.15212
Terr	*Mephitus mephistus*	0.30858	1.2668	0.27198		0.26231	-0.31748	-0.00652
Terr	*Metachirus sp.*	0.24147	0.58794	1.4616		0.25423	0.35736	0.36212
Terr	*Mustela erminea*	0.46274	1.0612	-0.5793		0.23059	-0.33618	-0.33132
Terr	*Octodon degu*	-0.2757	1.2218	0.31323		-0.02926	-0.27259	-0.16826
Terr	*Odocoileus sp.*	-1.0093	1.3114	0.99624		-0.63308	-0.09361	0.33717
Terr	*Oryctocagus sp.*	-1.6304	1.103	0.74656		-0.68392	-0.08138	0.31526
Terr	*Panthera leo*	-0.40994	0.50159	-1.5		-0.23385	-0.44937	-0.14364
Terr	*Panthera tigris*	-0.40962	0.51492	-1.4988		-0.23682	-0.45493	-0.12935
Terr	*Pecari ta jacu*	-1.0032	1.3587	1.0078		-0.59996	-0.12449	0.37082
Terr	*Rattus sp.*	0.80654	1.3703	0.28162		0.50164	-0.45585	0.20154
Terr	*Spermophilius franklini*	0.78165	1.2572	0.23394		0.46488	-0.40719	0.1709
Terr	*Sus sp.*	-1.0234	1.269	0.96884		-0.58531	-0.09206	0.34597
Terr	*Tapirus sp.*	-1.0135	1.346	0.99047		-0.53752	-0.13387	0.39231
Terr	*Taxidea taxus*	0.59733	1.3167	1.0369		0.45994	-0.22799	0.62953
Terr	*Urogale everetti*	0.45733	1.0407	-0.59084		0.22363	-0.33145	-0.32413
BB	*Archaeopteryx*	-1.5592	0.56238	-0.36477		-0.5376	-0.20452	-0.03389
BB	*Archaeopteryx*	-1.5484	0.61495	-0.32668		-0.54397	-0.21758	-0.02947
BB	*Archaeopteryx*	-1.5375	0.67311	-0.28944		-0.54719	-0.23172	-0.01883
BB	*Confuciusornis*	-1.3629	-1.2574	0.34971		-0.45716	0.47257	-0.07938
BB	*Jeholornis*	-1.3591	-1.1697	0.36041		-0.47586	0.41707	-0.02064
BB	*Pengornis*	-1.229	-1.5473	-0.57994		-0.35388	0.26429	-0.31345
BB	*Sapeornis*	-1.2057	-1.3794	-0.49838		-0.38065	0.21946	-0.26917

BB	*Sinornis*	-1.3689	-1.2748	0.32858		-0.45644	0.46455	-0.07021
Ther	*Allosaurus*	-1.5277	0.62481	-0.2627		-0.58173	-0.20612	-0.12114
Ther	*Anchiornis*	-1.548	0.63859	-0.32341		-0.53543	-0.22382	-0.00658
Ther	*Bambiraptor*	-1.5026	0.80448	-0.18795		-0.57732	-0.26169	-0.04238
Ther	*Caudipteryx*	-1.6754	0.92586	0.63294		-0.64021	-0.05615	0.30331
Ther	*Caudipteryx*	-1.6861	0.88667	0.60168		-0.62688	-0.05186	0.30233
Ther	*Compsognathus*	-1.5316	0.67119	-0.27318		-0.56242	-0.23006	-0.04917
Ther	*Compsognathus*	-1.5319	0.6554	-0.27492		-0.56737	-0.22472	-0.06538
Ther	*Dalianraptor*	-1.552	0.5952	-0.33947		-0.54263	-0.21258	-0.03313
Ther	*Epidendrosaurus*	-1.5605	0.5338	-0.37068		-0.54261	-0.19478	-0.0564
Ther	*Mei long*	-1.7624	0.74989	-0.4663		-0.62784	-0.30399	-0.07155
Ther	*Microraptor gui*	-1.5237	0.69778	-0.24927		-0.57122	-0.23584	-0.05923
Ther	*Microraptor zhaoianus*	-1.5246	0.69409	-0.25196		-0.5705	-0.23497	-0.05888
Ther	*Sinornithoides*	-1.6751	0.94558	0.63526		-0.62852	-0.06809	0.3161
Ther	*Sinornithomimus*	-1.6831	0.87119	0.60941		-0.64518	-0.03477	0.28371
Ther	*Sinosauropteryx*	-1.523	0.68833	-0.24799		-0.57616	-0.23213	-0.07354
Ther	*Sinosauropteryx*	-1.5096	0.74145	-0.20939		-0.58785	-0.24437	-0.08216
Ther	*Struthithomimus*	-1.6659	0.94717	0.65867		-0.66047	-0.05016	0.29385
Ther	*Tyrannosaurus*	-1.5147	0.69384	-0.22456		-0.59137	-0.2275	-0.11141
