# Supplementary material for: Assessing Arboreal Adaptations of Bird Antecedents: Testing the Ecological Setting of the Origin of the Avian Flight Stroke
Source: PLoS One. 2011 Aug 9;6(8):e22292. doi: 10.1371/journal.pone.0022292 (PMC3153453; doi:10.1371/journal.pone.0022292)
Supplement: Table S5 — PCO loadings for first axes for the quadrupedal only dataset. Percentage of variance explained by the first four axes for Euclidean setting: 47.2, 16.9, 10.2 and 5.2% For Correlation setting the first three axes explained: 39.0, 16.1 and 4.9%. All other axes explain less than 5% of the variance. (PDF) [file pone.0022292.s018.pdf]

		Euclidean					correlation		
category	taxon	axis 1	axis 2	axis 3	axis 4		axis 1	axis 2	axis 3
A	*Aotus trivirgatus*	2.3589	-1.4749	0.58659	-1.0127		0.56644	0.38237	-0.1306
A	*Arctictis binturong*	0.90391	1.515	0.066615	0.64325		0.37778	-0.50983	-0.09966
A	*Bradypus tridactylus*	1.2861	1.6036	-1.2543	-0.65386		0.16863	-0.19507	-0.17858
A	*Callithrix jacchus*	2.3307	0.17948	0.038912	-0.14499		0.56987	-0.10369	-0.07753
A	*Caluromys lanatus*	3.1323	-0.46388	-0.39621	1.3579		0.91358	0.005821	-0.05962
A	*Cebuella pygmea*	2.6388	0.42828	-0.39363	-0.28835		0.57658	-0.11185	-0.08332
A	*Cercopithecus cephus*	2.3809	-1.4946	0.4833	-1.0167		0.55021	0.39396	-0.13202
A	*Chamaeleo calyptratus*	3.1677	-2.0946	0.47009	1.6119		0.84296	0.57224	0.10708
A	*Daubbentonia madagascarensis*	3.0554	-0.12682	-1.4017	-0.55719		0.50166	0.1228	-0.1074
A	*Dendrolagus insutus*	0.57244	0.51428	0.010662	0.69385		0.24941	-0.2159	-0.14232
A	*Erthizon dorsatum*	1.5093	0.16843	0.46666	0.53163		0.63577	-0.13614	0.24932
A	*Galaucomyus sabrinus*	1.6677	1.096	-0.1197	-0.67483		0.39414	-0.26271	-0.18547
A	*Galaucomyus volans*	1.7168	1.0941	-0.26514	-0.72078		0.383	-0.24334	-0.20132
A	*Gymnobeleidus leadbeateri*	2.7192	-0.38638	-0.49273	-0.16373		0.52619	0.070613	-0.11122
A	*Lagothix sp.*	2.6857	-1.6917	0.46427	0.44627		0.70932	0.50649	-0.00456
A	*Lemur fuluvus*	2.4048	-1.5429	0.32486	-1.049		0.51399	0.4185	-0.11809
A	*Leontopithecus sp.*	2.3492	0.17322	-0.02632	-0.15063		0.56301	-0.09681	-0.08457
A	*Loris tardigradius*	2.5737	-1.4626	0.18412	-1.0912		0.60184	0.45695	-0.13436
A	*Manis tetradactyla*	0.57649	1.3535	0.62628	1.5143		0.35485	-0.48034	0.16665
A	*Otolemur sp.*	2.2874	-1.542	0.23978	-0.8545		0.56188	0.52068	-0.02536
A	*Perodicticus potto*	2.554	-1.4267	0.21184	-1.0162		0.6168	0.43917	-0.17659
A	*Petaurista grandis*	1.7134	1.134	-0.22164	-0.68094		0.41218	-0.25012	-0.23078
A	*Potos flavus*	2.7991	-1.5617	0.52034	0.50932		0.8781	0.50617	-0.06128
A	*Saguinus sp.*	2.3347	0.17769	0.02904	-0.1385		0.57297	-0.1039	-0.08433
A	*Saimiri sciureus*	2.3477	0.16925	-0.0231	-0.14677		0.56221	-0.09762	-0.08028
A	*Scurius carolinesis*	1.3669	1.2439	0.23124	-0.43954		0.41601	-0.3134	-0.04019
A	*Tarsius spectrum*	2.3508	-0.84167	-1.616	0.57241		0.31197	0.28635	-0.01651
A-Fossil	*Megalancosaurus*	3.5287	-0.96084	-0.99733	1.3755		0.7689	0.2613	-0.17475
A-Fossil	*Vallesaurus*	2.8413	0.84882	-1.4215	1.1571		0.55119	-0.12724	-0.11126
A-Fossil	*Sumina*	2.2808	-1.2363	-0.43292	0.28869		0.69756	0.50001	-0.2179
Liz	*Anolis sp.*	1.0461	1.9639	-1.3543	-0.74077		0.14268	-0.17629	-0.04346
Liz	*Crotaphytus collaris*	0.62737	1.649	0.54607	-0.10933		0.31224	-0.45044	0.042762
Liz	*Draco sp.*	0.99738	1.811	-0.83202	-0.50438		0.18657	-0.22647	-0.06029
Liz	*Lacerta agilis*	0.80037	1.7107	-0.10123	-0.12477		0.275	-0.3289	-0.08829
Liz	*Phrynosome solare*	0.72446	1.6506	0.26991	-0.17267		0.27483	-0.39584	0.013402
Liz	*Varanus niloticus*	0.71496	1.698	0.39967	-0.13895		0.31689	-0.41363	0.005955
Liz	*Xuanlong zhaoi*	0.94749	1.7333	-0.69768	-0.42549		0.18746	-0.23933	-0.06591
Scan	*Aliurus filgens*	1.567	0.62386	0.064716	-0.41491		0.39925	-0.10269	-0.08585
Scan	*Chlorocebus pygerythrus*	2.3904	-1.5277	0.43769	-1.0412		0.54021	0.40853	-0.12185
Scan	*Didelphis sp.*	2.0462	0.043119	0.4089	1.8701		0.82581	-0.20249	0.46226

Scan	*Felis catus*	-1.0038	0.87355	-0.55839	0.24354		-0.40042	-0.43481	-0.11827
Scan	*Genetta genetta*	-0.31282	1.1835	-0.11173	0.006407		-0.06054	-0.37999	-0.09427
Scan	*Gulo gulo*	-0.14063	1.6646	0.16471	-0.0081		0.15616	-0.47358	0.017522
Scan	*Lemur catta*	1.6788	-1.7465	0.74588	-0.55961		0.48317	0.55271	0.002596
Scan	*Leopardus pardal*	-0.97904	1.2323	-1.1722	0.052162		-0.38747	-0.42551	-0.14496
Scan	*Leptailurus serval*	-0.98411	1.2286	-1.1785	0.051331		-0.38805	-0.42602	-0.14872
Scan	*Marmosa mexicana*	1.2936	-0.33339	0.16133	2.06		0.46459	0.052113	0.38017
Scan	*Martes americana*	0.78919	1.7346	0.51542	-0.27192		0.3415	-0.47437	-0.02924
Scan	*Martes pennanti*	0.77295	1.718	0.54628	-0.23328		0.35205	-0.49071	-0.03576
Scan	*Monodelphis sp.*	1.9088	-0.63342	0.54151	2.1128		0.69417	0.11007	0.37007
Scan	*Nasua narica*	0.44884	1.061	1.0447	-0.62156		0.32835	-0.28976	-0.01241
Scan	*Panthera pardus*	-0.99049	1.206	-1.201	0.034391		-0.41106	-0.42383	-0.11915
Scan	*Papio papio*	0.98844	-1.8599	0.86187	-0.34127		0.36879	0.7493	0.1994
Scan	*Procavia capensis*	-1.0931	0.023219	1.5275	-0.40243		0.028393	0.10823	0.20169
Scan	*Procyon lotor*	0.44661	1.0185	1.0009	-0.65533		0.28735	-0.27638	0.016245
Scan	*Puma concolor*	-0.98282	1.2187	-1.1888	0.044603		-0.39153	-0.42845	-0.15525
Scan	*Rhampholeon brevicaudatus*	3.0576	-1.9745	-0.27093	-0.1006		0.58161	0.53731	0.006537
Scan	*Tamias minus*	0.46933	1.032	0.95373	-0.7047		0.26864	-0.25618	0.023322
Scan	*Tupia ferruginea (gillis)*	-0.91807	1.2032	1.0182	0.60746		-0.05501	-0.42169	0.36903
Terr	*Acinonyx jubatus*	-1.6398	0.33401	-0.92984	-0.34896		-0.59496	-0.19767	-0.2435
Terr	*Canis familiaris*	-2.7074	-0.22951	0.12432	0.52555		-0.83019	-0.10858	0.18326
Terr	*Cavia porcellus*	-1.2167	0.51185	1.2863	0.16764		-0.06711	-0.152	0.30081
Terr	*Chinchilla sp.*	-1.6608	0.18767	1.0833	0.22004		-0.29487	-0.07675	0.32084
Terr	*Dasyprocta sp.*	-2.8649	-0.77821	0.49042	0.10511		-0.83946	0.20755	0.093851
Terr	*Dipodomys ordii*	-1.4781	0.33595	1.1053	0.20466		-0.2976	-0.1412	0.37807
Terr	*Equus caballus*	-2.5471	-0.52979	1.2991	0.14323		-0.75904	0.19125	0.25704
Terr	*Erinaceus europaneus*	-1.0604	0.65651	1.3411	0.18128		-0.07321	-0.24895	0.38607
Terr	*Helogale parvula*	-1.2313	0.32953	0.38682	-0.07836		-0.33899	-0.26992	-0.24121
Terr	*Hystirx cristata*	-0.50182	-0.8368	1.6472	0.027309		0.21092	0.47896	0.5377
Terr	*Lepus americannus*	-2.8031	-0.7948	0.34848	0.032551		-0.80475	0.21072	0.090255
Terr	*Lynx lynx*	-1.2719	0.9533	-0.29602	0.39439		-0.45952	-0.4567	0.10408
Terr	*Macropos sp*	-2.3929	-0.54623	0.3371	-0.12562		-0.73736	0.10879	0.16219
Terr	*Marmot monax*	-0.33432	0.71848	0.86442	-0.27612		0.13851	-0.24288	-0.04844
Terr	*Mephitus mephistus*	-1.0291	0.67699	1.315	0.15824		-0.03878	-0.24496	0.3452
Terr	*Metachirus sp.*	-0.39161	-1.6179	1.4299	0.019153		0.12891	0.75889	0.43968
Terr	*Mustela erminea*	-0.8577	1.2539	0.91385	0.58317		-0.01955	-0.38561	0.29424
Terr	*Octodon degu*	-1.1695	0.34076	0.85988	0.088494		-0.14948	-0.13757	0.069086
Terr	*Odocoileus sp.*	-2.505	-0.55034	1.2153	0.11302		-0.77931	0.2104	0.20072
Terr	*Oryctocagus sp.*	-2.8432	-0.77967	0.44004	0.091378		-0.82219	0.20832	0.086163
Terr	*Panthera leo*	-0.98226	1.22	-1.188	0.045324		-0.38986	-0.43028	-0.16089
Terr	*Panthera tigris*	-0.98729	1.2136	-1.1955	0.041015		-0.39742	-0.4285	-0.14883
Terr	*Pecari ta jacu*	-2.5311	-0.51171	1.271	0.14887		-0.76917	0.19325	0.17786

Terr	*Rattus sp.*	-0.10599	0.82886	0.92057	-0.45701		0.1853	-0.22695	0.002576
Terr	*Spermophilius franklini*	-0.36672	0.73373	0.97408	-0.25918		0.14664	-0.26542	-0.009
Terr	*Sus sp.*	-2.4115	-0.5648	0.98363	0.00594		-0.6739	0.2227	0.1414
Terr	*Tapirus sp.*	-2.5062	-0.55717	1.1669	0.095844		-0.70329	0.19024	0.24351
Terr	*Taxidea taxus*	-0.42898	0.17644	1.1659	-0.7477		0.15819	0.008576	-0.11327
Terr	*Urogale everetti*	-0.85947	1.2032	0.87638	0.56022		-0.06732	-0.3804	0.33613
BB	*Archaeopteryx*	-1.6974	-0.88507	-1.1311	0.047803		-0.57421	0.19891	-0.08413
BB	*Archaeopteryx*	-1.6889	-0.90017	-1.1457	0.008097		-0.57767	0.19719	-0.06807
BB	*Archaeopteryx*	-1.7187	-0.92814	-1.0631	0.016454		-0.60286	0.19291	-0.03594
Ther	*Allosaurus*	-1.6651	-0.7766	-1.0069	0.060078		-0.49533	0.20729	-0.18523
Ther	*Anchiornis*	-1.6943	-0.93979	-1.1969	-0.02511		-0.58112	0.19938	-0.04283
Ther	*Bambiraptor*	-1.7568	-0.90788	-0.87922	0.03982		-0.60472	0.19002	-0.0457
Ther	*Caudipteryx*	-1.8441	-1.3884	-0.63228	-0.41056		-0.54243	0.45235	-0.17837
Ther	*Caudipteryx*	-1.8486	-1.4182	-0.67107	-0.41068		-0.5527	0.45396	-0.16538
Ther	*Compsognathus*	-2.0209	-0.26639	-0.82242	0.081774		-0.56919	-0.0247	-0.11366
Ther	*Compsognathus*	-2.0004	-0.2568	-0.85097	0.065954		-0.56179	-0.02105	-0.12423
Ther	*Dalianraptor*	-1.6385	-0.84317	-1.27	-0.03341		-0.53548	0.19727	-0.09521
Ther	*Epidendrosaurus*	-1.6516	-0.84132	-1.2038	0.019897		-0.54075	0.20373	-0.12316
Ther	*Mei long*	-1.9546	-0.76264	-1.256	-0.11076		-0.6377	0.19233	-0.01756
Ther	*Microraptor gui*	-1.7666	-0.94271	-0.91318	0.067358		-0.61574	0.20308	-0.05644
Ther	*Microraptor zhaoianus*	-1.8052	-0.85629	-1.2531	-0.14661		-0.6338	0.20541	-0.04118
Ther	*Sinornithoides*	-1.8701	-1.4242	-0.60774	-0.40357		-0.56186	0.45322	-0.15591
Ther	*Sinornithomimus*	-2.1164	-1.4403	-0.45799	-0.18471		-0.59938	0.51338	-0.23926
Ther	*Sinosauropteryx*	-2.0547	-0.26004	-0.69655	0.13103		-0.57893	-0.0347	-0.1218
Ther	*Sinosauropteryx*	-2.0729	-0.25587	-0.61786	0.14214		-0.58763	-0.04291	-0.12388
Ther	*Struthithomimus*	-2.1546	-1.4435	-0.32352	-0.1556		-0.62549	0.51803	-0.22652
Ther	*Tyrannosaurus*	-2.0391	-0.23805	-0.67195	0.12796		-0.553	-0.02678	-0.18162
