# Supplementary material for: Assessing Arboreal Adaptations of Bird Antecedents: Testing the Ecological Setting of the Origin of the Avian Flight Stroke
Source: PLoS One. 2011 Aug 9;6(8):e22292. doi: 10.1371/journal.pone.0022292 (PMC3153453; doi:10.1371/journal.pone.0022292)
Supplement: Table S6 — PCO loadings for first 3 axes for the quadrupedal only dataset using hindlimb characters only. Percentage of variance explained by the first four axes for Euclidean setting: 50.7, 16.7, 10.5 and 6.4% For Correlation setting the first three axes explained: 47.5, 14.9 and 5.3%. All other axes explain less than 5% of the variance. (PDF) [file pone.0022292.s019.pdf]

		Euclidean					Correlation		
category	taxon	axis 1	axis 2	axis 3	axis 4		axis 1	axis 2	axis 3
A	*Aotus trivirgatus*	1.6172	1.5545	0.48415	-0.89753		0.57676	0.44822	-0.2775
A	*Arctictis binturong*	1.1948	-1.3044	0.12755	0.85185		0.49083	-0.50326	0.094656
A	*Bradypus tridactylus*	0.97267	-1.3107	-0.14754	-0.10152		0.22213	-0.22441	0.053158
A	*Callithrix jacchus*	2.0106	0.04	-0.17403	-0.53445		0.59325	-0.0688	-0.07578
A	*Caluromys lanatus*	2.5693	0.16995	-1.1515	0.87151		1.0544	-0.15275	0.094362
A	*Cebuella pygmea*	2.2322	-0.09845	-0.42287	-0.64352		0.58551	-0.06009	-0.04435
A	*Cercopithecus cephus*	1.6321	1.5543	0.37598	-0.9143		0.55522	0.45614	-0.24262
A	*Chamaeleo calyptratus*	2.3445	1.596	-0.85032	1.3067		0.94377	0.49979	0.38138
A	*Daubbentonia madagascarensis*	2.2134	-0.04679	-0.50612	-0.56041		0.69125	-0.09103	-0.0498
A	*Dendrolagus insutus*	0.37088	-0.8261	0.22345	0.93512		0.20247	-0.45837	0.18596
A	*Erthizon dorsatum*	1.7589	0.01	-0.20361	0.071854		0.66946	-0.10534	0.13215
A	*Galaucomyus sabrinus*	1.2951	-1.2435	0.61476	-0.46261		0.40032	-0.34159	-0.2542
A	*Galaucomyus volans*	1.3098	-1.2367	0.55346	-0.46872		0.40126	-0.33927	-0.24176
A	*Gymnobeleidus leadbeateri*	2.0539	0.02	-0.53642	-0.60434		0.52379	-0.02186	-0.00174
A	*Lagothix sp.*	1.9784	1.8053	-0.30851	0.47146		0.73201	0.62127	-0.05761
A	*Lemur fuluvus*	1.6376	1.5709	0.22269	-0.97335		0.50735	0.48	-0.19293
A	*Leontopithecus sp.*	2.0212	0.04	-0.23008	-0.53914		0.58798	-0.0648	-0.06278
A	*Loris tardigradius*	1.8057	1.4696	0.16942	-0.93158		0.63121	0.52345	-0.2637
A	*Manis tetradactyla*	1.0872	-0.86615	0.043711	1.495		0.43553	-0.40274	0.17507
A	*Otolemur sp.*	1.4745	1.5556	0.32917	-0.43632		0.64564	0.60202	-0.08973
A	*Perodicticus potto*	1.8225	1.4364	0.10355	-0.92869		0.62445	0.51574	-0.24349
A	*Petaurista grandis*	1.3232	-1.2553	0.57025	-0.44236		0.42644	-0.35635	-0.24428
A	*Potos flavus*	2.151	1.6913	-0.29755	0.54584		0.9542	0.66257	-0.14183
A	*Saguinus sp.*	2.0176	0.04	-0.19465	-0.53414		0.59606	-0.07015	-0.0715
A	*Saimiri sciureus*	2.0227	0.04	-0.23683	-0.539		0.58729	-0.0644	-0.06127
A	*Scurius carolinesis*	1.3158	-1.2649	0.60526	-0.43116		0.42465	-0.35627	-0.24959
A	*Tarsius spectrum*	1.4159	0.22422	-1.596	0.15861		0.32619	0.094351	0.38544
A-Fossil	*Megalancosaurus*	2.6422	0.06	-1.6414	0.73264		0.85548	-0.03567	0.21597
A-Fossil	*Vallesaurus*	2.8243	-0.05565	-2.0774	0.59732		0.82682	0.037583	0.30645
A-Fossil	*Sumina*	1.7498	1.0329	-0.58374	0.12021		0.91175	0.35604	0.006613
Liz	*Anolis sp.*	0.93171	-1.2127	0.30397	0.055966		0.28507	-0.30825	0.013859
Liz	*Crotaphytus collaris*	0.88083	-1.1852	0.67493	0.11648		0.35211	-0.38986	-0.0462
Liz	*Draco sp.*	0.97124	-1.25	0.16934	0.032977		0.29876	-0.30054	0.029782
Liz	*Lacerta agilis*	0.98949	-1.2938	0.10083	0.031472		0.29695	-0.2876	0.03436
Liz	*Phrynosome solare*	0.91234	-1.2004	0.51962	0.11542		0.3324	-0.35967	-0.0145
Liz	*Varanus niloticus*	0.9228	-1.2151	0.62845	0.15955		0.39153	-0.40677	-0.03192
Liz	*Xuanlong zhaoi*	0.96032	-1.2417	0.059829	-0.03045		0.25687	-0.2668	0.039188
Scan	*Aliurus filgens*	1.183	-1.1817	0.55166	-0.46483		0.35417	-0.27581	-0.16752
Scan	*Chlorocebus pygerythrus*	1.6319	1.5676	0.33915	-0.93568		0.54271	0.46541	-0.23003

Scan	*Didelphis sp.*	2.1013	0.25997	-0.90923	1.4492		0.94766	-0.10339	0.42637
Scan	*Felis catus*	-0.80011	-0.99592	-0.61501	-0.49532		-0.40354	-0.37687	-0.16295
Scan	*Genetta genetta*	-0.30307	-0.99366	0.049787	-0.10511		-0.15165	-0.43082	-0.06967
Scan	*Gulo gulo*	0.2445	-1.1763	0.51245	0.28909		0.17246	-0.40552	0.046404
Scan	*Lemur catta*	1.1725	1.6608	0.48043	-0.38974		0.47977	0.54865	-0.01161
Scan	*Leopardus pardal*	-0.83709	-1.212	-0.85259	-0.51214		-0.39387	-0.38342	-0.14368
Scan	*Leptailurus serval*	-0.84262	-1.2088	-0.85789	-0.51825		-0.39988	-0.37863	-0.14515
Scan	*Marmosa mexicana*	1.4277	0.48963	-1.0952	1.5652		0.52062	0.067238	0.57925
Scan	*Martes americana*	1.3136	-1.2768	0.65428	-0.41544		0.43783	-0.367	-0.25986
Scan	*Martes pennanti*	1.3244	-1.2737	0.61072	-0.4175		0.4382	-0.36505	-0.25076
Scan	*Monodelphis sp.*	1.9394	0.33843	-0.8354	1.4297		0.71307	-0.03302	0.35107
Scan	*Nasua narica*	0.96569	-0.42263	0.97389	-0.64917		0.39327	-0.09674	-0.33608
Scan	*Panthera pardus*	-0.85633	-1.1996	-0.87272	-0.53534		-0.41431	-0.3659	-0.14967
Scan	*Papio papio*	0.64856	1.7986	0.42059	-0.2006		0.37319	0.73218	0.071994
Scan	*Procavia capensis*	-0.63451	0.63298	1.3841	0.47735		0.013283	0.29226	0.038099
Scan	*Procyon lotor*	0.94939	-0.40202	0.94561	-0.68062		0.35704	-0.07441	-0.32733
Scan	*Puma concolor*	-0.84315	-1.2018	-0.86629	-0.52704		-0.40403	-0.37851	-0.14775
Scan	*Rhampholeon brevicaudatus*	2.0118	1.3514	-0.57535	-0.2085		0.60327	0.4239	0.21651
Scan	*Tamias minus*	0.9332	-0.40106	0.98359	-0.68508		0.34557	-0.06959	-0.33537
Scan	*Tupia ferruginea (gillis)*	-0.26239	-0.85904	0.73128	0.80157		0.006333	-0.36505	0.27309
Terr	*Acinonyx jubatus*	-1.6586	-0.21181	-0.58078	-0.53474		-0.6607	-0.1031	-0.35666
Terr	*Canis familiaris*	-1.9633	-0.00318	-0.27564	0.06514		-0.76735	-0.11739	0.10564
Terr	*Cavia porcellus*	-0.45403	-0.12834	1.1049	0.64647		0.021588	-0.14949	0.15605
Terr	*Chinchilla sp.*	-1.0083	0.07	0.9318	0.76855		-0.26663	-0.07257	0.25649
Terr	*Dasyprocta sp.*	-2.1723	0.75537	0.064563	-0.11004		-0.80411	0.33581	-0.09675
Terr	*Dipodomys ordii*	-1.0091	0.09	0.8666	0.73831		-0.273	-0.0363	0.25346
Terr	*Equus caballus*	-1.675	0.74539	0.82024	0.29022		-0.75286	0.34034	-0.08432
Terr	*Erinaceus europaneus*	-0.45961	-0.11457	1.036	0.62676		-0.01712	-0.10512	0.15322
Terr	*Helogale parvula*	-1.0302	-0.09504	0.33959	-0.09507		-0.4455	-0.21525	-0.31509
Terr	*Hystirx cristata*	-0.21593	1.233	1.005	0.44065		0.20135	0.61965	0.2763
Terr	*Lepus americannus*	-2.1687	0.76682	0.019787	-0.12477		-0.80249	0.34311	-0.09028
Terr	*Lynx lynx*	-0.79345	-0.98489	-0.65709	-0.50698		-0.40706	-0.37326	-0.16096
Terr	*Macropos sp*	-2.187	0.78446	0.0067	-0.15148		-0.79725	0.3471	-0.09448
Terr	*Marmot monax*	0.00604	-0.20536	0.9074	0.077941		0.16136	-0.12559	-0.11758
Terr	*Mephitus mephistus*	-0.45028	-0.1173	1.0595	0.63466		0.008454	-0.13273	0.16194
Terr	*Metachirus sp.*	-0.23391	1.2578	0.97452	0.40335		0.14219	0.62782	0.26353
Terr	*Mustela erminea*	-0.24518	-0.8816	0.72148	0.83549		0.024126	-0.37535	0.28549
Terr	*Octodon degu*	-0.9761	0.04	0.95238	0.81895		-0.23978	-0.14838	0.30275
Terr	*Odocoileus sp.*	-1.6605	0.76106	0.7709	0.28247		-0.76253	0.35917	-0.07053
Terr	*Oryctocagus sp.*	-2.1661	0.75827	0.034035	-0.11308		-0.80081	0.33876	-0.08959
Terr	*Panthera leo*	-0.84238	-1.2038	-0.86369	-0.52425		-0.40239	-0.37916	-0.14696
Terr	*Panthera tigris*	-0.84971	-1.2017	-0.86834	-0.52995		-0.40887	-0.37239	-0.14834

Terr	*Pecari ta jacu*	-1.6697	0.74157	0.80885	0.30002		-0.74077	0.33529	-0.07196
Terr	*Rattus sp.*	-0.02892	-0.22093	1.0836	0.085472		0.1877	-0.15972	-0.17262
Terr	*Spermophilius franklini*	-0.00764	-0.21174	0.99254	0.088021		0.17901	-0.14517	-0.13986
Terr	*Sus sp.*	-1.6623	0.77104	0.70487	0.27006		-0.72058	0.36033	-0.04363
Terr	*Tapirus sp.*	-1.6834	0.75764	0.74788	0.27741		-0.68946	0.34	-0.049
Terr	*Taxidea taxus*	-0.174	0.54997	1.263	-0.08205		0.20255	0.23729	-0.32933
Terr	*Urogale everetti*	-0.25114	-0.85893	0.67148	0.80379		0.001393	-0.3512	0.28086
BB	*Archaeopteryx*	-1.9403	0	-0.78647	-0.07551		-0.67655	0.000285	0.13201
BB	*Archaeopteryx*	-1.9549	0.02	-0.74655	-0.07763		-0.68889	0.001728	0.11864
BB	*Archaeopteryx*	-1.9702	0.04	-0.70179	-0.0749		-0.69709	0.002438	0.1058
Ther	*Allosaurus*	-1.9138	0.00568	-0.5851	0.009638		-0.71094	-0.05654	0.14776
Ther	*Anchiornis*	-1.9763	0.05	-0.7778	-0.11459		-0.68585	0.026361	0.10268
Ther	*Bambiraptor*	-1.9854	0.06	-0.54101	-0.04469		-0.73062	-0.01964	0.086917
Ther	*Caudipteryx*	-2.1606	0.7969	-0.21682	-0.2002		-0.75808	0.36419	-0.04827
Ther	*Caudipteryx*	-2.1621	0.80573	-0.28372	-0.22899		-0.7444	0.37023	-0.03861
Ther	*Compsognathus*	-1.9443	0	-0.6194	-0.00852		-0.70005	-0.03831	0.1283
Ther	*Compsognathus*	-1.9361	-0.00021	-0.61608	-0.00396		-0.70247	-0.0436	0.13396
Ther	*Dalianraptor*	-1.9442	0	-0.74473	-0.06		-0.68248	-0.00754	0.12889
Ther	*Epidendrosaurus*	-1.9228	-0.01087	-0.77319	-0.05569		-0.67434	-0.01139	0.14522
Ther	*Mei long*	-2.1909	-0.03336	-0.6185	-0.05266		-0.77466	-0.03492	0.03443
Ther	*Microraptor gui*	-1.9688	0.0693	-0.65185	-0.07894		-0.71591	0.002267	0.10002
Ther	*Microraptor zhaoianus*	-1.9705	0.07	-0.66239	-0.08705		-0.71406	0.0083	0.098667
Ther	*Sinornithoides*	-2.1754	0.81104	-0.2346	-0.22124		-0.75086	0.36564	-0.04991
Ther	*Sinornithomimus*	-2.135	0.76934	-0.21842	-0.17045		-0.75364	0.36046	-0.03613
Ther	*Sinosauropteryx*	-1.9391	0	-0.57259	0.006336		-0.71296	-0.05288	0.13043
Ther	*Sinosauropteryx*	-1.945	0.01	-0.50796	0.020875		-0.72712	-0.06638	0.12439
Ther	*Struthithomimus*	-2.1469	0.77417	-0.13829	-0.15435		-0.77275	0.35565	-0.05463
Ther	*Tyrannosaurus*	-1.9281	0.01	-0.52432	0.023031		-0.72504	-0.0693	0.13695
