# Supplementary material for: Assessing Arboreal Adaptations of Bird Antecedents: Testing the Ecological Setting of the Origin of the Avian Flight Stroke
Source: PLoS One. 2011 Aug 9;6(8):e22292. doi: 10.1371/journal.pone.0022292 (PMC3153453; doi:10.1371/journal.pone.0022292)
Supplement: Table S7 — PCO loadings for first 3 axes for bipedal (avian) only dataset. Percentage of variance explained by the first three axes for Euclidean setting: 68.0, 14.1 and 7.9%. For Correlation setting: 60.4, 10.5 and 6.7%. All other axes explain less than 5% of the variance. (PDF) [file pone.0022292.s020.pdf]

		Euclidean				Correlation		
category	taxon	axis 1	axis 2	axis 3		axis 1	axis 2	axis 3
A	*Ara macao*	1.23280	0.04016	0.46517		0.35189	0.13323	0.03078
A	*Chaetura pelagica*	1.35500	-0.16292	0.11525		0.51657	0.13716	0.02280
A	*Opisthocomus hoazin*	2.35690	-1.79070	-0.87338		0.28675	0.12330	-0.33742
A	*Alcedo atthis*	0.92095	0.06414	-0.72338		0.26206	-0.16176	-0.14133
A	*Coccyzus erythrophthalmus*	0.67694	0.49809	-0.24135		0.21104	-0.10041	-0.03967
BOP	*Bubo virginanus*	1.62300	-0.73741	-0.19413		0.25741	0.08912	-0.10320
BOP	*Buteo jamaicensis*	0.77376	0.47820	0.84327		0.25635	0.05348	0.25760
BOP	*Falco sparverius*	0.85297	0.40543	0.77703		0.27566	0.07284	0.20694
BOP	*Strix varia*	1.78290	-1.01970	-0.36373		0.20178	0.07496	-0.14533
C	*Certhia familiaris*	1.23740	-0.22091	0.42038		0.32408	0.12247	0.04270
C	*Dryocopus pileatus*	1.62300	-0.72694	-0.16281		0.49297	0.14689	-0.13019
C	*Melanerpes erythrocephalus*	1.43970	-0.53037	0.00121		0.49854	0.14103	-0.03393
C	*Sitta europaea*	1.20060	-0.06985	0.44475		0.22688	0.08938	0.02447
G	*Cinclus cinclus*	0.24323	-0.44713	0.71144		0.04962	0.18189	0.06208
G	*Columba livia*	0.52149	0.81438	-0.06598		0.34224	-0.13759	0.04910
G	*Corvus corax*	1.10680	0.02522	0.53239		0.21391	0.08453	0.05694
G	*Corvus frugilegus*	0.63295	0.48943	-0.21589		0.16007	-0.10942	-0.02052
G	*Crotophaga ani*	0.75308	0.37073	-0.54761		0.38738	-0.22363	-0.05464
G	*Geococcyx sp.*	0.64160	0.39661	-0.47226		0.25615	-0.25388	0.01226
G	*Goura crisata*	0.40054	0.86616	0.01532		0.23796	-0.17721	0.11725
G	*Melanocorypha calandra*	0.38446	0.82380	0.00332		0.17635	-0.19020	0.11975
G	*Pica pica*	0.59469	0.51385	-0.19022		0.14132	-0.11985	-0.00361
G	*Sturnus vulgaris*	0.61522	0.54809	-0.19207		0.18757	-0.11213	-0.01292
G	*Turdus philomelos*	0.74201	0.30751	-0.33189		0.13195	-0.09692	-0.05969
GB	*Alectoris rufa*	-0.23882	0.16878	0.01123		0.05178	0.03015	-0.02302
GB	*Anhima cornuta*	-0.33279	0.10726	0.05199		-0.06310	-0.04429	0.03509
GB	*Cariama cristata*	-1.58210	-0.20203	0.49758		-0.57682	0.01062	0.29600
GB	*Dromaius novaehollandiae*	-1.80230	0.24389	-0.40752		-0.60480	-0.19590	0.01567
GB	*Gallus_gallus*	-0.42282	0.20715	0.12957		-0.08665	-0.06884	0.09268
GB	*Meleagris gallopavo*	-0.33963	0.14300	0.06201		-0.05206	-0.03906	0.03681
GB	*Rhea sp.*	-1.82600	0.30989	-0.37968		-0.62387	-0.20612	0.04025
GB	*Struthio camelus*	-1.79140	0.29149	-0.40162		-0.59566	-0.19112	0.00409
BB	*Confuciusornis*	0.57638	0.67842	-0.13388		0.29778	0.09639	0.13674
BB	*Jeholornis*	0.50485	0.67771	-0.08923		0.26355	-0.11959	0.00910
BB	*Pengornis*	0.66438	0.51805	-0.23056		0.13725	-0.14010	0.04006
BB	*Sapeornis*	0.97944	0.27950	0.67005		0.18215	-0.13784	0.04306
BB	*Sinornis*	0.50600	0.62107	-0.10650		0.21427	-0.10360	-0.03440

BB	*Archaeopteryx*	-1.01350	-0.61204	0.06332		-0.25578	0.13770	-0.09191
BB	*Archaeopteryx*	-1.10130	-0.54455	0.13111		-0.29643	0.11680	-0.04108
BB	*Archaeopteryx*	-1.17860	-0.48051	0.19602		-0.33850	0.10378	0.00851
Ther	*Anchiornis*	-1.10580	-0.55166	0.13731		-0.30332	0.11465	-0.03753
Ther	*Bambiraptor*	-1.24230	-0.23897	0.30042		-0.32359	0.17289	0.05998
Ther	*Caudipteryx*	-1.56260	-0.00248	-0.60749		-0.43711	-0.10586	-0.19539
Ther	*Caudipteryx*	-1.60480	0.07074	-0.56424		-0.45785	-0.11717	-0.16994
Ther	*Compsognathus*	-1.01650	-0.40070	0.10749		-0.18408	0.23837	-0.10943
Ther	*Dalianraptor*	-1.10140	-0.56566	0.12137		-0.30223	0.10924	-0.04020
Ther	*Mei long*	-1.27470	-0.32150	0.29722		-0.37770	0.11560	0.08176
Ther	*Microraptor gui*	-1.20610	-0.38404	0.24098		-0.33861	0.12759	0.02878
Ther	*Microraptor zhaoianus*	-1.21000	-0.38601	0.23844		-0.33924	0.12204	0.03051
Ther	*Sinornithoides*	-1.20720	-0.31553	0.25140		-0.31199	0.14996	0.02759
Ther	*Sinosauropteryx*	-1.20870	-0.26237	0.25993		-0.28225	0.16581	0.02502
Ther	*Struthithomimus*	-1.57380	0.01523	-0.60155		-0.44166	-0.10936	-0.18900
