# Supplementary material for: Assessing Arboreal Adaptations of Bird Antecedents: Testing the Ecological Setting of the Origin of the Avian Flight Stroke
Source: PLoS One. 2011 Aug 9;6(8):e22292. doi: 10.1371/journal.pone.0022292 (PMC3153453; doi:10.1371/journal.pone.0022292)
Supplement: Table S8 — Forelimb measurements and BI for theropods and basal birds. (PDF) [file pone.0022292.s021.pdf]

category	taxon	H	U	BI	Ref
BB	*Archaeopteryx*	75	67.5	0.9	[35]
BB	*Archaeopteryx*	63	55	0.87	[35]
BB	*Archaeopteryx*	41.5	36.5	0.88	[35]
BB	*Archaeopteryx*	72	62	0.86	[35]
BB	*Archaeopteryx*	55	53	0.96	[35]
BB	*Archaeopteryx*	83	74	0.89	[35]
BB	*Archaeopteryx*	56.9	50.9	0.89	[35]
BB	*Changchengornis*	33.5	32	0.95	[36]
BB	*Confuciusornis*	51	46	0.9	[36]
BB	*Confuciusornis*	62	61	0.98	Pers. obs
BB	*Confuciusornis*	42	35	0.83	Pers. obs
BB	*Confuciusornis*	51	42	0.82	[36]
BB	*Dalianraptor*	52	46	0.88	[28]
BB	*Jeholornis*	100	109	1.09	[37]
BB	*Jixiangornis*	112	107	0.96	[38]
BB	*Patagopteryx*	65.4	53	0.81	[39]
BB	*Pengornis*	64.3	70.7	1.1	[22]
BB	*Sapeornis*	122.6	124	1.01	[40]
BB	*Shenzhouraptor*	79.8	83.4	1.05	[41]
BB	*Sinornis*	24	22	0.92	[23]
BB	*Yixianornis*	49.3	50.3	1.02	[42]
Ther	*Acrocanthosaurus*	370	255	0.69	Pers. com. Currie
Ther	*Albertosaurus*	205	125	0.61	[43]
Ther	*Allosaurus*	310	263	0.85	[24]
Ther	*Alxasaurus*	375	245	0.65	[44]
Ther	*Anchiornis*	69	55.1	0.8	[25]
Ther	*Bambiraptor*	105	93	0.89	[26]
Ther	*Baryonyx*	463	283	0.61	[45]
Ther	*Buetreraptor*	135	110	0.81	[46]

Ther	*Carnotaurus*	285	78	0.27	[47]
Ther	*Caudipteryx*	73	61	0.84	[48]
Ther	*Caudipteryx*	69	61	0.88	[49]
Ther	*Caudipteryx*	72	62	0.86	[49]
Ther	*Citipati*	235	245	1.04	Pers. com Balanoff
Ther	*Coelurus*	119	96	0.81	[50]
Ther	*Coleophysis*	120	65	0.54	[51]
Ther	*Coleophysis*	134	82	0.61	[51]
Ther	*Compsognathus*	38	28.6	0.75	[27]
Ther	*Compsognathus*	56.3	46.4	0.82	[27]
Ther	*Daspletosaurus*	357	214	0.6	[43]
Ther	*Deinonychus*	237	186	0.78	[52]
Ther	*Deinonychus*	254	208	0.82	[53]
Ther	*Dilophosaurus*	285	209	0.73	[54]
Ther	*Epidendrosaurus*	17.1	15	0.88	[55]
Ther	*Epidendrosaurus*	18.5	15.8	0.85	[55]
Ther	*Epidexipteryx*	50	42	0.84	[55]
Ther	*Falcarius*	255	197	0.77	[56]
Ther	*Fukuiraptor*	242	211	0.87	[57]
Ther	*Gallimimus*	530	375	0.71	[58]
Ther	*Gigantoraptor*	700	560	0.8	Pers.com. Xu
Ther	*Gracilliraptor*	106	91	0.86	[59]
Ther	*Guanlong*	222	178	0.8	[60]
Ther	*Harpymimus*	294	242	0.82	[61]
Ther	*Herrerasaurus*	175	153	0.87	[62]
Ther	*Heyuannia*	130	117	0.9	[63]
Ther	*Huxiagnathus*	88	54.8	0.62	[64]
Ther	*Jinfengopteryx*	49.2	43.3	0.88	[65]
Ther	*Juravenator*	27	19.5	0.72	[66]
Ther	*Khaan*	115	110	0.96	Pers. com. Balanoff
Ther	*Limusaurus*	80	40	0.5	[67]

Ther	*Mahakala*	40	40	1	[68]
Ther	*Mei Long*	42	42	1	[30]
Ther	*Microraptor*	62.1	53.8	0.87	[31]
Ther	*Microraptor*	42	37	0.88	Pers. com Xu
Ther	*Microraptor*	62.9	53.5	0.85	[31]
Ther	*Microraptor gui*	82	73	0.89	Pers. obs.
Ther	*Mononykus*	36.7	34.4	0.94	[69]
Ther	*Nothronychus*	418	302	0.72	[70]
Ther	*Ornithomimus*	276	206	0.75	[34]
Ther	*Oviraptor*	185	147	0.79	[71]
Ther	*Poekilopleuron*	310	180	0.58	[72]
Ther	*Protoarchaeopteryx*	88	74	0.84	[73]
Ther	*Raptorex*	99	57	0.58	[60]
Ther	*Segnosaurus*	560	390	0.7	[74]
Ther	*Sinornithoides*	83.2	65	0.78	[32]
Ther	*Sinornithomimus*	212	147	0.69	[33]
Ther	*Sinornithosaurus*	134	110	0.82	[46]
Ther	*Sinosauropteryx*	20.3	16.9	0.83	Pers. com. Currie
Ther	*Sinosauropteryx*	35.5	28.2	0.79	Pers. com. Currie
Ther	*Sinovenator*	71	59	0.83	[46]
Ther	*Struthithomimus*	310	246	0.79	[34]
Ther	*Szechuanosaurus*	360	240	0.67	[75]
Ther	*Tanycolagreus*	198	152	0.77	[76]
Ther	*Tianyuraptor*	129	103	0.8	Pers com. Sullivan
Ther	*Torvosaurus*	424	220	0.52	[77]
Ther	*Tyrannosaurus*	385	214	0.56	Pers. com. Currie
Ther	*Yixianosaurus*	89	64	0.72	[78]
