# Supplementary material for: Assessing Arboreal Adaptations of Bird Antecedents: Testing the Ecological Setting of the Origin of the Avian Flight Stroke
Source: PLoS One. 2011 Aug 9;6(8):e22292. doi: 10.1371/journal.pone.0022292 (PMC3153453; doi:10.1371/journal.pone.0022292)
Supplement: Table S9 — Hindlimb measurements and CI for theropods and basal birds. (PDF) [file pone.0022292.s022.pdf]

category	taxon	F	T	CI	Ref
BB	*Archaeopteryx*	52.5	71	1.35	[35]
BB	*Archaeopteryx*	37	52.5	1.42	[35]
BB	*Archaeopteryx*	60.5	80.5	1.33	[35]
BB	*Archaeopteryx*	48	71	1.48	[35]
BB	*Archaeopteryx*	70	89.5	1.28	[35]
BB	*Archaeopteryx*	50.3	74.6	1.48	[35]
BB	*Changchengornis*	33.46	36.59	1.09	[36]
BB	*Confuciusornis*	33	41	1.24	[36]
BB	*Confuciusornis*	41.78	48.7	1.17	[36]
BB	*Confuciusornis*	46.85	53.29	1.14	[36]
BB	*Dalianraptor*	49	68	1.39	[28]
BB	*GMV-2156*	14.5	17.1	1.18	[79]
BB	*GMV-2158*	14.3	18	1.26	[79]
BB	*GMV-2159*	17.2	21.6	1.26	[79]
BB	*Jeholornis*	55.4	68.3	1.23	[37]
BB	*Jixiangornis*	71.9	83.2	1.16	[38]
BB	*Patagopteryx*	103	140	1.36	[39]
BB	*Pengornis*	48	50.4	1.05	[22]
BB	*Sapeornis*	74.4	81.7	1.1	[40]
BB	*Shenzhouraptor*	55.4	68.3	1.23	[41]
BB	*Sinornis*	21	26.4	1.26	[23]
BB	*Yanornis*	66	78	1.18	[80]
BB	*Yixianornis*	41	52.8	1.29	[42]
Ther	*Achillobator*	505	490	0.97	[53]
Ther	*Acrocanthosaurus*	1153	865	0.75	Pers. com. Currie
Ther	*Afrovenator*	760	687	0.9	[81]
Ther	*Albertosaurus*	1080	1030	0.95	[43]
Ther	*Allosaurus*	850	690	0.81	[24]
Ther	*Alxasaurus*	555	481	0.87	[44]
Ther	*Anchiornis*	66.2	106.4	1.61	[25]
Ther	*Australovenator*	578	569	0.98	[82]
Ther	*Bambiraptor*	118	170	1.44	[26]
Ther	*Beipiaosaurus*	265	275	1.04	[83]
Ther	*Caudipteryx*	152	196	1.29	[48]
Ther	*Caudipteryx*	145	183	1.26	[49]
Ther	*Caudipteryx*	145	188	1.3	[49]

Ther	*Ceratosaurus*	759	594	0.78	[84]
Ther	*Ceratosaurus*	630	520	0.83	[84]
Ther	*Ceratosaurus*	620	555	0.9	[84]
Ther	*Chilantosaurus*	1190	954	0.8	[85]
Ther	*Citipati*	415	450	1.08	Pers. com Balanoff
Ther	*Coelophysis*	209	224	1.07	[51]
Ther	*Coelophysis*	123	136	1.11	[51]
Ther	*Compsognathus*	67	87.7	1.31	[27]
Ther	*Compsognathus*	108.8	131.8	1.21	[27]
Ther	*Daspletosaurs*	1005	1022	1.02	[43]
Ther	*Deinonychus*	336	368	1.1	[53]
Ther	*Delatdromeus*	740	700	0.95	[86]
Ther	*Dilophosaurus*	557	555	1	[54]
Ther	*Elaphrosaurus*	520	608	1.17	[87]
Ther	*Epidendrosaurus*	16.5	19.25	1.17	[55]
Ther	*Epidendrosaurus*	16.2	18.9	1.17	[55]
Ther	*Epidexipteryx*	51	63	1.24	[55]
Ther	*Eustreptospondylus*	498	479	0.96	[88]
Ther	*Falcarius*	394	430	1.09	Pers com Zanno
Ther	*Gallimimus*	665	740	1.11	[58]
Ther	*Gallimimus*	270	306	1.13	[58]
Ther	*Gaurdimimus*	371	388	1.05	[89]
Ther	*Gigantoraptor*	1100	1180	1.07	[90]
Ther	*Gorgosaurus*	1040	1000	0.96	Pers. Com. Currie
Ther	*Herrerasaurus*	345	345	1	[62]
Ther	*Huxiagnathus*	163.28	183.41	1.12	[64]
Ther	*Jinfengopteryx*	70.31	100.5	1.43	[65]
Ther	*Jurvenator*	52	58.1	1.12	[66]
Ther	*Khaan*	193	240	1.24	Pers. com. Balanoff
Ther	*Limusaurus*	208	249	1.2	[67]
Ther	*Mahakala*	79	110	1.39	[68]
Ther	*Mapusaurus*	1300	1075	0.83	[85]
Ther	*Mei long*	81	108	1.33	[30]
Ther	*Microraptor gui*	97	124.4	1.28	[7]
Ther	*Microraptor*	49.8	66	1.33	Pers. com Xu
Ther	*Microraptor*	74.75	94.22	1.26	[31]
Ther	*Microraptor*	74.77	95.51	1.28	[31]
Ther	*Mononykus*	138.6	175.2	1.26	[69]

Ther	*Neimongosaurus*	366	310	0.85	[91]
Ther	*Neovenator*	750	670	0.89	[92]
Ther	*Neqwebasaurus*	118	140.7	1.19	[93]
Ther	*Northronychus*	692	614	0.89	[70]
Ther	*Ornithomimus*	435	475	1.09	[34]
Ther	*Parvicursor*	52.6	75.6	1.44	[94]
Ther	*Procompsognathus*	93.1	112.6	1.21	[95]
Ther	*Protoarchaeopteryx*	147	188	1.28	[73]
Ther	*Rahonavis*	88	119.8	1.36	[96]
Ther	*Raptorex*	338	397	1.17	[60]
Ther	*Saurornitholestes*	214	283	1.32	[97]
Ther	*Saurornitthodes*	198	243	1.23	[87]
Ther	*Similicaudipteryx*	220	240	1.09	[98]
Ther	*Sinoraptor*	876	776	0.89	[99]
Ther	*Sinornithoides*	140	190.6	1.36	[32]
Ther	*Sinornithomimus*	323	347	1.07	[33]
Ther	*Sinosauropteryx*	53.2	61	1.15	Pers. com. Currie
Ther	*Sinosauropteryx*	86.4	97	1.12	Pers. com. Currie
Ther	*Struthithomimus*	480	535	1.11	[34]
Ther	*Struthithomimus*	502	556	1.11	[34]
Ther	*Suchomimus*	1075	945	0.88	[85]
Ther	*Syntarus*	208	223	1.07	[100]
Ther	*Tanycolagreus*	356	387	1.09	[76]
Ther	*Tarbosaurus*	970	880	0.91	Pers. com Currie
Ther	*Tianyuraptor*	200	260	1.3	Pers com. Sullivan
Ther	*Tugulusaurus*	215	240	1.12	[101]
Ther	*Tyrannosaurus*	1320	1245	0.94	Pers. com. Currie
Ther	*Tyrannosaurus*	1280	1166	0.91	Pers. com. Currie
Ther	*Velociraptor*	238	255	1.07	[103]
Ther	*Yangchuanosaurus*	850	755	0.89	[104]
