# Supplementary material for: Assessing Arboreal Adaptations of Bird Antecedents: Testing the Ecological Setting of the Origin of the Avian Flight Stroke
Source: PLoS One. 2011 Aug 9;6(8):e22292. doi: 10.1371/journal.pone.0022292 (PMC3153453; doi:10.1371/journal.pone.0022292)
Supplement: Table S10 — Bird hindlimb lengths and relative length scores. M = mass, F = femur length, T = tibia length, TMT = tarsometatarsus length, Leg L = leg length (F+T+TMT), Rleg L = (F+T+TMT)/M0.33, TL = T/M0.33, RTL = TL/Avg, TMTL = TMT/M0.33, R TMTL = TMTL/Avg. (PDF) [file pone.0022292.s023.pdf]

category	taxon	M (kg)	Leg L (mm)	F (mm)	T (mm)	TMT (mm)	Rleg L	TL	RTL	TMTL	R TMTL	Ref
A	*Alcedo atthis*	0.027	50.1	15.9	25.2	9	167.0	84.0	0.78	30.0	0.39	[6]
A	*Ara macao*	1.015	176.5	62	84.3	30.2	175.6	83.9	0.78	30.1	0.4	[6]
A	*Carduelis chloris*	0.026	62.2	17	27.7	17.5	210.0	93.5	0.86	59.1	0.78	[10]
A	*Chaetura pelagic*	0.0236	51.3	15.8	23.7	11.8	178.8	82.6	0.76	41.1	0.54	[6]
A	*Coccothraustes coccothraustes*	0.058	76.9	21.5	34.2	21.2	198.7	88.4	0.82	54.8	0.72	[10]
A	*Coccyzus erythrophthalmus*	0.059	100	30.8	42.2	27	256.9	108.4	1	69.4	0.91	[7]
A	*Delichon urbica*	0.0145	44.8	13.1	20.6	11.1	183.7	84.5	0.78	45.5	0.6	[10]
A	*Ficedula hypoleuca*	0.0122	55	13.4	24.4	17.2	238.9	106.0	0.98	74.7	0.98	[10]
A	*Hirundo daurica*	0.0222	50.5	13.6	23.3	13.6	179.7	82.9	0.77	48.4	0.64	[10]
A	*Loxia curvirostra*	0.0364	67.7	19.5	30.7	17.5	204.3	92.6	0.86	52.8	0.69	[10]
A	*Muscicapa striata*	0.0159	51.5	13.8	23	14.7	204.8	91.5	0.84	58.5	0.77	[10]
A	*Opisthocomus hoazin*	0.696	203	62.8	86.7	53.5	229.1	97.8	0.91	60.4	0.8	[6]
A	*Regulus regulus*	0.0059	48	9.7	21.2	17.1	265.6	117.3	1.08	94.6	1.24	[8]
A	*Riparia riparia*	0.013	40.5	11.4	19.1	10	172.2	81.2	0.75	42.5	0.56	[8]
Bop	*Bubo virginanus*	1.154	257.5	76.9	118.6	62	245.5	113.1	1.06	59.1	0.79	[15]
Bop	*Buteo jamaicensis*	1.028	277.6	83.6	109.6	84.4	275.1	108.6	1.02	83.6	1.11	[15]
BOP	*Falco sparverius*	0.113	119.9	33.9	49.3	36.7	248.0	102.0	0.95	75.9	1	[15]
BOP	*Strix varia*	0.632	230.8	69.7	103.4	57.7	268.9	120.5	1.12	67.2	0.89	[15]
C	*Certhia familiaris*	0.0091	45.1	10.8	19	15.3	216.0	91.0	0.84	73.3	0.96	[8]
C	*Dryocopus pileatus*	0.308	131.4	41.9	53.5	36	194.6	79.2	0.74	53.3	0.71	[6]
C	*Melanerpes erythrocephalus*	0.0716	98.2	30.5	40.7	27	236.5	98.0	0.91	65.0	0.86	[6]
C	*Sitta europaea*	0.0226	61.6	16	26.5	19.1	217.9	93.7	0.87	67.6	0.89	[10]
G	*Luscinia megarhynchos*	0.0183	79.5	18.5	34.4	26.6	301.7	130.5	1.2	100.9	1.32	[10]
G	*Alauda amensis*	0.037	74.1	19.3	31.5	23.3	222.4	94.5	0.87	69.9	0.92	[10]
G	*Cinclus cinclus*	0.0642	97.7	23.4	43.6	30.7	244.0	108.9	1.01	76.7	1.01	[11]
G	*Columba livia*	0.348	136.8	45.1	59.1	32.6	194.5	84.0	0.78	46.3	0.61	[12]
G	*Corvus corax*	1.097	257.8	71.9	114.4	71.5	250.0	110.9	1.04	69.3	0.92	[11]
G	*Corvus frugilegus*	0.489	196.7	50.9	89	56.8	249.7	113.0	1.05	72.1	0.96	[6]
G	*Crotophaga ani*	0.111	100	25.3	44.2	30.5	208.1	92.0	0.85	63.5	0.84	[7]
G	*Fringilla coelebs*	0.0219	62.7	16.2	28.1	18.4	224.1	100.4	0.93	65.8	0.86	[10]
G	*Fringilla montiiringilla*	0.0226	64.5	16.6	29.3	18.6	228.1	103.6	0.96	65.8	0.86	[10]
G	*Galerida theckla*	0.0368	78.6	20.3	33.2	25.1	236.3	99.8	0.92	75.5	0.99	[10]
G	*Geococcyx sp.*	0.376	201.6	53.9	85	62.7	279.3	117.8	1.1	86.9	1.15	[7]
G	*Goura crisata*	2	294.8	79	124.2	91.6	232.0	99.8	0.92	73.1	0.97	[13]
G	*Lullula arborea*	0.0269	69.5	17.7	29.9	21.9	247.9	94.4	0.92	94.7	0.96	[10]
G	*Melanocorypha calandra*	0.0616	97.9	23.2	37.3	37.4	223.6	98.9	0.87	74.8	1.25	[10]
G	*Oenanthe hispanica*	0.0164	82.5	19.9	36	26.6	324.7	141.7	1.31	104.7	1.37	[10]
G	*Oenanthe leucura*	0.041	77.1	17.2	34.1	25.8	240.1	107.9	0.91	78.2	0.98	[10]
G	*Parus alter*	0.0091	50	11.8	22.2	16	239.5	106.3	0.98	76.6	1	[8]
G	*Parus caeruleus*	0.011	53.4	12	24	17.4	237.2	103.8	0.99	77.8	1.03	[10]

G	*Parus cristatus*	0.0122	54.6	12.8	23.9	17.9	240.4	107.3	0.96	74.9	1.02	[8]
G	*Parus montanus*	0.0109	53.3	12.9	23.8	16.6	274.2	118.2	0.99	93.5	0.98	[8]
G	*Phoenicurus ochruros*	0.0165	69.8	15.9	30.1	23.8	267.0	117.6	1.09	88.2	1.23	[10]
G	*Phoenicurus phoenicurus*	0.0147	65.4	15	28.8	21.6	262.3	112.8	1.08	81.4	1.16	[10]
G	*Pica pica*	0.221	158.6	41.2	68.2	49.2	260.3	114.1	1.05	86.2	1.08	[6]
G	*Saxicola rubetra*	0.0166	66.4	15.3	29.1	22	269.5	117.9	1.05	90.6	1.13	[10]
G	*Saxicola torquatus axillaris*	0.0155	67.2	15.2	29.4	22.6	258.4	111.1	1.09	72.4	1.19	[10]
G	*Sturnus cineraceus*	0.071	107	31	46	30	236.6	104.7	1.03	69.4	0.96	[12]
G	*Sturnus vulgaris*	0.0876	105.1	27.8	46.5	30.8	243.4	107.1	0.97	72.8	0.91	[11]
G	*Turdus iliacus*	0.0612	95.9	25	42.2	28.7	226.5	98.5	0.99	68.7	0.96	[10]
G	*Turdus merula merula*	0.113	109.5	28.7	47.6	33.2	255.1	111.2	0.91	79.0	0.91	[10]
G	*Turdus philomelos*	0.0689	104.6	26.6	45.6	32.4	231.5	101.9	1.03	68.5	1.04	[10]
G	*Turdus visciuorus*	0.112	111.6	29.5	49.1	33	234.0	98.6	0.95	72.7	0.9	[10]
Gb	*Alectoris rufa*	0.516	169.4	55.4	72.8	41.2	211.2	90.8	0.85	51.4	0.68	[6]
Gb	*Anas acuta*	0.5	182.7	54.6	84.2	43.9	230.2	106.1	0.99	55.3	0.73	[12]
Gb	*Anhima cornuta*	3.15	409	99	184	126	279.0	125.5	1.18	86.0	1.15	[12]
Gb	*Ardea cinerea*	1.12	398	62	179	157	383.2	172.4	1.61	151.2	2.01	[12]
Gb	*Cariama cristata*	1.4	480	82	205	193	429.1	183.3	1.71	172.5	2.3	[6]
Gb	*Chroicocephalus ridibundus*	0.228	164.5	42.4	75.9	46.2	269.3	124.2	1.16	75.6	1	[12]
Gb	*Dromaius novaehollandiae*	35	973	219	393	361	395.1	185.0	1.14	131.6	1.49	[14]
Gb	*Egretta garzetta*	0.376	285.2	56.7	133.5	95	297.5	120.1	1.72	110.4	1.74	[12]
Gb	*Gallus gallus*	0.988	313	91	128	94	314.3	128.5	1.2	94.4	1.26	[11]
Gb	*Meleagris gallopavo*	7.8	537	139	230	168	270.8	116.0	1.09	84.7	1.13	[6]
Gb	*Rhea sp.*	25	851	211	322	318	291.0	110.1	1.04	108.8	1.46	[14]
Gb	*Struthio camelus*	111	1301	300	524	477	270.7	109.0	1.04	99.3	1.34	[14]
Avg								107.7		75.7		
