# Supplementary material for: Assessing Arboreal Adaptations of Bird Antecedents: Testing the Ecological Setting of the Origin of the Avian Flight Stroke
Source: PLoS One. 2011 Aug 9;6(8):e22292. doi: 10.1371/journal.pone.0022292 (PMC3153453; doi:10.1371/journal.pone.0022292)
Supplement: Table S11 — Theropod hindlimb lengths and relative length scores. Est C = mass estimate based on femoral circumference from Christiansen and Farina 2004, Est L = estimate based on femoral length from Christiansen and Farina 2004, M = mass used in kg, F = femur length, T = tibia length, Mt = metatarsus length, Leg L = leg length (F+T+Mt), Rleg L = (F+T+TMT)/M0.33, TL = T/M0.33, RTL = TL/Avg, TMTL = TMT/M0.33, R TMTL = TMTL/Avg. (PDF) [file pone.0022292.s024.pdf]

category	taxon	F	Est C	Est L	M	Tibia	MtIII	Leg L	Rleg L	TL	RTL	MtL	RMTL
BB	*Archaeopteryx*	53	0.18	0.18	0.18	71	37	161	0.29	126.2	1.2	65.8	1.1
BB	*Archaeopteryx*	37	0.15	0.06	0.06	53	30	120	0.30	132.9	1.3	76.4	1.3
BB	*Archaeopteryx*	61	0.22	0.29	0.22	81	44	185	0.30	132.7	1.3	72.5	1.2
BB	*Archaeopteryx*	48	0.12	0.12	0.12	71	41	160	0.33	145.0	1.4	82.7	1.4
BB	*Archaeopteryx*	70	0.95	0.39	0.40	90	48	207	0.28	121.1	1.1	64.3	1.1
BB	*Archaeopteryx*	50	X	X	0.15	75	40	165	0.31	139.5	1.3	74.1	1.2
BB	*Confuciusornis*	45	X	0.20	0.20	55	26	126	0.21	93.6	0.9	44.2	0.7
BB	*Confuciusornis*	33	X	0.06	0.06	41	20	94	0.24	103.8	1.0	50.6	0.9
BB	*Dalianraptor*	49	0.77	0.14	0.14	68	43	160	0.31	130.1	1.2	82.3	1.4
BB	*Jeholornis*	75	0.57	X	0.57	94	53	222	0.27	112.7	1.1	63.8	1.1
BB	*Jixiangornis*	72	X	0.50	0.50	83	41	196	0.25	104.6	1.0	51.9	0.9
BB	*Patagopteryx*	103	4.1	1.6	1.6	140	52	295	0.25	119.9	1.1	44.5	0.8
BB	*Pengornis*	48	X	0.15	0.15	50	27	125	0.23	94.3	0.9	49.6	0.8
BB	*Sapeornis*	80	X	0.71	0.71	84	45	209	0.23	93.6	0.9	49.9	0.8
BB	*Shenzhouraptor*	55	0.77	0.21	0.21	68	35	158	0.26	114.3	1.1	57.9	1.0
BB	*Sinornis*	21	X	0.01	0.01	26	15	62	0.28	120.7	1.1	66.7	1.1
BB	*Yixianornis*	41	0.29	0.90	0.09	53	27	121	0.27	116.9	1.1	59.8	1.0
Ther	*Achillobator*	505	767	264	264	490	234	1229	0.20	77.8	0.7	37.2	0.6
Ther	*Allosaurus*	850	1403	1547	1400	690	327	1867	0.17	63.2	0.6	30.0	0.5
Ther	*Anchiornis*	66	X	X	0.40	106	55	228	0.31	144.0	1.4	74.7	1.3
Ther	*Australovenator*	578	773	408	410	569	322	1469	0.20	78.1	0.7	44.2	0.7
Ther	*Austroraptor*	560	665	369	370	565	330	1455	0.21	80.3	0.8	46.9	0.8

Ther	*Bambiraptor*	118	3.8	2.4	2.5	170	81	369	0.27	125.6	1.2	59.9	1.0
Ther	*Beipiaosaurus*	265	160.0	31.0	31.0	275	107	647	0.21	88.6	0.8	34.5	0.6
Ther	*Caudipteryx dongi*	152	13.0	5.5	5.5	196	124	472	0.27	111.7	1.1	70.7	1.2
Ther	*Caudipteryx sp.*	145	11.2	4.7	4.7	183	112	440	0.26	109.8	1.0	67.2	1.1
Ther	*Caudipteryx zoui*	145	9.4	4.7	4.7	188	113	446	0.27	112.8	1.1	67.8	1.1
Ther	*Ceratosaurus*	759	964	982	960	594	X	X	X	61.6	0.6	X	X
Ther	*Coelophysis*	209	X	15	15	224	126	559	0.23	91.7	0.9	51.6	0.9
Ther	*Coelophysis*	123	X	2.6	2.6	136	82	341	0.25	99.2	0.9	59.8	1.0
Ther	*Compsognathus*	109	4.1	1.9	1.9	132	81	322	0.26	106.6	1.0	65.5	1.1
Ther	*Deinonychus*	336	96	71	71	368	164	868	0.21	90.1	0.9	40.3	0.7
Ther	*Delatdromeus*	740	399	905	400	700	450	1890	0.26	96.9	0.9	62.3	1.1
Ther	*Dilophosaurus*	557	399	350	350	555	300	1412	0.20	80.3	0.8	43.4	0.7
Ther	*Elaphrosaurus*	520	287	307	287	608	378	1506	0.23	93.9	0.9	58.4	1.0
Ther	*Eoraptor*	152	12	6	6	157	81	390	0.22	86.9	0.8	44.8	0.8
Ther	*Epidendrosaurus*	17	X	0.01	0.01	19	12	48	0.27	110.6	1.0	69.0	1.2
Ther	*Epidendrosaurus*	16	X	0.01	0.01	19	12	47	0.27	108.6	1.0	68.4	1.2
Ther	*Epidexipteryx*	51	0.49	0.16	0.16	63	31	145	0.27	115.3	1.1	56.8	1.0
Ther	*Eustreptospondylus*	498	350	273	275	479	232	1209	0.19	75.1	0.7	36.4	0.6
Ther	*Gallimimus*	665	442	689	442	740	530	1935	0.26	99.1	0.9	71.0	1.2
Ther	*Gigantoraptor*	1100	2260	3246	2260	1177	583	2860	0.22	92.0	0.9	45.6	0.8
Ther	*Gorgosaurus*	1040	5262	2710	2710	1000	594	2634	0.19	73.6	0.7	43.7	0.7
Ther	*Guaibasaurus*	206	27.2	16.6	16.6	212	95	513	0.20	83.9	0.8	37.6	0.6
Ther	*Guanlong*	416	X	142	142	424	360	1200	0.23	82.6	0.8	70.2	1.2
Ther	*Haplocheirus*	214	X	16.7	16.7	269	134	617	0.24	106.3	1.0	52.8	0.9
Ther	*Heyuannia*	255	55	29	29	320	135	710	0.23	105.3	1.0	44.4	0.8
Ther	*Huxiagnathus*	163	13	7	7	183	110	456	0.24	96.5	0.9	57.7	1.0
Ther	*Jinfengopteryx*	70	X	0.46	0.46	101	60	231	0.30	129.	1.2	77.5	1.3

										9			
Ther	*Khaan*	193	24	12	12	240	110	543	0.24	105.7	1.0	48.5	0.8
Ther	*Mahakala*	79	X	X	0.40	110	51	240	0.32	148.8	1.4	69.0	1.2
Ther	*Mei long*	81	X	X	0.40	108	58	247	0.33	146.1	1.4	78.5	1.3
Ther	*Microraptor gui*	97	0.77	1.30	0.77	124	70	292	0.32	135.6	1.3	76.4	1.3
Ther	*Microraptor zhaoianus*	75	0.30	0.60	0.30	94	48	217	0.32	140.2	1.3	71.1	1.2
Ther	*Microraptor zhaoianus*	75	0.30	0.60	0.30	96	49	220	0.33	142.1	1.3	73.5	1.2
Ther	*Ornitholestes*	215	24.1	16.9	16.9	X	119	X	X	X	X	46.8	0.8
Ther	*Ornithomimus*	435	89	173	89	475	310	1220	0.28	108.0	1.0	70.5	1.2
Ther	*Parvicursor*	53	0.14	0.18	0.14	76	58	186	0.36	144.6	1.4	111.2	1.9
Ther	*Procompsognathus*	93	1.9	1.3	1.3	113	69	275	0.25	103.3	1.0	63.6	1.1
Ther	*Protoarchaeopteryx*	147	15.0	2.6	2.6	188	115	450	0.33	137.2	1.3	83.9	1.4
Ther	*Rahonavis*	88	0.68	0.91	0.68	120	48	256	0.29	136.1	1.3	54.5	0.9
Ther	*Raptorex*	338	X	72.5	72.5	397	266	1001	0.24	96.6	0.9	64.7	1.1
Ther	*Saurornitholestes*	198	X	16.0	16.0	243	139	580	0.23	97.3	0.9	55.7	0.9
Ther	*Similicaudipteryx*	220	20.7	18.2	18.2	240	183	643	0.25	92.1	0.9	70.3	1.2
Ther	*Sinoraptor*	876	1095	1522	1095	776	410	2062	0.20	77.1	0.7	40.7	0.7
Ther	*Sinornithoides*	140	5.4	4.3	4.3	191	111	442	0.27	117.8	1.1	68.6	1.2
Ther	*Sinornithosaurus*	148	3.6	5.1	3.6	X	93	X	X	X	X	60.9	1.0
Ther	*Sinosauropteryx*	86	X	0.90	0.90	97	64	247	0.26	100.4	1.0	66.2	1.1
Ther	*Sinosauropteryx*	53	1.12	0.20	0.20	61	40	154	0.26	103.8	1.0	68.0	1.1
Ther	*Struthithomimus*	480	142	233	142	535	365	1380	0.27	104.3	1.0	71.1	1.2
Ther	*Struthithomimus*	502	182	258	182	556	398	1456	0.26	99.8	0.9	71.5	1.2
Ther	*Syntarus*	208	12.8	14.0	12.8	223	132	563	0.24	96.1	0.9	56.9	1.0

Ther	*Tarbosaurus*	1120	5423	3440	3440	890	530	2540	0.17	60.6	0.6	36.1	0.6
Ther	*Tyrannosaurus*	1320	6752	5400	5400	1245	671	3236	0.19	73.0	0.7	39.4	0.7
Ther	*Velociraptor*	238	16.9	23.4	16.9	255	99	592	0.23	100.3	1.0	39.0	0.7
								Avg	0.25	105.3		59.2	
