# Supplementary material for: Assessing Arboreal Adaptations of Bird Antecedents: Testing the Ecological Setting of the Origin of the Avian Flight Stroke
Source: PLoS One. 2011 Aug 9;6(8):e22292. doi: 10.1371/journal.pone.0022292 (PMC3153453; doi:10.1371/journal.pone.0022292)
Supplement: Table S12 — Manual phalangeal indices of non-avian theropods and early avians. A) Digit II B) Digit III. (PDF) [file pone.0022292.s025.pdf]

A)

category	taxon	McII	PhII-1	PhII-2	Ph total	P.I.	Ref
BB	*Archaeopteryx*	28.0	15.4	19.4	34.8	1.24	[35]
BB	*Archaeopteryx*	17.8	10.1	14.5	24.6	1.38	[35]
BB	*Archaeopteryx*	25.0	12.5	18.5	31.0	1.24	[35]
BB	*Archaeopteryx*	34.3	19.0	27.0	46.0	1.34	[35]
BB	*Archaeopteryx*	23.5	12.8	18.6	31.4	1.34	[35]
BB	*Dalianraptor*	23.0	22.0	19.0	41.0	1.78	[28]
BB	*Jeholornis*	36.7	17.8	19.2	37.0	1.01	[37]
BB	*Jixiangornis*	44.9	22.7	21.1	43.8	0.98	[38]
BB	*Sapeornis*	57.1	30.0	27.7	57.7	1.01	[40]
BB	*Sapeornis*	51.3	29.0	25.9	54.9	1.07	[40]
BB	*Sapeornis*	52.9	30.6	26.6	57.2	1.08	[40]
BB	*Shenzhouraptor*	36.7	17.8	19.2	37.0	1.01	[41]
BB	*Sinornis*	10.7	5.4	3.8	9.2	0.86	[23]
BB	*Yixianornis*	25.6	12.5	12.3	24.8	0.97	[42]
Ther	*Acrocathosaurus*	116.0	101.0	103.0	204.0	1.76	Pers. com. Currie
Ther	*Albertosaurus*	83.0	41.0	58.0	99.0	1.19	[43]
Ther	*Allosaurus*	125.0	94.0	102.0	196.0	1.57	[24]
Ther	*Alxasaurus*	111.0	72.0	80.0	152.0	1.37	[44]
Ther	*Anchiornis*	33.9	21.0	27.0	48.0	1.42	[25]
Ther	*Bambiraptor*	47.8	21.4	35.0	56.4	1.18	[26]
Ther	*Caudipteryx*	29.0	18.5	25.0	43.5	1.5	[48]
Ther	*Caudipteryx*	28.0	19.0	24.0	43.0	1.54	[49]
Ther	*Caudipteryx*	28.0	101.0	103.0	41.0	1.46	[49]
Ther	*Citipati*	120.0	-	-	190.0	1.58	Pers. com Balanoff
Ther	*Coelurus*	53.0	46.0	48.5	94.5	1.78	[50]
Ther	*Coleophysis*	39.2	18.6	21.0	39.6	1.01	[51]
Ther	*Compsognathus*	17.6	7.8	14.5	22.3	1.27	[27]
Ther	*Compsognathus*	27.3	13.4	24.0	37.4	1.37	[27]
Ther	*Deinonochurieus*	230.0	140.0	226.0	366.0	1.59	[104]
Ther	*Deinonychus*	88.3	54.0	76.5	130.5	1.48	[52]
Ther	*Deinonychus*	93.7	55.3	78.0	133.3	1.42	[52]
Ther	*Dilong*	43.0	28.0	43.0	71.0	1.65	Pers. com. Currie
Ther	*Dilophosaurus*	105.0	70.0	63.0	133.0	1.27	[54]
Ther	*ek troodon*	40.0	26.0	24.0	50.0	1.25	[105]
Ther	*Epidendrosaurus*	5.2	3.2	5.4	8.6	1.65	[55]
Ther	*Epidendrosaurus*	5.5	3.1	5.2	8.3	1.51	[55]
Ther	*Erliansaurus*	116.0	40.0	45.0	85.0	0.73	[106]

Ther	*Falcarius*	91.0	69.0	85.0	154.0	1.69	[56]
Ther	*Falcarius*	96.0	69.0	87.0	156.0	1.63	[56]
Ther	*Gallimimus*	115.0	53.0	100.0	153.0	1.33	[58]
Ther	*Gorgosaurus*	98.0	57.0	83.0	140.0	1.43	Pers. com Currie
Ther	*Hagaryphus*	122.0	95.0	99.0	194.0	1.59	[107]
Ther	*Harpymimus*	94.0	49.0	104.0	153.0	1.63	[61]
Ther	*Herrerasaurus*	66.0	47.0	51.0	98.0	1.48	[108]
Ther	*Huxiagnathus*	39.2	27.4	33.4	60.8	1.55	Pers. Com Hwang
Ther	*Jinfengopteryx*	21.4	15.3	21.1	36.3	1.7	[65]
Ther	*Jurvenator*	11.5	8.0	10.0	18.0	1.57	[66]
Ther	*Khann*	53.0	-	-	0.0	1.7	Pers. Com. Balanoff
Ther	*Limusaurus*	12.0	6.2	4.3	10.5	0.88	[67]
Ther	*Megaraptor*	170.0	108.0	104.0	212.0	1.25	[109]
Ther	*Microraptor*	31.3	13.3	16.4	29.7	0.95	[31]
Ther	*Microraptor*	22.3	11.3	11.8	23.1	1.04	Pers. com Xu
Ther	*Northonychus*	141.2	102.0	133.4	235.4	1.67	[70]
Ther	*Ornithomimus*	100.0	36.0	90.0	126.0	1.26	[34]
Ther	*Raptorex*	20.0	13.0	22.0	35.0	1.75	[60]
Ther	*Sinornithoides*	33.5	18.5	28.5	47.0	1.4	[32]
Ther	*Sinornithomimus*	54.7	19.9	59.9	79.8	1.46	[33]
Ther	*Sinornithosaurus*	63.0	32.0	35.0	67.0	1.06	Pers. com Xu
Ther	*Sinosauropteryx*	10.2	4.7	6.0	10.7	1.05	Pers. com Currie
Ther	*Sinosauropteryx*	17.0	7.9	8.9	16.8	0.99	Pers. com Currie
Ther	*Sinosauropteryx*	21.0	13.9	18.5	32.4	1.54	Pers. com Currie
Ther	*Struthithomimus*	103.0	44.0	89.0	133.0	1.29	[34]
Ther	*Syntarus*	26.0	13.0	17.0	30.0	1.15	[100]
Ther	*Tanycolagreus*	81.0	65.0	75.0	140.0	1.73	[76]
Ther	*Tarbosaurus*	75.0	34.0	62.0	96.0	1.28	Pers. com Currie
Ther	*Tianyuraptor*	57.0	32.0	42.0	74.0	1.3	Pers com. Sullivan
Ther	*Tyrannosaurus*	95.0	60.0	79.0	139.0	1.46	Pers. com Currie
Ther	*Velociraptor*	50.8	31.3	45.8	77.1	1.52	[102]
Ther	*Yixianosaurus*	36.0	26.0	38.0	64.0	1.78	[78]

B)

category	taxon	McIII	III-1	III-2	III-3	PI	3/(1+2)	Reference
BB	*Archaeopteryx*	22	4.8	4.2	12.9	1	1.43	[35]
BB	*Archaeopteryx*	24.5	6.4	4	12.3	0.93	1.18	[35]
BB	*Archaeopteryx*	X	7.9	6.1	17.5	X	1.25	[35]
BB	*Archaeopteryx*	16.5	4.8	2.2	9.5	1	1.36	[35]
BB	*Dalianrapter*	22	5	12	13	1.36	0.76	[28]
Ther	*Acrocanthosaurus*	89	50	42	60	1.71	0.65	Pers. com. Currie
Ther	*Allosaurus*	105	50	41	52	1.36	0.57	[24]
Ther	*Anchiornis*	30.5	8.1	7.2	14.2	0.97	0.93	[25]
Ther	*Bambiraptor*	44.9	15.5	6.3	23.5	1.01	1.08	[26]
Ther	*Coelophysis*	35.9	12.6	15.4	17.5	1.27	0.63	[51]
Ther	*Deinoncherus*	246	110	104	186	1.63	0.87	[104]
Ther	*Deinonychus*	73.4	21.7	15.5	47.3	1.15	1.27	[52]
Ther	*Dilophosaurus*	115	41	44	45	1.13	0.53	[54]
Ther	*Elmisaurus*	X	30	30	43	X	0.72	[110]
Ther	*Epidendrosaurus*	5.75	7.1	6.5	6	3.41	0.44	[55]
Ther	*Epidendrosaurus*	X	7.3	6.8	6.3	X	0.45	[55]
Ther	*Erlianosaurus*	79	14	17	33	0.81	1.06	[106]
Ther	*Falcarius*	75	31	30	48	1.45	0.79	[56]
Ther	*Gallimimus*	105	32	36	74	1.35	1.09	[58]
Ther	*Hagryphus*	8.7	5.4	4.5	6	1.83	0.61	[107]
Ther	*Harpymimus*	103	31	37	81	1.45	1.19	[61]
Ther	*Herrersaurus*	74	44	35	40	1.61	0.51	[108]
Ther	*Juravenator*	9	4	4.5	5.5	1.56	0.65	[66]
Ther	*Linheraptor*	52	25	12	36	1.4	0.97	[111]
Ther	*Microraptor*	29.8	10.12	4.1	10.37	0.83	0.73	[31]
Ther	*Ornithomimus*	98	31	23	74	1.31	1.37	[34]
Ther	*Sinornithodes*	35	4.2	8.2	20.3	0.93	1.64	[32]
Ther	*Sinornithomimus*	53.8	13.9	14.6	42.9	1.33	1.51	[33]
Ther	*Sinornithosaurus*	60	18	9	23	0.83	0.85	Pers. com Xu
Ther	*Sinosauropteryx*	8.3	3.4	2	3	1.01	0.56	Pers. com Currie
Ther	*Sinosauropteryx*	13.8	4.7	3.6	5	0.96	0.6	Pers. com Currie
Ther	*Struthiomimus*	103	28	28	68	1.2	1.21	[34]
Ther	*Syntarus*	26	10	9	12	1.19	0.63	[100]
Ther	*Velociraptor*	45	17.6	10.1	32.2	1.33	1.16	[102]
Ther	*Velociraptor*	X	19	11.1	33.2	X	1.1	[102]
Ther	*Yixianosaurus*	31	9	8	22	1.26	1.29	[78]
