# Supplementary material for: Assessing Arboreal Adaptations of Bird Antecedents: Testing the Ecological Setting of the Origin of the Avian Flight Stroke
Source: PLoS One. 2011 Aug 9;6(8):e22292. doi: 10.1371/journal.pone.0022292 (PMC3153453; doi:10.1371/journal.pone.0022292)
Supplement: Table S13 — Pedal phalangeal indices of non-avian theropods and early avians. (PDF) [file pone.0022292.s026.pdf]

| category | taxon | PhIII1 | PhIII2 | PhIII3 | sum | PPI | Ref |
|---|---|---|---|---|---|---|---|
| BB | *Archaeopteryx* | 9.6 | 9 | 8.2 | 26.8 | 1.79 | [35] |
| BB | *Archaeopteryx* | 9 | 8 | 7 | 24 | 1.67 | [35] |
| BB | *Archaeopteryx* | 12.7 | 11 | 9.5 | 33.2 | 1.61 | [35] |
| BB | *Archaeopteryx* | 11 | 8.5 | 8.5 | 28 | 1.55 | [35] |
| BB | *Archaeopteryx* | 14 | 13 | 10.5 | 37.5 | 1.68 | [35] |
| BB | *Archaeopteryx* | 10.8 | 9.6 | 7.8 | 28.2 | 1.61 | [35] |
| BB | *Confuciuornis* | 6 | 5 | 5 | 16 | 1.67 | [7] |
| BB | *Dalianraptor* | 14 | 11 | 13 | 38 | 1.71 | [28] |
| BB | *Jeholornis* | 10.9 | 10.6 | 7.3 | 28.8 | 1.64 | [7] |
| BB | *Pengornis* | 8.6 | 7.9 | 8.8 | 25.3 | 1.94 | [7] |
| BB | *Shenzhouraptor* | 10.9 | 10.6 | 7.3 | 28.8 | 1.64 | [41] |
| BB | *Sinornis* | 4.3 | 3.6 | 3.9 | 11.8 | 1.74 | [23] |
| BB | *Yanornis* | 14 | 11 | 10 | 35 | 1.5 | [80] |
| BB | *Yixianornis* | 11.5 | 8.7 | 8.3 | 28.5 | 1.48 | [42] |
| | | | | | | | |
| Ther | *Albertosaurus* | 145 | 105 | 85 | 335 | 1.31 | [43] |
| Ther | *Allosaurus* | 110 | 90 | 66 | 266 | 1.42 | [24] |
| Ther | *Anchiornis* | 12.9 | 11.1 | 10.5 | 34.5 | 1.67 | [25] |
| Ther | *Bambiraptor* | 27.8 | 15.4 | 16.9 | 60.1 | 1.16 | [26] |
| Ther | *Caudipteryx* | 27 | 20 | 17 | 64 | 1.37 | [48] |
| Ther | *Caudipteryx* | 23 | 17 | 13 | 53 | 1.3 | [49] |
| Ther | *Caudipteryx* | 24 | 19 | 15 | 58 | 1.42 | [49] |
| Ther | *Chirostenotes* | 75 | 52 | 58 | 185 | 1.47 | [112] |
| Ther | *Coelophysis* | 36.2 | 30.5 | 26.6 | 93.3 | 1.58 | [51] |
| Ther | *Coelophysis* | 21 | 16 | 13 | 50 | 1.38 | [51] |
| Ther | *Compsognathus* | 17 | 13.65 | 11.5 | 42.15 | 1.48 | [27] |
| Ther | *Deinonychus* | 64.4 | 44 | 41.3 | 149.7 | 1.32 | [52] |
| Ther | *Dilophosaurus* | 110 | 84 | 70 | 264 | 1.4 | [54] |
| Ther | *Epidendrosaurus* | 2.5 | 2 | 2.5 | 7 | 1.8 | [55] |
| Ther | *Eustreptospondylus* | 80 | 65 | 54 | 199 | 1.49 | [88] |
| Ther | *Gallimimus* | 90 | 70 | 50 | 210 | 1.33 | [58] |
| Ther | *Gallimimus* | 44 | 35 | 24 | 103 | 1.34 | [58] |
| Ther | *Gorgosaurus* | 163 | 122 | 93 | 378 | 1.32 | Pers com Currie |
| Ther | *Herrerasaurus* | 55 | 38 | 34 | 127 | 1.31 | [62] |
| Ther | *Huxiagnathus* | 31.17 | 29.44 | 23.62 | 84.23 | 1.7 | Pers. Com Hwang |
| Ther | *Jurvenator* | 11.9 | 8.1 | 7.4 | 27.4 | 1.3 | [66] |
| Ther | *Limusaurus* | 36 | 26 | 20 | 82 | 1.28 | [67] |
| Ther | *Microraptor* | 8.9 | 5.5 | 5 | 19.4 | 1.18 | Pers. com Xu |
| Ther | *Microraptor gui* | 14.4 | 10.3 | 9.8 | 34.5 | 1.4 | [7] |
| Ther | *Microraptor* | 9.95 | 7.74 | 6.13 | 23.82 | 1.39 | [31] |
| Ther | *Microraptor* | 10.05 | 8.24 | 7.88 | 26.17 | 1.6 | [31] |
| Ther | *Ornithomimus* | 74 | 50 | 43 | 167 | 1.26 | [34] |

| | | | | | | | |
|---|---|---|---|---|---|---|---|
| Ther | *Procompsognathus* | 17.1 | 15.2 | 12 | 44.3 | 1.59 | [95] |
| Ther | *Rahonavis* | 19 | 12.4 | 10 | 41.4 | 1.18 | [96] |
| Ther | *Similicaudipteryx* | 46 | 34 | 32 | 112 | 1.43 | [98] |
| Ther | *Sinoraptor* | 124 | 83 | 64 | 271 | 1.19 | [99] |
| Ther | *Sinornithoides* | 27.6 | 20 | 16 | 63.6 | 1.3 | [32] |
| Ther | *Sinornithosaurus* | 25 | 17.5 | 16.5 | 59 | 1.36 | Pers. com Xu |
| Ther | *Sinosauropteryx* | 10 | 7 | 6.8 | 23.8 | 1.38 | Pers. com Currie |
| Ther | *Sinosauropteryx* | 16.7 | 11.2 | 9.4 | 37.3 | 1.23 | Pers. com Currie |
| Ther | *Struthithomimus* | 78 | 54 | 39 | 171 | 1.19 | Pers. com Currie |
| Ther | *Struthithomimus* | 83 | 64 | 52 | 199 | 1.4 | [34] |
| Ther | *Syntarus* | 37 | 29 | 24 | 90 | 1.43 | [100] |
| Ther | *Tanycolagreus* | 73 | 55 | 41 | 169 | 1.32 | [76] |
| Ther | *Tarbosaurus* | 135 | 60 | 100 | 295 | 1.19 | Pers. com Currie |
| Ther | *Tianyuraptor* | 48 | 32 | 27 | 107 | 1.23 | Pers com. Sullivan |
| Ther | *Troodon* | 66 | 41.2 | 39.3 | 146.5 | 1.22 | [105] |
| Ther | *Tyrannosaurus* | 201 | 136 | 122 | 459 | 1.28 | Pers. com Currie |
