# Supplementary material for: Assessing Arboreal Adaptations of Bird Antecedents: Testing the Ecological Setting of the Origin of the Avian Flight Stroke
Source: PLoS One. 2011 Aug 9;6(8):e22292. doi: 10.1371/journal.pone.0022292 (PMC3153453; doi:10.1371/journal.pone.0022292)
Supplement: Table S14 — Phylogenetic nodal reconstructions across Theropoda into basal birds. (PDF) [file pone.0022292.s027.pdf]

taxon	FL	HL	BI	CI	MPI	PPI
Theropoda	0.46	0.96	0.74	1.06	1.36	1.34
Neotheropoda	0.36	0.87	0.66	1.06	1.23	1.38
Ceratosauria	0.29	N/A	0.58	1.04	1.14	1.36
Tetanurae	N/A	0.85	0.62	98	1.29	1.4
Avetheropoda	0.31	0.87	0.7	98	1.49	1.36
Coelurosauria	0.32	0.91	0.74	1.06	1.57	1.35
Coelurosauria A	0.39	0.92	0.77	1.12	1.5	1.4
Maniraptorformes	0.49	0.96	0.8	1.13	1.52	1.42
Maniraptora	0.61	1.04	0.82	1.17	1.53	1.49
Paraves	0.78	1.05	0.82	1.22	1.52	1.61
Eumaniraptora	0.92	1.11	0.89	1.3	1.4	1.54
Troodontidea	0.96	1.21	0.87	1.34	1.4	1.46
Dromeaosaurida	0.9	1.22	0.91	1.28	1.33	1.25
Microraptor sp	1.01	1.41	0.87	1.29	1.08	1.36
Aves	1.04	1.09	0.9	1.3	1.3	1.6
Aves A	1.17	1.03	0.92	1.26	1.18	1.63
Pygostylia	1.09	1.01	0.89	1.23	N/A	1.64
Ornithurae	1.12	1.08	0.85	1.26	1	1.61
