# Supplementary material for: Assessing Arboreal Adaptations of Bird Antecedents: Testing the Ecological Setting of the Origin of the Avian Flight Stroke
Source: PLoS One. 2011 Aug 9;6(8):e22292. doi: 10.1371/journal.pone.0022292 (PMC3153453; doi:10.1371/journal.pone.0022292)
Supplement: Text S1 — References cited in all supplementary information. (PDF) [file pone.0022292.s028.pdf]

1. Argot, C (2004) Functional-adaptive analysis of the postcranial skeleton of a Laventan Borhyaenoid, *Lycopsis longirostris* (Marsupialia, Mammalia). Journal of Vertebrate Paleontology 24(3): 689-70.
2. Renesto S (1994) *Megalancosaurus*, a possibly Arboreal Archosauromorph (Reptilia) from the Upper Triassic of Northern Italy. Journal of Vertebrate Paleontology 14: 38-52.
3. Renesto S and Binelli G (2006) Vallesaurus cenensis Wild 1991, A drepanosaurid (Reptillia, Diapsida) from the late Triassic of Northern Italy.Rivista Italiana di Palentologia e Stratigrafia 112(1):77-94.
4. Fröbisch J, Reisz RR (2009) The Late Permian herbivore *Suminia* and the early evolution of arboreality in terrestrial vertebrate ecosystems. Proceedings of The Royal Society B 276: 3611-8.
5. Hopson JA (2001) Ecomorphology of avian and nonavian theropod phalangeal proportions: Implications for the arboreal versus terrestrial origin of bird flight. In: Gauthier JA, Gall LF, editors. New Perspectives on the Origin and Early Evolution of Birds: Proceedings of the International Symposium in Honour of John H. Ostrom. Peabody Museum of Natural History. pp. 211-227.
6. Dyke G J, Nudds R L and Rayner J M V (2006) Limb disparity and wing shape in pterosaurs. Journal of Evolutionary Biology. 19(4): 1339-1342.
7. Li Z (2008) Morphological study and functional analysis of *Jeholornis prima.* Msc. Thesis.
8. Norberg UM (1979) The Morphology of the wings, legs, and tail of three coniferous forest tits, the goldcrest, and the treecreeper in relation to locomotor pattern and feeding station selection. Philosophical Transactions of the Royal Society *B*. 287 (1019): 131-165.
9. Spring L W (1965) Climbing and pecking adaptations in some North American woodpeckers. The Condor. 67(6): 457-488.
10. Moreno E and Carrascal L M (1993) Ecomorphological patterns of aerial feeding in oscines (Passeriformes: Passeri). Biological Journal of the Linnean Society. 50: 147-165.
11. Gatsey S M (1991) Hind limb scaling in birds and other theropods: Implications for terrestrial locomotion. Journal of Morphology. 209:83-96.
12. Fujita M (2004) Kinematic parameters of the walking of herons, ground-feeders and water-fowl. Comparative Biochemistry and Physiology Part A. 139: 117-124.
13. Livezey B C (1993) An ecomorphological review of the dodo *(Raphus cucullatus)* and solitaire*(Pezophaps solitaria),* flightless Columbiformes of the Mascarene Islands. Journal of Zoology 230: 247-292

14. Dickison M R (2007) The allometry of giant flightless birds. Ph.D thesis.

15. Ward A B, Weigel P D and Conroy R M (2002) Functional morphology of raptor hindlimbs: Implications for resource portioning. The Auk. 119(4): 1052-1063.

16. Li P-peng, Gao K-qin, Hou L-hai, Xu X (2007) A gliding lizard from the Early Cretaceous of China. Proceedings of the National Academy of Sciences of the United States of America 104: 5507-5509.

17. Argot C (2003) Functional adaptations of the postcranial skeleton of two Miocene Borhyaenoids (Mammalia, Metatheria), *Borhyaena* and *Prothylacinus*, from South America. Palaeontology. 46(6): 1213-1267.

18. Doppes D (2001) *Gulo gulo* (Mustelidae, Mammalia) im Jungplesitozän Mitteleuropas. Beiträge zur Paläontologie. 26: 1-95.

19. Steudel K and Beattie J (1993) scaling of cursoriality in mammals. Journal of Morphology. 217: 55-63.

20. Day L M and Jayne BC (2007) Interspecific scaling of the morphology and posture of the limbs during the locomotion of cats (Felidae). The Journal of Experimental Biology. 210: 642-654.

21. Wellnhofer P (1974) Das fünfte Skelette exemplar von *Archaeopteryx*. Palaeontographica Abteilung A .147(4-6): 168-216.

22. Zhou Z, Clarke J A and Zhang F (2008) Insights into diversity, body size and morphological evolution from the largest Early Cretaceous enantiornithine bird. Journal of Anatomy. 212: 565-577.

23. Sereno P C , Rao C and Li J (2002) Sinornis santensis (Aves: Enantiornithines) from the Early Cretaceous of Northeastern China. In: Chiappe LM and Witmer L M Editors. Mesozoic birds: above the heads of dinosaurs. University of California Press. pp. 184-208.

24. Gilmore C W (1920) Osteology of the carnivorous Dinosauria in the United States national Museum, with special reference to the genera *Antrodemus* (*Allosaurus*) and *Ceratosaurus*. Bulletin of the United States National Museum .110: 1-154.

25. Hu D, Hou L, Zhang L, Xu X (2009) A pre-*Archaeopteryx* troodontid theropod from China with long feathers on the metatarsus. Nature 461: 640-3

26. Burnham D A 2004) New information on *Bambiraptor feinbergi* (Theropoda: Dromaeosauridae) from the Late Cretaceous of Montana. In: Currie PJ, Koppelhaus MA, Shugar MA, L WJ, editors. Feathered Dragons. Studies on the Transition from Dinosaurs to Birds. Indiana University Press. pp. 67-111.

27. Peyer K 2006) A reconsideration of *Compsognathus* from the Upper Tithoninan of Canjeurs, Southeastern France. Journal of Vertebrate Paleontology,.26(4): 879-896.

28. Gao C and Liu J (2005). A new avian taxon from Lower Cretaceous Jiufotang Formation of western Liaoning (In Chinese). Global Geology. 24(4):313-316.

29. Zhang F, Zhou Z, Xu X, Wang X (2002) A juvenile coelurosaurian theropod from China indicates arboreal habits. Naturwissenschaften 89: 394-398.

30. Xu X and Norell M A (2004) A new troodontid dinosaur from China with avian-like sleeping posture. Nature. 431: 838-841.

31. Hwang SH, Norell MA, Qiang JI, Keqin GAO (2002) New Specimens of *Microraptor zhaoianus* (Theropoda: Dromaeosauridae) from Northeastern China. American Museum Novitates: 1-44.

32. Russel D A and Dong Z (1993) A nearly complete skeleton of a new troodontid dinosaur from the Early Cretaceous of the Ordos Basin, Inner Mongolia, People's Republic of China. Canadian Journal of Earth Sciences 30: 2163-2173.

33. Kobayashi Y.and Lü J (2003) A new ornithomimid dinosaur with gregarious habits from the Late Cretaceous of China. Acta Palaeontologica Polonica. 48: 235-259.

34. Russell D A (1972) Ostrich dinosaurs from the Late Cretaceous of Western Canada. Canadian Journal of Earth Sciences. 9: 375-402.

35. Mayr G, Pohl B, Hartman S and Peters DS (2007) The tenth skeletal specimen of *Archaeopteryx*. Zoological Journal of the Linnean Society 149: 97-116.

36. Chiappe L M, Ji S, JI Q and Norell M A (1999) Anatomy and systematics of the Confuciusornithidae (Theropoda: Aves) from the Late Mesozoic of Northeastern China. Bulletin of the American Museum of Natural History. 242: 1-89.

37. Zhou Z and Zhang F (2002) A long-tailed, seed-eating bird from the Early Cretaceous of China. Nature. 418: 405-409

38. Ji Q, Ji S, Zhang H, You H, Zhang J, Wang L Yuan C and Ji X(2002) A new avialan bird —*Jixiangornis orientalis* gen. et sp. nov.—from the Lower Cretaceous of Western Liaoning. Journal of Nanjing University (Natural Science Edition). 38(6): 723-736.

39. Chiappe L M (2002) Osteology of the flightless *Patagopteryx deferrariisi* from the Late Cretaceous of Patagonia (Argentina). In L. M. Chiappe & L. M. Witmer editors. Mesozoic Birds: Above the heads of dinosaurs. University of California Press pp. 281-316.

40. Zhou Z and Zhang F (2003) Anatomy of the primitive bird *Sapeornis chaoyangensis* from the Early Cretaceous of Liaoning, China. Canadian Journal of Earth Sciences 40: 731-747.

41. Ji Q, Ji S , You H, Zhang J, Yuan C, Ji X, Li J and Li Y (2003) An early Cretaceous Avialian bird , *Shenzhouraptor sinensis* from Western Liaoning, China. Acta Geologica Sinica. 77(1): 21-27.

42. Clarke J A, Zhou Z and Zhang F (2006) Insight into the evolution of avian flight from a new clade of Early Cretaceous ornithurines from China and the morphology of *Yixianornis grabaui*. Journal of anatomy. 208(3): 287-308.

43. Russell D A (1970) Tyrannosaurs from the Late Cretaceous of Western Canada. National Museum of Natural Sciences Publicatios in Paleontology. 1: 1-34.

44. Russell D A and Dong Z (1993) The affinities of a new theropod from the Alxa Desert, Inner Mongolia, People's Republic of China. Canadian Journal of Earth Sciences. 30: 2107-2127.

45. Charig A J and Milner A (1997) *Baryonyx walkeri*, a fish-eating dinosaur from the Wealden of Surrey. Bulletin of the Natural History Museum of London Geology Series 53(1): 11-70.

46. Makovicky P J, Apesteguia S and Agnolin F (2005) The earliest dromaeosaurid theropod from South America. Nature. 437: 1007-1011.

47. Bonaparte J F, Novas F E and Coria R A (1990) *Carnotaurus sastrei* Bonaparte, the horned, lightly built carnosaur from the ?Albian of Patagonia. Los Angeles Museum Contributions in Science. 416: 1-41.

48. Zhou Z and Wang X (2000) A new species of *Caudipteryx* from the Yixian Formation of Liaoning, Northeast China. Vertebrata Palasiatica. 38(2): 111-127.

49. Zhou Z, Wang X, Zhang F, Xu X (2000) Important features of *Caudipteryx*-Information from two nearly complete new specimens. Vertebrata Palasiatica. 38(4): 241-254.

50. Carpenter K, Miles C and Cloward K (2005) Redescription of the small maniraptoran theropods *Ornitholestes* and *Coelurus* from the Upper Jurassic Morrison Formation of Wyoming. In K. Carpenter Editor. The Carnivorous Dinosaurs. Indiana University Press. pp. 49-71.

51. Colbert E H (1989) The Triassic dinosaur *Coelophysis*. Bulletin of the Museum of Northern Arizona. 57: 1-174.

52. Ostrom J H (1969) Osteology of *Deinonychus antirrhopus*, an unusual theropod from the Lower Cretaceous of Montana. Peabody Museum of Natural History, Bulletin, 30: 1-165.

53. Novas F E, Pol D, Canale JI, Porfiri J D and Calvo J O (2009) A bizarre Cretaceous theropod dinosaur from Patagonia and the evolution of Gondwanan dromaeosaurids. Proceedings of The Royal Society B, 276, 1101-1107.

54. Welles S (1984) *Dilophosaurus wetherilli* (Dinosauria, Theropoda). Osteology and comparisons. Palaeontographica Abteilung A. 185: 85-180.

55. Zhang F, Zhou Z, Xu X, Wang X, Sullivan C (2008) A bizarre Jurassic maniraptoran from China with elongate ribbon-like feathers. Nature 455: 1105-1108.

56. Zanno L E (2006) The pectoral girdle and forelimb of the primitive therizinosauroid *Falcarius utahensis* (Theropoda, Maniraptora): Analyzing evolutionary trends within Therizinosauroidea. Journal of Vertebrate Paleontology. 26(3): 636-650.

57. Azuma Y and Currie P J (2000) A new carnosaur (Dinosauria: Theropoda) from the Lower Cretaceous of Japan. Canadian Journal of Earth Sciences. 37: 1735-1753.

58. Osmolska H, Roniewicz E and Barsbold R (1972) A new dinosaur, *Gallimimus bullatus* n. gen., n. sp. (Ornithomimidae) from the Upper Cretaceous of Mongolia. Acta Palaeontologica Polonica. 27: 103-143.

59. Xu X and Wang X (2004) A New Dromaeosaur (Dinosauria: Theropoda) from the Early Cretaceous Yixian Formation of Western Liaoning. Vertebrata Palasiatica. 42(2):111-119.

60. Sereno P C, Tan L, Brusatte, SL, Kreigstein, H J, Zhao X and Cloward K (2009) Tyrannosaurid skeletal design first evolved at small body size. Science. 326: 418-22.

61. Kobayashi Y and Barsbold R (2005). Anatomy of *Harpymimus okladnikovi* Barsbold and Perle 1984 (Dinosauria: Theropoda) of Mongolia. In K. Carpenter The Carnivorous Dinosaurs. Indiana University Press, pp. 97-126.

62. Novas F E (1994) New information on the systematics and postcranial skeleton of *Herrersaurus ischigualastensis* (Theropoda: Herrersauridae) from the Ischigualasto Formation (Upper Triassic) of Argentina. Journal of Vertebrate Paleontology. 13(4): 400-423.

63. Lu J (2004) Oviraptorid dinosaurs from Southern China. Ph.D Thesis: 1-265.

64. Hwang S, Norell M A, Ji Q and Gao K (2004) A large compsognathid for the Early Cretaceous Yixian Formation of China. Journal of Systematic Palaeontology. 2(1):13-30.

65. Ji Q, Ji S, Lu J, You H, Chien W Liu and Liu Y(2005) First avialian bird from China. Geological Bulletin of China. 24(3), 197-210.

66. Chiappe LM and Gühlich U B (2011) Anatomy of Juravenator starki (Theropoda: Coelurosauria) from the Late Jurassic of Germany. Neues Jahrbuch für Geologie und Paläontologie – Abhandlungen. 258: 277-296.

67. Xu, X, Clark JM, Mo J, Choiniere J, Forster C A, Erickson GM, Hone D W E, Corwin S, Eberth D, Nesbitt S, Zhao Q, Hernandez R , Jia C, Han F, Guo Y (2009). A Jurassic ceratosaur from China helps clarify avian digital homologies. Nature. 459: 940-944.

68. Turner A H, Pol D, Clarke J A, Erickson G A and Norell M A (2007) A basal dromaeosaurid and size evolution preceding avian flight. Science. 317: 1378-81.

69. Perle A, Chiappe L M, Barsbold R, Clarke J M and Norell M A (1994) Skeletal morphology of *Mononykus olecranus* (Theropoda: Avialae) from the Late Cretaceous of Mongolia. American Museum Novitates. 3105: 1-29.

70. Zanno L E, Gillette D D, Albright L B and Titus A L (2009) A new North American therizinosaurid and the role of herbivory in 'predatory' dinosaur evolution. Proceedings of the Royal Society B. 1672: 3505-3511.

71. Smith D (1992) The type specimen of *Oviraptor philoceratops*, a theropod dinosaur from the Upper Cretaceous of Mongolia. Neues Jahrbuch ftir Geologie und Paliontologie, Abhandlungen. 186: 365-388.

72. Allain R and Chure D J (2002) *Poekilopleuron bucklandii* , the theropod dinosaur from the Middle Jurassic (Bathonian) of Normandy. Palaeontology. 45(6):1107-1121.

73. Ji Q, Currie P J, Norell M A and Ji S (1998) Two feathered dinosaurs from Northeastern China. Nature. 393: 753-761.

74. Perle S (1979) Segnosauridae-A new family of Theropoda from the Lower Cretaceous of Mongolia (In Russian). Transactions(Sestnaya Sovetsko-Mongol'skaya eskpeditsia, Trudy) 45-55.

75. Gao Y (1998) A new species of Middle Jurassic Carnosauria from Dashanpu, Zigong, Sichaun Province, Szechuanosaurus zigongensis sp. nov. Vertebrata Palasiatica, 31(4): 308-314.

76. Carpenter K, Miles C and Cloward K (2005) New small theropod from the Upper Jurassic Morrison Formation of Wyoming. In K. Carpenter The Carnivorous Dinosaurs. Indiana University Press, pp. 23-48.

77. Britt B (1991) Theropods of Dry Mesa Quarry (Morrison Formation, Late Jurassic), Colorado, with emphasis on the osteology of *Torvosaurus tanneri*. Brigham Young University, Geological Studies. 1-72.

78. Xu X and Wang X (2003) A new maniraptoran from the Early Cretaceous Yixian Formation of western Liaoning. Vertebrata Palasiatica. 41(3): 195-202.

79. Chiappe L M, Ji S and Ji Q (2007) Juvenile Birds from the Early Cretaceous of China: Implications for Enantiornithine Ontogeny. American Museum Novitates. 3594(1): 1-46.

80. Zhou Z, Clarke J, and Zhang, F (2002) *Archeoraptor*'s better half. Nature. 420:285.

81. Sereno P C, Wilson J A, Larsson H C E, Dutheil D B and Sues H D (1994) Early Cretaceous dinosaurs from the Sahara. Science. 266: 267-271.

82. Hocknull S A, White M A, Tischler T R, Cook A G, Calleja N D, Sloan T and Elliott D (2009) New Mid-Cretaceous (Latest Albian) Dinosaurs from Winton, Queensland, Australia. PloS one, 4(7), e6190.

83. Xu X, Tang Z and Wang X L (1999) A therizinosauroid dinosaur with integumentary structures from China. Nature. 399: 350-354.

84. Madsen J H and Welles S (2000) *Ceratosaurus* (Dinosauria, Theropoda) a revised osteology. Miscellaneous Publications of the Utah Geological Survey. 2: 1-80.

85. Benson R B and Xu X (2008) The anatomy and systematic position of the theropod dinosaur *Chilantosaurus tashuikouensis* Hu,1964 from the Early Cretaceous of Alanshan, People's Republic of China. Geological Magazine. 145(6): 778-789.

86. Sereno P C, Dutheil D B, Iarochene M, Larsson H C E, Lyon G H, Magwene P M, Sidor C A,Varricchio D J and Wilson J A (1996) Predatory dinosaurs from the Sahara and Late Cretaceous faunal differentiation. Science. 272: 986-991.

87. Holtz T R (1995) The Arctometatarsalian pes, an unusual structure of the metatarsus of Cretaceous theropods (Dinosauria: Saurischia). Journal of Vertebrate Paleontology. 14(4): 480-519.

88. Sadleir R, Barret P M. and Powell H P (2008) The anatomy and systematics of *Eustreptospondylus oxoniensis*. A theropod dinosaur from the Middle Jurassic of Oxfordshire, England, London: The Paleaontographical Society.

89. Kobayashi Y and Barsbold R (2005) Re-examination of a primitive ornithomimosaur, *Garudimimus brevipes* Barsbold, 1981 (Dinosauria: Theropoda), from the Late Cretaceous of Mongolia. Canadian Journal of Earth Sciences. 42: 1501-1521.

90. Xu X, Tan Q, Wang J, Zhao X and Tan L (2007) A gigantic bird-like dinosaur from the Late Cretaceous of China. Nature, 447: 844-847.

91. Zhang X, Xu X, Zhao X, Sereno P C, Kuang X and Tan L (2001) A long-necked Therizinosauroid dinosaur from the Upper Cretaceous Iren Dabasu Formation of Nei Mongol, People's Republic of China. Vertebrata Palasiatica. 39(4): 282-290.

92. Brusatte S L, Benson R B and Hutt S (2008) The osteology of *Neovenator salerii* (Dinosauria: Theropoda) from the Wealden Group (Barremian) of the Isle of Wight, London: Palaeontographical Society. 1-75.

93. de Kirk W J, Forster C A, Sampson S, Chinsamy A, Ross C F (2000) A new coelurosaurian dinosaur from the Early Creaceous of South Africa. Journal of Vertebrate Paleontology. 20(2):324-332.

94. Karhu A A and Rautian A (1996) A new family of Maniraptora (Dinosauria: Saurischia) from the Late Cretaceous of Mongolia. Paleontological Journal. 30(5):583-592.

95. Ostrom J H (1981) *Procompsognathus*: Theropod or thecodont. Palaeontographica A, 179: 179-195.

96. Forster C A , Sampson, S, Chiappe L M and Krause D W (1998) The theropod ancestry of birds: New evidence from the Late Cretaceous of Madagascar. Science 279:1915-1919.

97. Farina R A and Christiansen P (2004) Mass Prediction in theropod dinosaurs. Historical Biology. 16(2); 85-92.

98. Tao H E, Wang X L and Zhou Z H (2008) A new genus and species of Caudipterid dinosaurs from the Lower Cretaceous Jiufotang Foramtion of Western Liaoning, China. Vertebrata Palasiatica. 46(3): 178-189.

99. Currie P and Zhao X (1993) A carnosaur (Dinosauria, Theropoda) from the Jurassic of Xinjiang, People's Republic of China. Canadian Journal of Earth Sciences. 30: 2037-2081.

100. Raath M A (1969) A new coelurosaurian dinosaur from the Forest Sandstone of Rhodesia. Arnoldia. 28(4): 1-25.

101. Rauhut O W and Xu X (2005) The small theropod dinosaurs *Tugulusaurus* and *Phaedrolosaurus* from the Early Cretaceous of Xinjiang, China. Journal of Vertebrate Paleontology. 25(1): 107-118.

102. Norell M A and Makovicky P J (1999) Important features of the Dromaeosaurid Skeleton II: Information from newly collected specimens of *Velociraptor mongoliensis*. American Museum Novitates. 3282: 1-45.

103. Dong Z, Zhou S and Zhang Y (1983) Dinosaurs from the Jurassic of Sichuan. Palaeontologica Sinica Series C. 162(23): 1-136.

104. Osmolska H and Roniewicz E (1970) Deinocheiridae, a new family of theropod dinosaurs. Acta Paleontologica Polonica. 21: 5-19.

105. Barsbold R, Osmolska H and Kurzanov S M (1987) On a new troodontid (Dinosauria, Theropoda)from the Early Cretaceous of Mongolia. Acta Palaeontologica Polonica. 32(1-2): 121-132.

106. Xu X, Zhang X, Sereno P C, Zhao XKuan X, Han J and Tan L (2002) A new Therizinosauroid (Dinosauria, Theropoda) from the Upper Cretaceous Iren Dabasu Formation of Nei Mongol. Vertebrata Palasiatica. 40(3): 228-240.

107. Zanno L E and Sampson S D (2005) A new oviraptorosaur (Theropoda, Maniraptora) from the Late Cretaceous (Campanian) of Utah. Journal of Vertebrate Paleontology. 25(4): 897-904.

108. Sereno P C (1994) The Pectoral Girdle and Forelimb of the Basal Theropod *Herrerasaurus Ischigualastensis*. Journal of Vertebrate Paleontology. 13(4): 425-450.

109. Calvo J O, Porfiri J D, Veralli C, Novas F and Poblete F (2004) Phylogenetic status of *Megaraptor namunhuaiquii* Novas based on a new specimen from Neuquén, Patagonia, Argentina. Ameghiniana. 41: 565-575.

110. Osmolska H (1981) Co-ossified tarsometatarsi in theropod dinosaurs and their bearing on the problem of bird origins. Acta Palaeontologia Polonica. 42: 79-95.

111. Xu X, Choiniere J N, Pittman M, Tan Q, Xiao D, Li Z, Tan L, Clark J M, Norell M A, Hone D W E and Sullivan C (2010) A new dromaeosaurid (Dinosauria: Theropoda) from the Upper Cretaceous Wulansuhai Formation of Inner Mongolia, China. Zootaxa 2403: 1-9.

112. Sternberg C M (1932) Two new theropod dinosaurs from the Belly River Formation of Alberta. Canadian Field Naturalist. 46(5): 99-105.
